# Design, Synthesis, and Biological Evaluation of Tetrahydroindazole-Based Sulfonamides as Potential Multi-Target Anti-Inflammatory Agents

**DOI:** 10.3390/ph19060843

**Published:** 2026-05-28

**Authors:** Mohammed A. I. Elbastawesy, Ahmed H. Abdelhafez, Abdullah Yahya Abdullah Alzahrani, Bandar A. Alyami, Hanyu Ling, Mahmoud S. Abdelbaset, Ahmed A. Gaber, Bahaa G. M. Youssif, Stefan Brase, Hiroyuki Konno

**Affiliations:** 1 Department of Pharmaceutical Organic Chemistry, Faculty of Pharmacy, Al-Azhar University, Assiut 71524, Egypt; mohamedali.pharm.ast@azhar.edu.eg (M.A.I.E.); mahmoudsobhyorganic@yahoo.com (M.S.A.); 2 PharmaHelp Foundation Inc., Falls Church, VA 22046, USA; ahmed@pharmahelp.org; 3 Department of Chemistry, Faculty of Science, King Khalid University, Abha 61413, Saudi Arabia; ayalzahrani@kku.edu.sa; 4 Department of Pharmaceutical Chemistry, College of Pharmacy, Najran University, Najran 11001, Saudi Arabia; bandaralyami1@gmail.com; 5 Department of Chemistry and Biological Engineering, Graduate School of Science and Engineering, Yamagata University, Yonezawa, Yamagata 992-8510, Japan; t246246d@st.yamagata-u.ac.jp; 6 Department of Pharmaceutical Organic Chemistry, Faculty of Pharmacy (Boys), Al-Azhar University, Cairo 11884, Egypt; nouralrahmani@gmail.com; 7 Pharmaceutical Organic Chemistry Department, Faculty of Pharmacy, Assiut University, Assiut 71526, Egypt; bgyoussif2@gmail.com; 8 Institute of Biological and Chemical Systems, IBCS-FMS, Karlsruhe Institute of Technology, 76131 Karlsruhe, Germany

**Keywords:** sulfonamide, chalcone, anti-inflammatory COX-2, 5-LOX, sEH, cardioprotective properties

## Abstract

**Background/Objectives**: The dual inhibition of the COX-2 and 5-LOX pathways, in addition to sEH inhibition, presents a superior approach to managing inflammation while mitigating the cardiovascular adverse effects typically associated with conventional NSAIDs. These multi-target agents are safer and more efficient as they inhibit the synthesis of pro-inflammatory leukotrienes while preserving cardioprotective epoxyeicosatrienoic acids. **Methods**: This study reports the development of multi-target inhibitors to mitigate inflammatory and cardiovascular conditions. We examined a series of tetrahydroindazole-sulfonamide hybrids (**3a**–**g** and **4a**–**e**) against the enzymes COX-1/2, 5-LOX, and sEH. **Results**: Compound **3b** outperformed celecoxib as a multi-target agent, inhibiting COX-2 (IC_50_ = 0.08 µM, SI = 82), 5-LOX (IC_50_ = 0.46 µM), and sEH (IC_50_ = 21.95 nM) in many metrics. In cellular experiments, **3b** showed strong cardioprotective and anti-inflammatory effects, significantly reducing TNF-α (65.58%), LDH (76.26%), and CK-MB (76.76%) levels compared to LPS-treated controls. Molecular docking validated these findings, indicating that **3b** was comparable to celecoxib at the COX-2 site via a thorough six hydrogen-bond network and achieves considerable sEH affinity through specialized halogen bonding and aromatic stacking. These results indicate that **3b** effectively provides dual anti-inflammatory and cardioprotective effects. **Conclusions**: Our findings suggest that targeting the COX/5-LOX/sEH pathways simultaneously offers a balanced multi-target profile for treating complex inflammatory diseases while minimizing cardiovascular risks.

## 1. Introduction

Selective cyclooxygenase-2 (COX-2) inhibitors, or coxibs, are a specialized class of nonsteroidal anti-inflammatory drugs (NSAIDs) designed to treat pain and inflammation with fewer gastrointestinal side effects than traditional NSAIDs [1,2]. NSAIDs work by blocking the production of prostaglandins, the compounds responsible for signaling pain [3]. While traditional NSAIDs inhibit both COX-1 and COX-2 enzymes, coxibs specifically target COX-2. This is a critical distinction because COX-1 helps protect the stomach lining and supports blood clotting, whereas COX-2 is primarily produced at sites of tissue injury. By selectively inhibiting COX-2, these medications provide necessary relief while reducing the risk of gastric ulcers and bleeding [4,5].

Currently, celecoxib (Celebrex) is the only selective COX-2 inhibitor authorized for use in the United States, where it is primarily prescribed for osteoarthritis, rheumatoid arthritis, and acute pain. While other drugs in this class, such as etoricoxib and parecoxib, remain available in various international markets, they are not approved for use in the U.S. Safety concerns have led to the withdrawal of several prominent coxibs from the global market [6,7]. Rofecoxib (Vioxx) was voluntarily removed in 2004 following evidence of an increased risk of myocardial infarction (heart attack) and stroke. Similarly, valdecoxib (Bextra) was withdrawn in 2005 due to cardiovascular hazards and reports of rare but severe dermatological reactions [8,9].

Selective COX-2 inhibitors carry a significant risk of cardiotoxicity due to an imbalance in vascular signaling. Under normal conditions, COX-2 produces prostacyclin (PGI2), a vital vasodilator that prevents blood clots. By selectively blocking COX-2, these drugs suppress PGI2 while leaving thromboxane (TXA2), a potent vasoconstrictor and clotting agent produced by COX-1, unaffected. This shift toward a pro-thrombotic state can lead to elevated blood pressure and a heightened risk of myocardial infarction (heart attack) and cerebrovascular accidents (stroke) [10,11].

Dual inhibition of COX-2 and soluble epoxide hydrolase (sEH) offers a promising strategy to neutralize the cardiotoxicity traditionally associated with coxibs. While COX-2 inhibition alone can create a pro-thrombotic environment, sEH inhibition counters this by preserving epoxyeicosatrienoic acids (EETs), lipid mediators that promote vasodilation and prevent clotting. By blocking the sEH enzyme, which normally degrades EETs into less active metabolites, these dual inhibitors increase the bioavailability of cardioprotective compounds [12]. This synergy not only restores vascular balance but also enhances anti-inflammatory effects by suppressing pathways like NF-κB. Ultimately, this multi-target approach maximizes therapeutic efficacy in inflammatory diseases while significantly reducing the risk of systemic and cardiovascular damage [6,13].

Alternatively, the lipoxygenase (LOX) pathway converts membrane-derived arachidonic acid (AA) into leukotrienes (LTs), which serve as potent mediators of the inflammatory response [14]. The simultaneous inhibition of COX-2 and 5-LOX offers a comprehensive therapeutic approach by concurrently suppressing the production of both prostaglandins and leukotrienes [15]. Since both pathways compete for the same AA substrate, dual inhibition prevents the “leukotriene shunt”, a phenomenon where shunted AA metabolism increases inflammatory LTs, thereby enhancing anti-inflammatory efficacy. This integrated strategy represents a compelling framework for developing next-generation anti-inflammatory agents with an optimized safety and efficacy profile [16,17].

The FDA has not yet authorized any medications that simultaneously inhibit COX-2, 5-LOX, and sEH. While studies have investigated these targets individually or in pairs, a comprehensive therapeutic drug that incorporates all three is not currently available on the market [18]. A triple inhibitor targeting COX-2, 5-LOX, and sEH is a more advanced approach to addressing the challenges encountered with previous anti-inflammatory therapies. These compounds are more effective than conventional anti-inflammatory medications because they simultaneously inhibit COX-2 and 5-LOX. This dual blockade inhibits the “leukotriene shunt,” a process in which the body overproduces inflammatory leukotrienes to compensate for the deficiency of prostaglandins. This may result in persistent pain and gastric irritation. The triple inhibitor halts both routes at their metabolic origin, enhancing its efficacy against a broader spectrum of inflammatory mediators [19,20].

Furthermore, the integration of sEH inhibition serves as a critical safety mechanism to neutralize the cardiotoxic risks inherent in selective COX-2 inhibition [7]. Common coxibs disrupt vascular equilibrium and promote thrombosis, but inhibiting sEH preserves cardioprotective epoxyeicosatrienoic acids (EETs). These mediators facilitate healthy vasodilation and exhibit anti-thrombotic properties, thereby safeguarding the cardiovascular system from the adverse consequences of prostaglandin inhibition. This multi-targeted logic signifies a significant transformation in drug development. It provides a singular treatment agent that effectively addresses safety issues for both cardiac and gastric health [6].

### Rational Design

**Figure 1:**
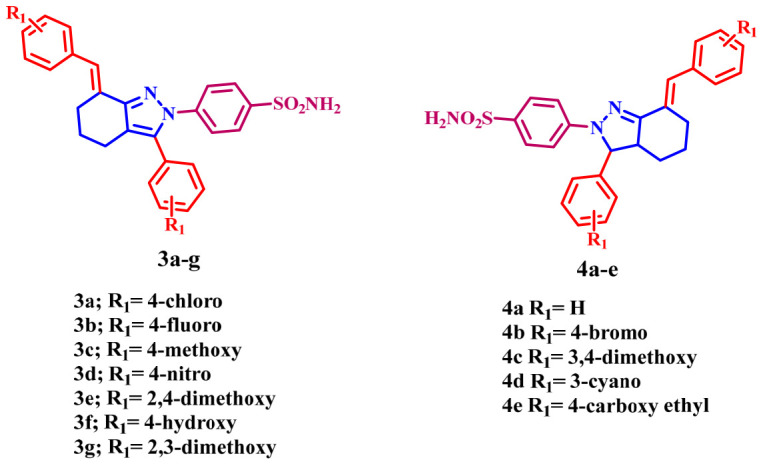
Structures of newly developed compounds **3a**–**g** and **4a**–**e**.

In response to the therapeutic need for safer anti-inflammatory agents, we synthesized a new series of tetrahydroindazole-sulfonamide hybrids (**3a**–**g** and **4a**–**e**, Figure 1).

The new compounds are categorized into two groups based on their structures: Tetrahydroindazole derivatives (**3a**–**g**), which comprise seven derivatives, with the substituent R_1_ varying in position and functional groups, such as 4-chloro, 4-fluoro, and various methoxy/hydroxy substituents. The benzenesulfonamide moiety, a common pharmacophore in medicinal chemistry, is present in all molecules in this series. The second series (**4a**–**e**) has five compounds with a hexahydroindazole scaffold. This structural modification most likely alters the molecule’s three-dimensional orientation relative to the tetrahydro analogs. The R_1_ substituents in this series range from simple hydrogen (**4a**) to more complex groups such as 3,4-dimethoxy (**4c**) and 4-carboxyethyl.

This series of compounds was particularly designed to function as a triple-action inhibitor of COX-2, 5-LOX, and sEH. The benzenesulfonamide moiety is a characteristic pharmacophore that ensures the compound selectively targets COX-2 [21]. The 4,5,6,7-tetrahydroindazole core is a robust, adaptable framework that facilitates the optimal orientation of the diaryl substituents, thereby enhancing interactions within the 5-LOX and sEH binding sites [22,23].

Our strategy focuses on tailoring this molecular architecture to achieve balanced potency across all three pathways, so addressing the ‘leukotriene shunt’ and mitigating cardiotoxic hazards within a single pharmaceutical entity. This paper presents the design, synthesis, and biological evaluation of tetrahydroindazole-sulfonamide hybrids, highlighting their potential as next-generation treatments for complex inflammatory diseases.

## 2. Results and Discussion

### 2.1. Chemistry

**Scheme 1:**
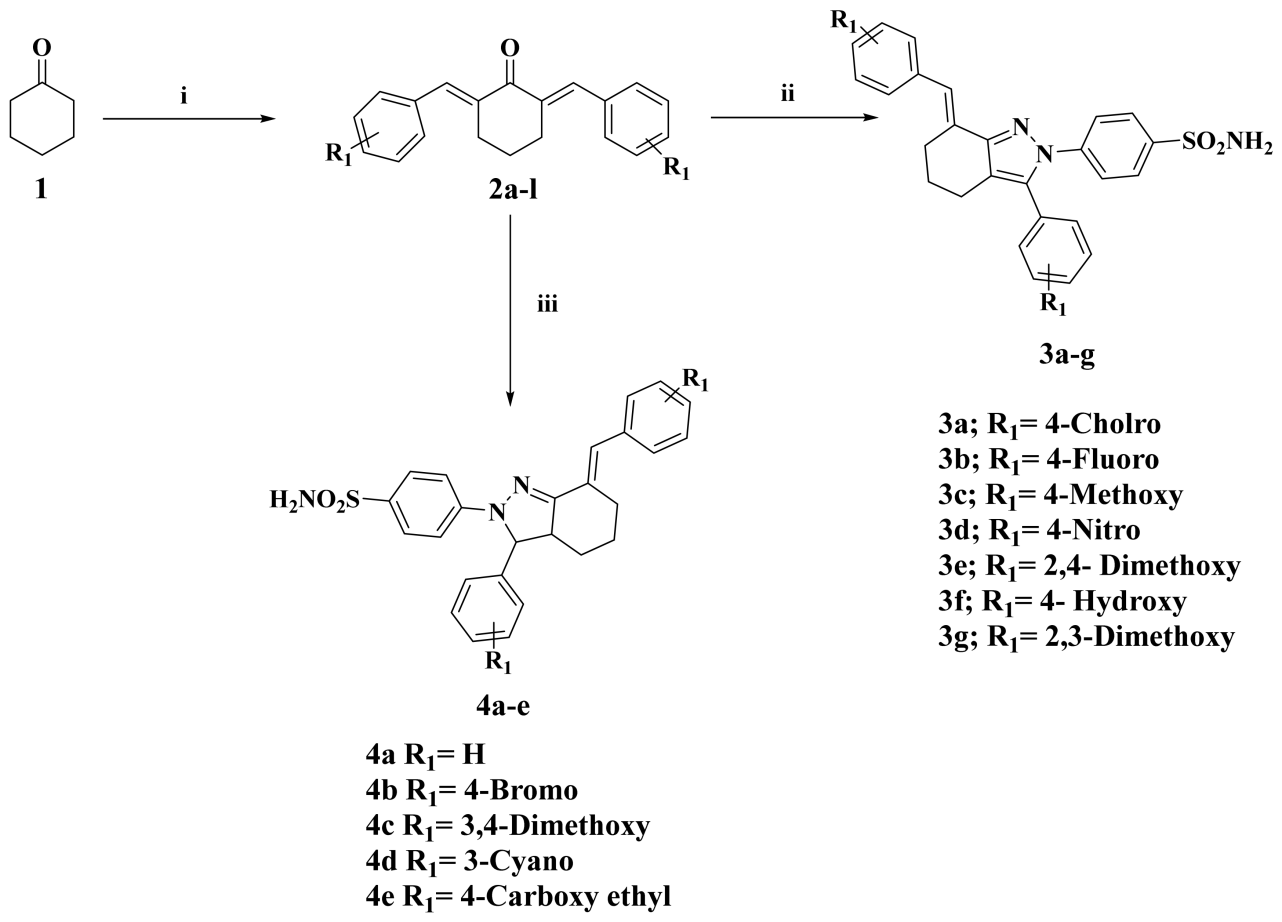
Synthesis of target compounds **3a**–**g** and **4a**–**e**.

In this study, several new indazole/sulfonamide derivatives (**3a**–**g** and **4a**–**e**) were synthesized from benzylidene cyclohexanone precursors (**2a**–**l**), as detailed in Scheme 1. Compounds **2a**–**l** were synthesized via acid-catalyzed condensation of cyclohexanone with various benzaldehydes (1:2 molar ratio) in refluxing absolute ethanol. The resulting 2,6-bis((*E*)-benzylidene)cyclohexan-1-one derivatives (**2a**–**l**) were characterized, and their physical and spectral data were fully consistent with previously reported values [24,25].

**Reagent and reaction conditions:** (i) 2 Equiv. of different aldehydes, conc. HCl, ethanol, reflux 10–12 h, 78–91% (ii) Hydrazine sulfanilamide, Sodium acetate, acetic acid, reflux 30–36 h (iii) Hydrazine sulfanilamide, piperidine, reflux 16–20 h.

Seven bis-chalcones were reacted with sulfanilamide in ethanol, using acetic acid and sodium acetate to yield pyrazole derivatives **3a**–**g** in good yields. Conversely, the partially saturated indazole targets **4a**–**e** were synthesized from the same chalcone and sulfonamide precursors using piperidine as the cyclization catalyst, as illustrated in Scheme 1.

The structures of all new compounds were elucidated using ^1^H NMR, ^13^C NMR, LC-MS, and HRMS. In the ^1^H NMR spectra of **3a**–**g** and **4a**–**e**, the sulfonamide (NH_2_) protons appeared as a characteristic signal at δ 7.05 ppm, which disappeared upon D_2_O exchange. The aromatic protons of the three phenyl rings were observed as a series of multiplets and doublets within the δ 7.91–6.97 ppm range. Furthermore, ^13^C NMR analysis confirmed the proposed frameworks, with key signals at approximately δ 135–139 ppm and δ 145 ppm corresponding to the C=C and C-SO_2_ carbons, respectively.

As a representative example of the pyrazole series **3a**–**g**, the ^1^H NMR spectrum of compound **3a** displayed twelve aromatic protons as doublets between δ 7.94 and 7.18 ppm, consistent with three *p*-disubstituted phenyl rings. The sulfonamide protons were observed as a characteristic two-proton singlet at δ 7.49 ppm. Furthermore, the identity of **3a** was confirmed by HRESI-MS, which showed a molecular ion at m/z 510.0822 [M+H]^+^, in close agreement with the calculated value of 510.0810 for the formula C_26_H_21_Cl_2_N_3_O_2_S.

As a representative example of the partially saturated indazole series, the ^1^H NMR spectrum of compound **4e** displayed two doublet-of-doublets integrating to eight protons, consistent with two *p*-disubstituted aryl groups. A quartet at δ 4.30 ppm, corresponding to four protons, was assigned to the methylene groups of the ethyl ester moieties. The ^13^C NMR data further supported the structure, with ester carbonyl signals at δ 165.95 and 165.91 ppm and ethyl group signals at δ 57.16 and 14.70 ppm. Finally, HRESI-MS analysis of **4e** showed a molecular ion at *m*/*z* 610.1986 M+Na]^+^, which is in excellent agreement with the formula C_32_H_33_N_3_O_6_S.

### 2.2. Biology

#### 2.2.1. In Vitro Cyclooxygenase (COX) Inhibition Assay

Compounds **3a**–**g** and **4a**–**e** were evaluated in vitro for their inhibitory potential against COX-1 and COX-2 isozymes. Isozyme-specific inhibition was screened using a colorimetric enzyme immunoassay (EIA) kit [7], with potency expressed as the concentration required to achieve 50% inhibition (IC_50_). Additionally, COX-2 selectivity indices (SI) were calculated as the IC_50_ (COX-1)/IC_50_ (COX-2) ratio and compared with the reference drug celecoxib. The resulting data are summarized in Table 1.

As shown in Table 1, compounds **3a**–**g** and **4a**–**e** exhibited moderate to weak inhibitory activity against COX-1, with IC_50_ values ranging from 0.84 to 16.47 μM. These results demonstrate lower potency relative to the reference standard, indomethacin, which yielded an IC_50_ of 1.11 μM. Among the series, compounds **3g** (R_1_ = 2,3-dimethoxy, scaffold A) and **4e** (R_1_ = 3-cyano, scaffold B) emerged as the most potent COX-1 inhibitors, with IC_50_ values of 0.84 μM and 1.22 μM, respectively. Their inhibitory profiles are comparable to that of the reference drug, indomethacin (IC_50_ = 1.11 μM). Compound **3c** (R_1_ = 4-methoxy, scaffold A) displayed the third-highest COX-1 inhibitory activity, with an IC_50_ value of 1.76 μM, representing a 1.5-fold reduction in potency compared to indomethacin. The remaining derivatives exhibited moderate to poor efficacy, with potencies ranging from 3.5- to 15-fold lower than the reference standard.

As indicated in Table 1, compounds **3a**–**g** and **4a**–**e** displayed significant inhibitory activity against the COX-2 isoform. Notably, these derivatives demonstrated greater potency toward COX-2 (IC_50_ = 0.08–5.92 μM) than COX-1 (IC_50_ = 0.84–16.47 μM), resulting in selectivity indices (SI) of up to 82. Except for compound **3b**, the observed COX-2 inhibitory activities for all derivatives were lower than that of celecoxib (IC_50_ = 0.11 μM). Among the series, compounds **3a**, **3b**, **3f**, and **4d** were identified as the most effective inhibitors, yielding IC_50_ values of 0.27, 0.08, 0.89, and 1.09 μM, respectively. Compound **3b** (R_1_ = 4-fluoro, scaffold A) emerged as the most potent COX-2 inhibitor in this study, with an IC_50_ value of 0.08 μM and a selectivity index (SI) of 82. This derivative demonstrated comparable efficacy to the reference drug celecoxib, which recorded an IC_50_ value of 0.11 μM and an SI of 42.

The substitution pattern at the C4 position of the benzylidene phenyl ring or the phenyl ring at the C3 position of the pyrazole moiety (R_1_ group) significantly modulates the COX-2 inhibitory activity of series **3a**–**g** and **4a**–**e**. For instance, although compounds **3d** (R_1_ = 4-NO_2_), **3e** (R_1_ = 2,4-dimethoxy), and **3g** (R_1_ = 2,3-dimethoxy) share scaffold A with **3b**, their distinct benzylidene phenyl or C3-pyrazole phenyl substituents lead to a marked reduction in efficacy. These derivatives yielded IC_50_ values of 5.92, 4.17, and 4.25 µM, respectively, representing at least a 52-fold decrease in potency compared to **3b**. These data indicate that the introduction of either a nitro group or dimethoxy substitutions (at the 2,4- or 2,3-positions) on the phenyl ring is detrimental to potency, suggesting the COX-2 binding site poorly tolerates these specific electronic or steric profiles.

Compounds **3a** (R_1_ = 4-Cl), **3c** (R_1_ = 4-OMe), and **3f** (R_1_ = 4-OH), which share the common scaffold A with **3b**, retained significant COX-2 inhibitory activity. These derivatives yielded IC_50_ values of 0.27, 1.37, and 0.89 µM, respectively. While these values represent a 3- to 17-fold reduction in potency relative to **3b**, they demonstrate that chloro, methoxy, and hydroxyl substituents are well tolerated at this position, with inhibitory potency following the order: Cl > OH > OMe.

Within series **4a-e** (scaffold B), compounds **4a** (R_1_ = H), **4b** (R_1_ = 4-Br), and **4c** (R_1_ = 3,4-dimethoxy) exhibited moderate COX-2 inhibitory efficacy, with IC_50_ values of approximately 2 µM. In contrast, the 4-carboxyethyl derivative **4e** showed weak activity (IC_50_ = 5.60 µM), representing a 70-fold reduction in potency relative to **3b**. While compound **4d** (R_1_ = 3-CN) emerged as the most potent derivative in this series (IC_50_ = 1.09 µM), it was still 13-fold less active than **3b** and exhibited a poor selectivity index (SI) of 1.2.

#### 2.2.2. In Vitro Lipoxygenase (5-LOX) Inhibition Assay

The series **3a**–**g** and **4a**–**e** were further evaluated for their inhibitory potential against 5-lipoxygenase (5-LOX) using a commercial assay kit, with Zileuton as the reference standard [26]. The resulting data, summarized in Table 1, align with the findings from the in vitro COX-1/COX-2 inhibition assays. Notably, compounds **3a**, **3b**, **3c**, and **3f** emerged as the most potent inhibitors within the series, yielding IC_50_ values of 0.80, 0.46, 0.87, and 0.93 µM, respectively, comparable to the IC_50_ of 0.69 µM observed for Zileuton.

Compound **3b** (R_1_ = 4-fluoro, scaffold A), previously identified as the most potent COX-2 inhibitor in the series, also demonstrated superior 5-LOX inhibition with an IC_50_ of 0.46 ± 0.02 µM. This activity surpassed the reference standard, Zileuton (IC_50_ = 0.69 ± 0.03 µM), making compound **3b** approximately 1.5 times more potent as a 5-LOX inhibitor. Furthermore, its dual-action profile is highlighted by a high selectivity index (SI) of 82 for COX-2.

Compounds **3a** (R_1_ = 4-Cl), **3c** (R_1_ = 4-OMe), and **3f** (R_1_ = 4-OH) exhibited significant 5-LOX inhibitory activity with IC_50_ values below 1 µM (0.80, 0.87, and 0.93 µM, respectively), performing comparably to Zileuton. These results indicate that chlorine atom, methoxy, and hydroxyl substitutions at the C4 of the phenyl rings in the benzylidene moiety or at C3 of the pyrazoline ring are well tolerated and maintain effective dual-inhibitory action against both COX-2 and 5-LOX.

Compounds **3g** (R_1_ = 2,3-dimethoxy, Scaffold A) and **4c** (R_1_ = 3,4-dimethoxy, Scaffold B) display moderate 5-LOX inhibitory activity with IC_50_ values of 1.10 and 1.68 µM, respectively, representing a 1.6-fold and 2.5-fold decrease in potency compared to Zileuton. The remaining compounds in the series exhibited weak 5-LOX inhibitory activity, with IC_50_ values ranging from 2.87 to 7.34 µM, reflecting at least a four-fold reduction in potency relative to the reference standard. These in vitro assays demonstrate that compounds **3a**, **3b**, and **3f** possess potent COX-2 inhibitory activity and favorable selectivity profiles, effectively functioning as dual COX-2/5-LOX inhibitors.

#### 2.2.3. In Vitro Soluble Epoxide Hydrolase (sEH) Assay

A cell-based assay experiment was employed to assess the inhibitory activity of compounds **3a**, **3b**, and **3f**, the most selective COX-2 inhibitors, against the sEH enzyme in vitro. AUDA and celecoxib were used as the reference drugs [6]. The IC_50_ values (nM) are presented in Table 1.

**Figure 2:**
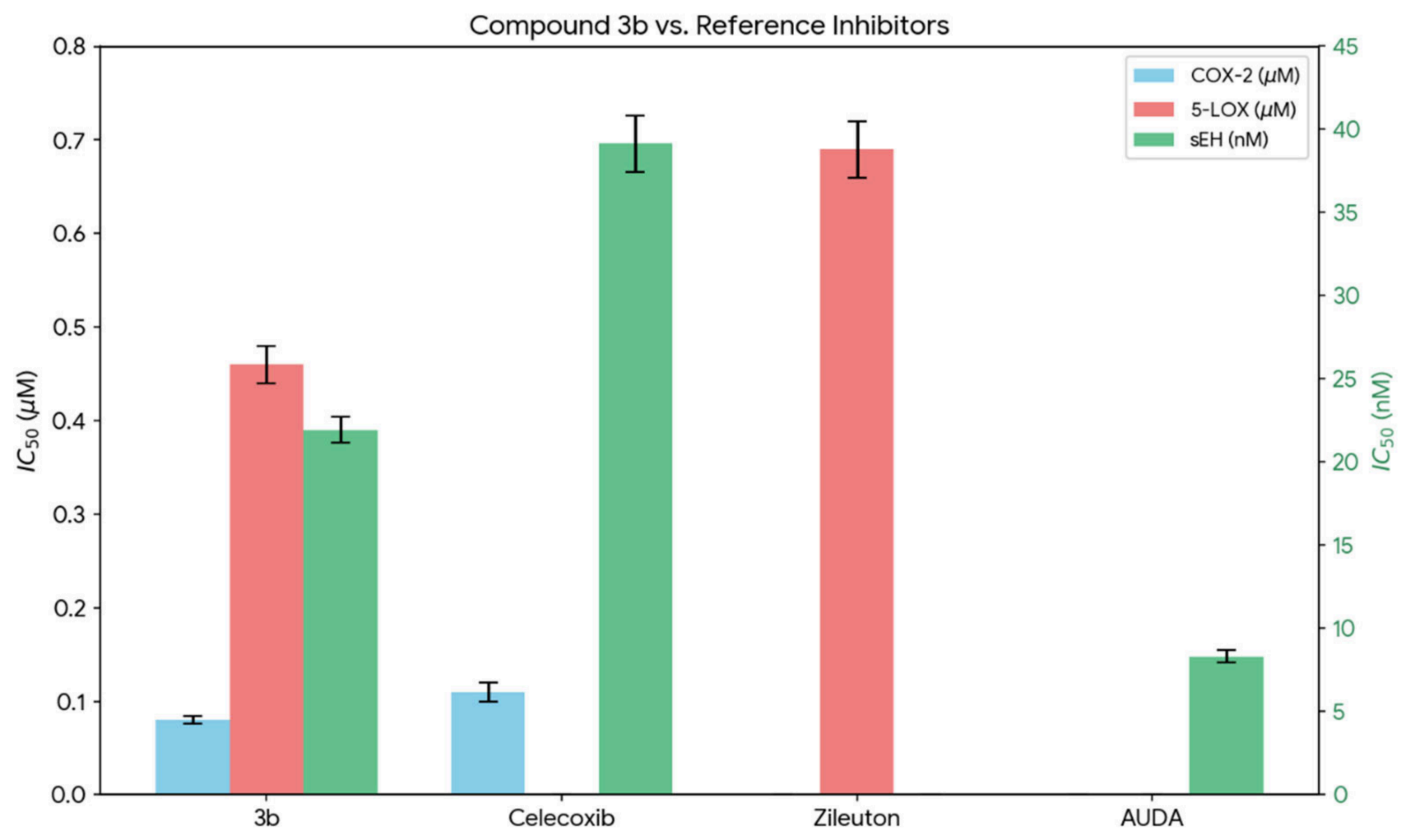
Inhibitory potency (IC_50_) of multi-target compound **3b** compared to reference inhibitors Celecoxib, Zileuton, and AUDA.

The tested compounds inhibited sEH, with IC_50_ values ranging from 21.95 to 66.29 nM. Although these values indicate lower potency relative to AUDA (IC_50_ = 8.32 nM), compounds **3a** and **3b** outperformed the second reference drug, celecoxib (IC_50_ = 39.15 nM), in terms of enzymatic suppression (Figure 2). Among the series, compounds **3a** (R_1_ = 4-Cl) and **3b** (R_1_ = 4-fluoro) demonstrated significant sEH inhibitory activity, with IC_50_ values of 27.56 and 21.95 nM, respectively. These analogs were 1.4- and 1.8-fold more potent than celecoxib (IC_50_ = 39.15 nM), though they remained at least 2.6-fold less potent than the reference standard AUDA (IC_50_ = 8.32 nM).

In contrast, compound **3f** (R_1_ = 4-OH) emerged as the least potent sEH inhibitor in the series, with an IC_50_ of 66.29 nM. This derivative was 1.7-fold less potent than celecoxib and approximately 8-fold less potent than the reference standard AUDA. These data indicate that compounds **3a** and **3b** function as multi-target COX-2/5-LOX/sEH inhibitors. Given their high selectivity indices (33 and 82, respectively), these dual-acting agents hold potential as anti-inflammatory leads with a reduced risk of cardiotoxicity.

#### 2.2.4. Modulation of TNF-α, CK-MB, and LDH

In vivo evaluation of serum biomarkers in rat models yields essential evidence for the multi-targeted anti-inflammatory effectiveness and safety profiles of compounds **3a**, **3b**, and **3f** as multi- COX-2/5-LOX/sEH inhibitors. The compounds’ ability to inhibit the major proinflammatory “cytokine-mediated hyperinflammation” is demonstrated by the significant reduction in Tumor Necrosis Factor Alpha (TNF-α) levels. This successfully halts the signaling cascade that typically induces irreversible tissue damage [27]. Moreover, a reduction in Creatine Kinase MB (CK-MB) and D-Lactate Dehydrogenase (D-LDH) is a crucial indicator of lower collateral organ damage [28,29]. While elevated CK-MB and LDH levels are traditional indicators of myocardial and systemic cellular injury that are the result of oxidative stress and inflammatory metabolic reprogramming, their normalization implies that these dual- or triple-target inhibitors provide a superior cardioprotective profile than traditional selective COX-2 inhibitors, which are frequently linked to cardiovascular risks [30,31].

Compounds **3a**, **3b**, and **3f,** identified as the most potent candidates in vitro, were selected for further evaluation of their effects on serum TNF-α, LDH, and CK-MB levels. To induce a systemic inflammatory response and tissue injury, mice received a single intraperitoneal (IP) injection of Lipopolysaccharide (LPS) [32]. Serum concentrations of the targeted markers were subsequently quantified using commercial ELISA kits, following the manufacturers’ standardized protocols [33]. The results were cited in Table 2.

The serum levels of TNF-α demonstrate that all three pyrazole derivatives **3a**, **3b**, and **3f** possess significant anti-inflammatory activity, substantially suppressing the LPS-induced cytokine flow (110.40 pg/mL) toward the baseline level of the normal control (20.85 pg/mL). Of the evaluated compounds, **3b** (serum level = 51.67 pg/mL) demonstrated the highest potency, followed by **3a** (59.36 pg/mL), both displaying inhibitory effects comparable to the reference medication, celecoxib (41.75 pg/mL, 76.66% inhibition; Table 2). Specifically, compound **3b** demonstrated a 65.58% inhibition, maintaining roughly 85% of the reference drug’s efficacy, whereas compounds **3a** and **3f** (69.04 pg/mL) exhibited 57.00% and 46.19% inhibition, respectively. The compound-specific inhibition of TNF-α highlights the ability of these derivatives to disrupt the initial inflammatory cascade triggered by LPS, confirming the significant therapeutic potential of this multi-targeted scaffold as an anti-inflammatory medicine.

The assessment of LDH levels further validated the protective effects of the synthesized compounds against LPS-induced systemic cellular damage. The positive control group showed a substantial increase in LDH levels (244.55 mIU/mL) compared with the normal control (88.73 mIU/mL), indicating severe tissue hypoxia and necrosis. Remarkably, all evaluated compounds significantly mitigated this increase. Compound **3b** (125.72 mIU/mL) showed superior efficacy, exceeding the performance of celecoxib (150.05 mIU/mL) with a 76.26% reduction in the increase in LDH. Compound **3a** (146.03 mIU/mL) exhibited enhanced activity relative to the reference medication, achieving 63.23% inhibition, whilst **3f** (174.32 mIU/mL) attained a commendable 45.07% inhibition. The substantial decrease in serum LDH, especially at **3b** and **3a**, indicates that these multi-target inhibitors provide superior cytoprotection and more effectively maintain metabolic integrity during inflammatory stress compared to selective COX-2 inhibition alone.

**Figure 3:**
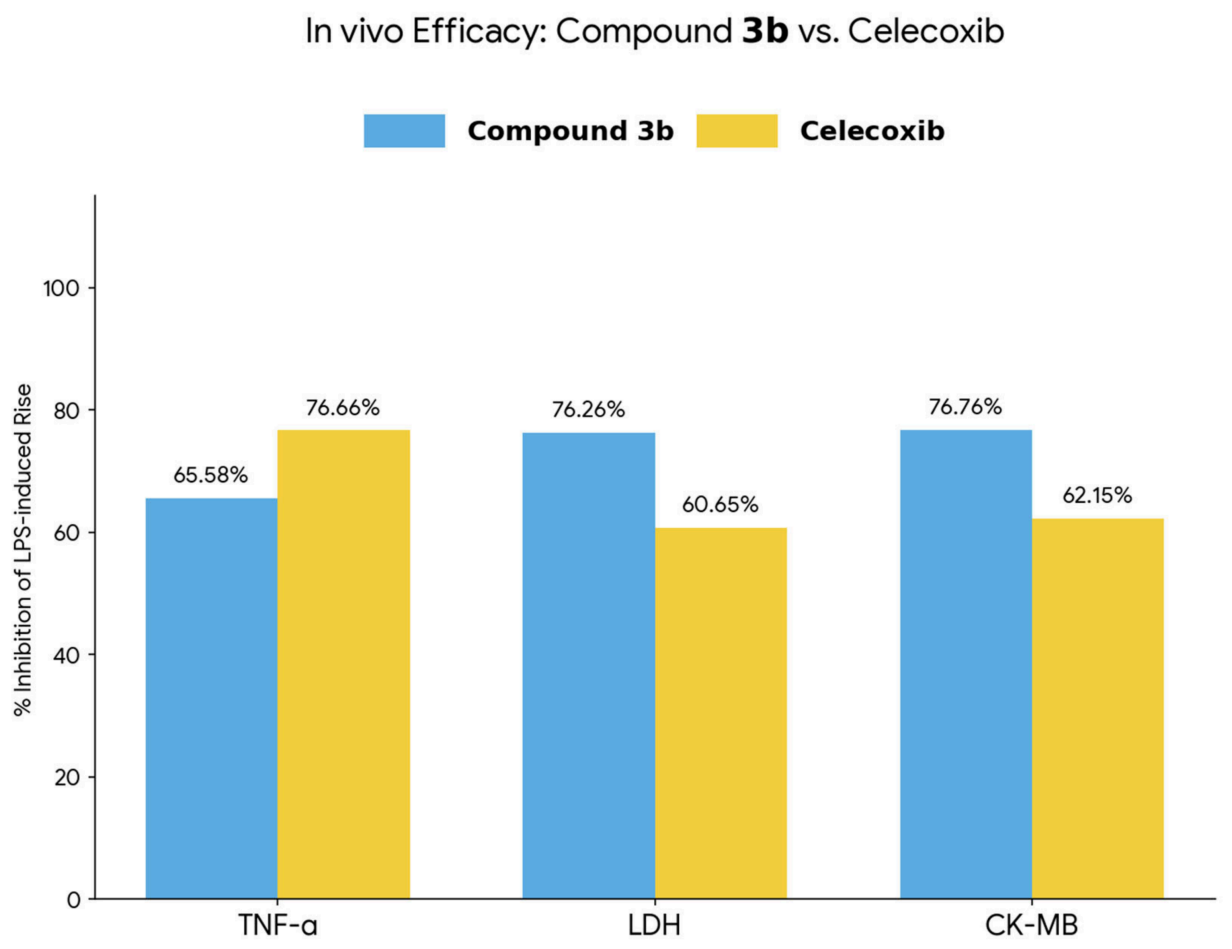
In vivo inhibitory efficacy of compound **3b** vs. celecoxib.

The evaluation of CK-MB levels provides additional evidence of the compounds’ capacity to alleviate inflammation-induced tissue damage. While the positive control group showed a substantial rise in CK-MB levels to 6.78 ng/mL compared to the normal control (1.10 ng/mL), treatment with the synthesized derivatives led to a marked reduction in this marker of cellular injury. Compound **3b** (2.42 ng/mL) had the most significant protective effect, surpassing celecoxib (3.25 ng/mL) with an inhibition rate of 76.76%, compared to the reference drug’s 62.15%. Compounds **3a** (4.25 ng/mL) and **3f** (6.08 ng/mL) yielded inhibition rates of 44.54% and 12.32%, respectively. The exceptional efficacy of **3b** in reducing CK-MB levels, surpassing that of celecoxib, underscores the potential benefits of the triple-inhibition strategy targeting COX-2, 5-LOX, and sEH in achieving superior cardioprotective and cytoprotective effects during systemic inflammation (Figure 3).

### 2.3. Docking Study

#### 2.3.1. Analysis of 3b and Celecoxib with the COX-2 Active Site

To understand the binding mechanism of the most selective COX-2 inhibitor, compound **3b**, we performed docking analysis using AutoDock 4.2 [31,32] and Discovery Studio 2024 [34]. We docked compound **3b** and the reference drug celecoxib into the COX-2 crystal structure (PDB ID: 3LN1) [35], selecting the most stable models based on the highest-scoring conformations. To validate the protocol, we re-docked celecoxib into the active site; the resulting pose closely matched the original crystal structure with a root-mean-square deviation (RMSD) of 1.098 Å, confirming the reliability of our docking parameters.

**Figure 4:**
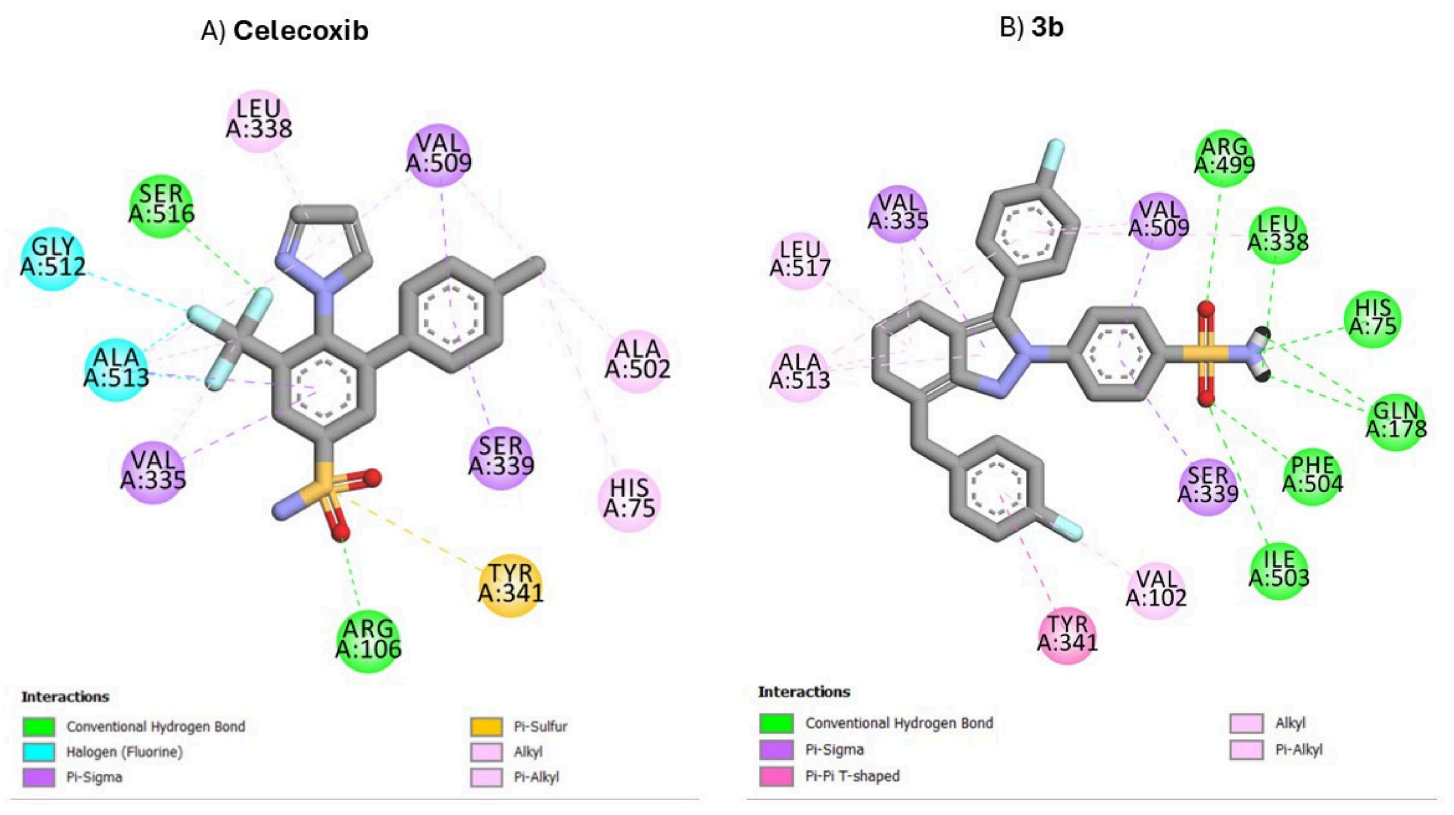
2D molecular docking snapshots illustrating the binding modes of (**A**) **Celecoxib** and (**B**) **compound 3b** within the COX-2 active site.

The molecular docking investigation provided compelling clarifications for the observed in vitro results. Compound **3b** exhibited a more potent action on the COX-2 isozyme compared to the reference medication celecoxib (IC_50_ = 0.079 µM versus IC_50_ = 0.108 µM). The elevated biological activity corresponds with the docking scores, indicating that **3b** exhibits superior binding affinity to celecoxib (−10.1, RMSD of 1.54) compared to celecoxib itself (−9.4, RMSD of 1.28). The structural cornerstone for this advantage appears to be the extensive network of hydrogen bonds established by the sulfonamide moiety of **3b**, which established six hydrogen bonds with the residues Arg499, Leu338, His75, Gln178, Phe504, and Ile503, Figure 4. Celecoxib, conversely, stabilized its binding by the formation of two hydrogen bonds (Arg106 and Ser616). Furthermore, both compounds exhibited analogous hydrophobic interactions with Val335 and Val509. Nonetheless, **3b** further stabilized itself by establishing a π–π T-shaped intersection with Tyr341. **3b**’s improved potency and selectivity profile is probably due to its ability to better occupy the COX-2 binding pocket, especially through multipoint binding with residues such as Arg499 and Phe504, which frequently reside in the secondary hydrophilic pocket of COX-2.

#### 2.3.2. Analysis of 3b and AUDA with sEH Active Site

To investigate the binding mechanism of the most potent dual-action compound, **3b**, we performed a docking analysis within the human sEH active site. Compound **3b** and the reference inhibitor AUDA were docked into the crystal structure of the human sEH catalytic domain (PDB ID: 1VJF) [36] to predict their likely interaction patterns.

**Figure 5:**
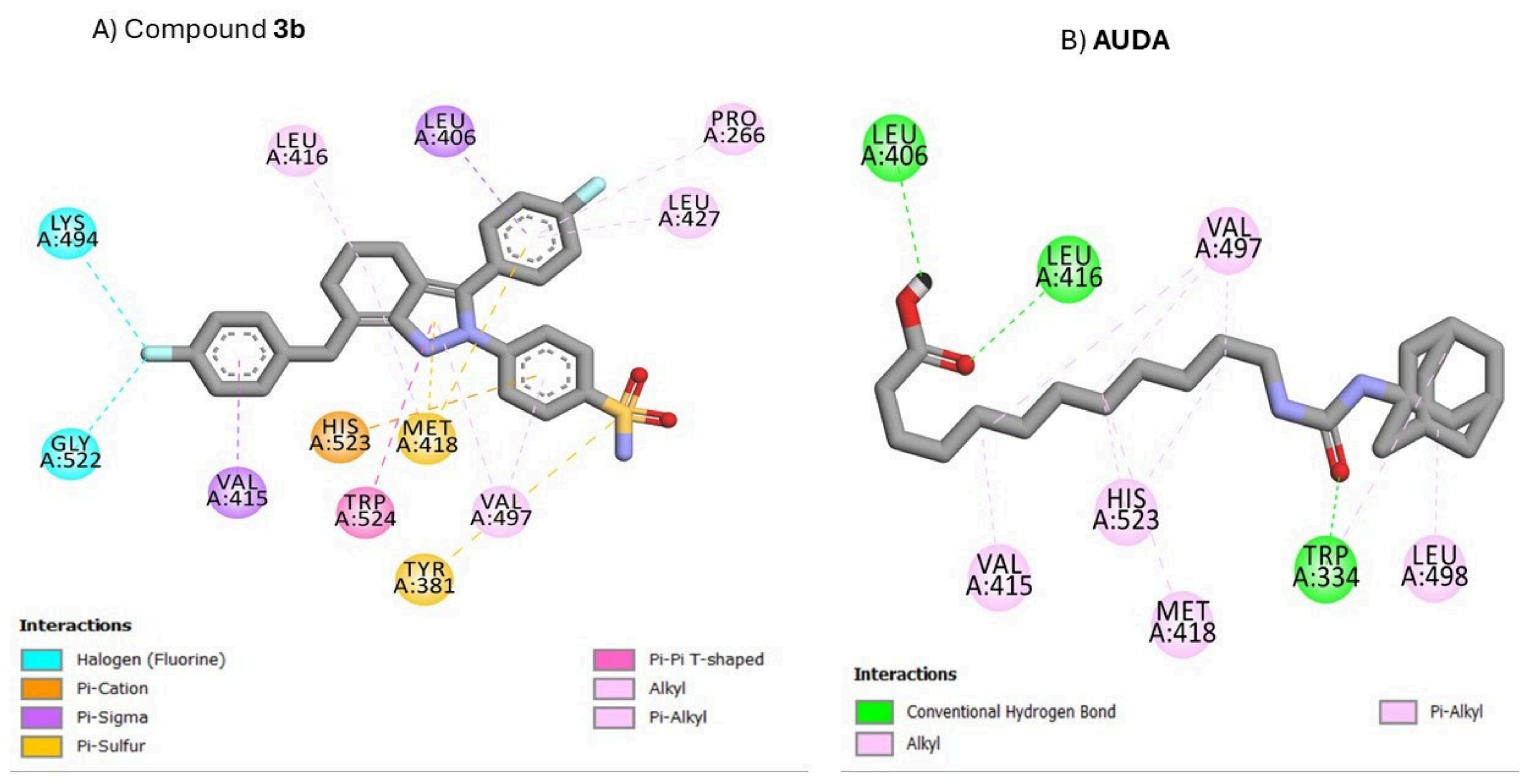
D molecular docking snapshots illustrating the binding modes of (**A**) **3b** and (**B**) **AUDA** within the sEH active site.

The comparative docking analysis of the reference ligand AUDA and compound **3b** reveals two apparent yet highly efficient mechanisms for inhibiting the sEH receptor. AUDA exhibits an IC_50_ of 8.32 nM and demonstrates greater potency due to its complex arrangement of “conventional hydrogen bonds” at the carboxylic acid and urea moieties. These bonds specifically engage residues Leu406, Leu416, and Trp334. This binding mode resembles the enzyme’s natural transition state, providing a robust anchor. Compound **3b** possesses a marginally reduced IC_50_ of 21.95 nM; however, it remains highly efficacious. The binding mechanism is intricate, involving both hydrophobic and electrical interactions. For instance, it possesses halogen (fluorine) connections with Lys494 and Gly522, Figure 5.

Due to their interactions with Val415, Met418, and Val497, both compounds have the same hydrophobic footprint. The potency discrepancy shows that AUDA’s direct hydrogen bonding provides a bigger energy advantage than **3b**’s aromatic stacking. The change from AUDA’s flexible alkyl chain to **3b**’s stiffer, multi-ring aromatic framework is a significant advance toward developing a molecule similar to a medicinal drug. **3b** loses the specific urea-hydrogen bond; however, it compensates by occupying the volume pocket and forming halogen bonds. This may increase its stability in the body and improve its pharmacokinetic profile in comparison to the reference compound.

### 2.4. Structure Activity Relationship (SAR) Analysis

The SAR of the developed hybrids (**3a**–**g** and **4a**–**e**) can be categorized based on the oxidation state of the indazole core and the electronic properties of the substituents on the peripheral phenyl rings.


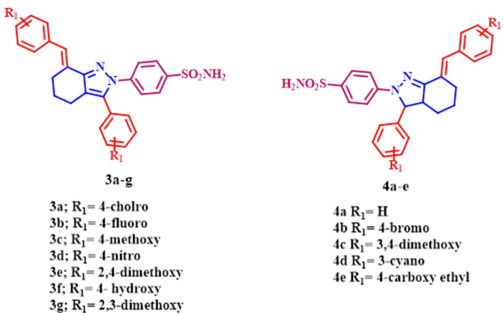

**1.**  **Impact of the Indazole Core (Tetrahydro vs. Hexahydro)**
  - The tetrahydroindazole series (**3a**–**g**) often has significantly greater potency against all three targets (COX-2, 5-LOX, and sEH) compared to the hexahydroindazole series (**4a**–**e**).
  - The planar, more rigid configuration of the 4,5,6,7-tetrahydro-2*H*-indazole core in series **3a**–**g** appears to facilitate a superior fit within the hydrophobic pockets of the enzymes compared to the more flexible hexahydro core in series **4a**–**e**.
**2.**  **Electronic Effects of Substituents (Series 3)**
  - **Halogenation:** The crucial factor for multi-target action is the presence of *para*-halogen atoms on the benzylidene and phenyl rings.
    ○ The 4-F derivative (**3b**) exhibited the highest potency against the three targets. The high electronegativity and small size of fluorine may enhance metabolic stability and binding via unique halogen bonding [37,38].
    ○ **Chlorine (3a):** Replacing Fluorine with the slightly bulkier **4-Cl** maintained strong activity, though it was slightly less potent than **3b**.
  - **Electron-Donating Groups (EDG),** such as methoxy (**3c**, **3e**, **3g**) or hydroxy (**3f**), significantly reduced COX-2 selectivity and sEH inhibition. The 4-OCH_3_ (**3c**) and dimethoxy (**3e**, **3g**) derivatives exhibited significantly elevated IC_50_ values. This indicates that large, oxygen-rich groups may impair the enzyme’s function by rendering its active sites less conducive to electron transfer [39].
  - **Electron-Withdrawing Groups (EWG):** Halogens served as effective EWGs; however, the highly polar 4-NO_2_ (**3d**) group inhibited sEH activity and significantly reduced COX-2/5-LOX efficacy, likely due to its excessive polarity or size [40].
**3.**  **COX-2 Selectivity and sEH Inhibition**
  - Compound **3b** had the greatest Selectivity Index (SI) at 82, nearly double that of Celecoxib, which recorded a SI of 42. The 4-F substitution sequence on the tetrahydroindazole scaffold aligns optimally with the COX-2 hydrophobic side pocket while avoiding the COX-1 narrow channel.
  - **sEH activity**: Only the halogenated (**3a**, **3b**) and hydroxylated (**3f**) compounds shown measurable efficacy against sEH. The transition from **3b** (F) to **3f** (OH) resulted in a 3-fold reduction in sEH potency, indicating that a hydrophobic, halogenated moiety is essential for sub-micromolar sEH suppression [40].
**4.**  **Developments in the Hexahydro Series (4a**–**e)**
  - This series had low selectivity (SIs ranging from >1 to 6).
  - The 3-CN (**4d**) and 3,4-dimethoxy (**4c**) derivatives exhibited moderate inhibition of COX-2 but were inferior to the tetrahydro series, indicating that saturating the indazole ring detrimentally affects the overall pharmacophore.

## 3. Materials and Methods

### 3.1. Chemistry

**General details**: See Supplementary S1 (Supplementary File)

Compounds **2a**–**l** were prepared, and their physical and spectral data were confirmed by matching with the reported ones.

#### 3.1.1. General Synthesis of 4-(7-Benzylidene Derivatives-3-phenyl derivatives-4,5,6,7-tetrahydro-2H-indazol-2-yl)benzenesulphonamide (**3a**–**g**)

To a stirred suspension of benzylidene cyclohexanone derivatives **2** (1 mmol) in 25 mL of absolute ethanol containing sodium acetate (0.08 g, 1 mmol) and three drops of glacial acetic acid, 4-sulfonamidophenylhydrazine (0.178 g, 1 mmol) was added. The reaction mixture was heated under reflux for 30–36 h. Upon completion (monitored by TLC), the solvent was evaporated under reduced pressure. The resulting residue was cooled, filtered, and dried. Recrystallization from absolute ethanol yielded the target compounds **3a**–**g**.

##### 4-(7-(4-Chlorobenzylidene)-3-(4-chlorophenyl)-4,5,6,7-tetrahydro-2H-indazol-2-yl)benzenesulfonamide (**3a**)

Yield: (51%, white crystals); mp: 243–245 °C; ^1^H NMR (600 MHz, DMSO-*d_6_*) *δ* ppm 7.94 (d, *J* = 8.5 Hz, 2H, Ar-H), 7.80 (d, *J* = 8.6 Hz, 2H, Ar-H), 7.77 (d, *J* = 8.5 Hz, 2H, Ar-H), 7.52 (d, *J* = 8.5 Hz, 2H, Ar-H), 7.49 (s, 2H, NH_2_), 7.38 (d, *J* = 8.5 Hz, 2H, Ar-H), 7.18 (d, *J* = 8.5 Hz, 2H, Ar-H), 6.09 (s, 1H, =CH), 2.87 (t, *J* = 6.2 Hz, 2H, –CH_2_), 2.73 (t, *J* = 6.5 Hz, 2H, –CH_2_), 1.87–1.82 (m, 2H, –CH_2_); ^13^C NMR (151 MHz, DMSO-*d_6_*) *δ* ppm 148.1, 143.9, 143.7, 139.7, 135.4, 133.1, 132.2, 132.2, 131.2, 129.4, 129.3, 128.9, 128.9, 127.4, 126.6, 125.2, 119.4, 27.3, 24.9, 22.5; LCMS: *m*/*z* Calcd.: 509.0732, found (M+H): 509.9595; ESI-HRMS: M+H: Calcd. (C_26_H_21_Cl_2_N_3_O_2_S): 510.0810, found: 510.0822.

##### 4-(7-(4-Fluorobenzylidene)-3-(4-fluorophenyl)-4,5,6,7-tetrahydro-2H-indazol-2-yl)benzenesulfonamide (**3b**)

Yield: (65%, yellow crystals); mp: 186–188 °C; ^1^H NMR (600 MHz, DMSO-*d_6_*) *δ* ppm 7.91 (d, *J* = 8.6 Hz, 2H, Ar-H), 7.79–7.73 (m, 4H, Ar-H), 7.45 (s, 2H, NH_2_), 7.27 (t, *J* = 8.9 Hz, 2H, Ar-H), 7.17 (dd, *J* = 8.6, 5.8 Hz, 2H, Ar-H), 7.13 (t, *J* = 8.8 Hz, 2H, Ar-H), 6.08 (s, 1H, =CH), 2.83 (t, *J* = 6.1 Hz, 2H, –CH_2_), 2.70 (t, *J* = 6.2 Hz, 2H, –CH_2_), 1.85–1.78 (m, –CH_2_); ^13^C NMR (151 MHz, DMSO-*d_6_*) *δ* ppm 148.4, 143.8, 143.7, 139.7, 132.9, 131.4, 131.4, 129.3, 129.2, 128.8, 127.4, 126.6, 125.3, 119.0, 116.2, 116.1, 115.9, 115.7, 27.2, 24.9, 22.4; LCMS: Calcd.: 477.1323, found (M+H): 478.0367; ESI-HRMS: M+H: Calcd. (C_26_H_21_F_2_N_3_O_2_S): 478.1395, found: 478.1379.

##### 4-(7-(4-Methoxybenzylidene)-3-(4-methoxyphenyl)-4,5,6,7-tetrahydro-2H-indazol-2-yl)benzenesulfonamide (**3c**)

Yield: (54%, white powder); mp: 179–181 °C; ^1^H NMR (600 MHz, DMSO-*d_6_*) *δ* ppm 7.92 (d, *J* = 8.7 Hz, 2H, Ar-H), 7.77 (d, *J* = 8.7 Hz, 2H, Ar-H), 7.67 (d, *J* = 8.9 Hz, 2H, Ar-H), 7.47 (s, 2H, NH_2_ exchangeable with D_2_O), 7.09 (d, *J* = 8.7 Hz, 2H, Ar-H), 7.02 (d, *J* = 8.9 Hz, 2H, Ar-H), 6.88 (d, *J* = 8.8 Hz, 2H, Ar-H), 6.07 (s, 1H, =CH), 3.79 (s, 3H, OCH_3_), 3.72 (s, 3H, OCH_3_), 2.84 (t, *J* = 6.2 Hz, 2H, –CH_2_), 2.74 (t, *J* = 6.2 Hz, 2H, –CH_2_), 1.86–1.80 (m, 2H, –CH_2_); ^13^C NMR (151 MHz, DMSO-*d_6_*) *δ* ppm 159.5, 158.8, 149.2, 143.5, 130.8, 128.6, 127.3, 126.5, 126.1, 125.9, 118.4, 114.6, 114.3, 55.7, 55.7, 27.4, 25.0, 22.5; LCMS: Calcd.: 501.1722, found(M+H): 502.0603; ESI-HRMS: M+H: Calcd. (C_28_H_27_N_3_O_4_S): 502.1795, found: 502.1777.

##### 4-(7-(4-Nitrobenzylidene)-3-(4-nitrophenyl)-4,5,6,7-tetrahydro-2H-indazol-2-yl)benzenesulfonamide (**3d**)

Yield: (64%, red powder); mp: 230–232 °C; ^1^H NMR (600 MHz, DMSO-*d_6_*) *δ* ppm 8.30 (d, *J* = 7.1 Hz, 2H, Ar-H), 8.14 (d, *J* = 7.1 Hz, 2H, Ar-H), 8.01 (d, *J* = 8.9 Hz, 2H, Ar-H), 7.94 (d, *J* = 6.9 Hz, 2H, Ar-H), 7.83 (d, *J* = 8.5 Hz, 2H, Ar-H), 7.49 (s, 2H, NH_2_), 7.40 (d, 2H, Ar-H), 6.17 (s, 1H, =CH), 2.94 (t, *J* = 6.2 Hz, 2H, –CH_2_), 2.76 (t, *J* = 6.2 Hz, 2H, –CH_2_), 1.86–1.78 (m, 2H, –CH_2_); ^13^C NMR (151 MHz, DMSO-*d_6_*) *δ* ppm 147.2, 146.4, 144.3, 143.4, 139.9, 139.6, 131.9, 130.6, 128.0, 127.6, 126.8, 124.9, 124.6, 124.1, 124.0, 120.9, 109.8, 27.4, 24.9, 22.5; LCMS: Calcd.: 531.1213, found (M+H): 532.0409; ESI-HRMS: M+H: Calcd. (C_26_H_21_N_5_O_6_S): 532.1285, found: 532.1273.

##### 4-(7-(2,4-Dimethoxybenzylidene)-3-(2,4-dimethoxyphenyl)-4,5,6,7-tetrahydro-2H-indazol-2-yl)benzenesulfonamide (**3e**)

Yield: (49%, brown powder); mp: 215−217 °C; ^1^H NMR (600 MHz, DMSO-*d_6_*) *δ* ppm 8.20–7.39 (m, 5H, Ar-H), 7.37–6.75 (m, 4H, Ar-H, NH_2_), 6.72–6.56 (m, 3H, Ar-H), 6.55–6.46 (m, 1H, =CH), 3.82 (s, 6H, OCH_3_), 3.80 (s, 6H, OCH_3_), 3.74 (t, *J* = 2.4 Hz, 2H, −CH_2_), 2.85–2.69 (m, 2H, −CH_2_), 1.83–1.55 (m, 2H, −CH_2_); ESI-HRMS: M+H: Calcd. (C_30_H_31_N_3_O_6_S): 562.2006, found: 562.2020.

##### 4-(7-(4-Hydroxybenzylidene)-3-(4-hydroxyphenyl)-4,5,6,7-tetrahydro-2H-indazol-2-yl)benzenesulfonamide (**3f**)

Yield: (53%, gray powder); mp: 198−200 °C; ^1^H NMR (600 MHz, DMSO-*d_6_*) *δ* ppm 9.94 (s, 2H, OH), 7.53 (s, 2H, NH_2_), 7.36 (dd, *J* = 41.0, 8.6 Hz, 4H, Ar-H, NH_2_), 7.22 (dd, *J* = 55.5, 8.5 Hz, 1H, Ar-H), 7.11–6.93 (m, 2H, Ar-H), 6.83 (d, *J* = 8.6 Hz, 4H, Ar-H), 6.81–6.61 (m, 2H, Ar-H, =CH), 2.84 (t, *J* = 6.2 Hz, 4H, −CH_2_), 1.74–1.64 (m, 2H, −CH_2_); ESI-HRMS: M+Na: Calcd. (C_26_H_23_N_3_O_4_S): 496.1301, found: 496.1350.

##### 4-(7-(2,3-Dimethoxybenzylidene)-3-(2,3-dimethoxyphenyl)-4,5,6,7-tetrahydro-2H-indazol-2-yl)benzenesulfonamide (**3g**)

Yield: (46%, brown powder); mp: 206−208 °C; ^1^H NMR (600 MHz, DMSO-*d_6_*) *δ* ppm 7.89–7.69 (m, 2H, Ar-H), 7.68–7.31 (m, 2H, Ar-H), 7.30–7.03 (m, 5H, Ar-H, NH_2_), 6.98 (t, *J* = 8.4 Hz, 4H, Ar-H, =CH), 3.80 (s, 6H, OCH_3_), 3.69 (s, 6H, OCH_3_), 3.08–2.53 (m, 4H, −CH_2_), 1.68–1.59 (m, 2H, −CH_2_); LCMS (M+H): Calcd. (C_30_H_31_N_3_O_6_S): 562.2006, found: 562.0803.

#### 3.1.2. General Synthesis of 4-(7-Benzylidene Derivatives-3-phenyl derivatives-3,3a,4,5,6,7-hexahydro-2H-indazol-2-yl)benzenesulfonamide (**4a**−**e**)

To a stirred suspension of benzylidene cyclohexanone derivatives **2** (1 mmol) in 20 mL of absolute ethanol containing three drops of piperidine, 4-sulfonamidophenylhydrazine (0.178 g, 1 mmol) was added. The mixture was heated under reflux for approximately 20 h, with the reaction progress monitored by TLC. Upon completion, the solvent was removed under reduced pressure. The resulting solid residue was cooled, collected by filtration, and dried. Recrystallization from absolute ethanol afforded compounds **4a**–**e**.

##### 4-(7-Benzylidene-3-phenyl-3,3a,4,5,6,7-hexahydro-2H-indazol-2-yl)benzenesulfonamide (**4a**)

Yield: (73%, green powder); mp: 218−220 °C; ^1^H NMR (600 MHz, DMSO-*d_6_*) *δ* ppm 7.55 (d, *J* = 9.0 Hz, 2H, Ar-H), 7.44–7.38 (m, 8H, Ar-H), 7.35–7.27 (m, 2H, Ar-H), 7.20 (d, 1H, =CH), 7.05 (s, 2H, NH_2_, exchangeable with D_2_O), 6.99 (d, *J* = 9.0 Hz, 2H, Ar-H), 4.87 (d, *J* = 11.4 Hz, 1H, -CH), 3.00 (m, 1H, -CH), 2.89 (d, *J* = 15.3 Hz, 1H, –CH_2_), 2.49–2.42 (m, 1H, –CH_2_), 2.12–2.05 (m, 1H, –CH_2_), 1.85 (d, *J* = 11.3 Hz, 1H, –CH_2_), 1.78–1.68 (m, 1H, –CH_2_), 1.48–1.37 (m, 1H, –CH_2_); ^13^C NMR (151 MHz, DMSO-*d_6_*) *δ* ppm 155.3, 148.3, 142.0, 136.5, 134.6, 131.1, 130.0, 129.8, 128.9, 128.2, 128.0, 127.3, 126.7, 126.3, 113.8, 71.8, 57.5, 29.1, 28.9, 24.1; LCMS: m/z Calcd. (C_26_H_25_N_3_O_2_S): 443.1667, found (M+H): 444.0609; ESI-HRMS: M+H: Calcd. (C_26_H_25_N_3_O_2_S): 444.1740, found: 444.1730.

##### 4-(7-(4-Bromobenzylidene)-3-(4-bromophenyl)-3,3a,4,5,6,7-hexahydro-2H-indazol-2-yl)benzenesulfonamide (**4b**)

Yield: (78%, green crystals); mp: 220−222 °C; ^1^H NMR (600 MHz, DMSO-*d_6_*) *δ* ppm 7.58 (dd, *J* = 16.4, 8.4 Hz, 6H, Ar-H), 7.36 (dd, *J* = 23.2, 8.5 Hz, 4H, Ar-H), 7.13 (d, *J* = 2.6 Hz, 1H, =CH), 7.06 (s, 2H, NH_2_, exchangeable with D_2_O), 6.97 (d, *J* = 9.0 Hz, 2H, Ar-H), 4.91 (d, *J* = 11.3 Hz, 1H, −CH), 3.03–2.96 (m, 1H, −CH), 2.83 (d, *J* = 15.3 Hz, 1H, −CH_2_), 2.48–2.40 (m, 1H, −CH_2_), 2.12–2.04 (m, 1H, −CH_2_), 1.85 (d, *J* = 15.5 Hz, 1H, –CH_2_), 1.73 (m, 1H, −CH_2_), 1.49–1.35 (m, 1H, −CH_2_); ^13^C NMR (151 MHz, DMSO-*d_6_*) *δ* ppm 155.2, 148.0, 141.4, 135.7, 134.8, 132.7, 132.0, 131.9, 131.9, 128.6, 127.4, 125.6, 121.3, 121.2, 113.8, 71.0, 57.2, 28.9, 28.8, 24.0; LCMS: *m*/*z* Calcd.: 599.9950, found (M+H): 601.8730; ESI-HRMS: M+H: Calcd. (C_26_H_23_Br_2_N_3_O_2_S): 599.9950, found: 599.9954.

##### 4-(7-(3,4-Dimethoxybenzylidene)-3-(3,4-dimethoxyphenyl)-3,3a,4,5,6,7-hexahydro-2H-indazol-2-yl)benzenesulfonamide (**4c**)

Yield: (74%, brown powder); mp: 211−213 °C; ^1^H NMR (600 MHz, DMSO-*d_6_*) *δ* ppm 7.55 (d, *J* = 9.3 Hz, 2H, Ar-H), 7.16 (d, *J* = 3.3 Hz, 3H, Ar-H), 7.04 (s, 2H, NH_2_), 7.03–7.00 (m, 2H, Ar-H), 7.00–6.92 (m, 3H, Ar-H), 6.92–6.88 (m, 1H, =CH), 4.73 (d, *J* = 11.8 Hz, 1H, −CH), 3.77 (s, 6H, OCH_3_), 3.74 (s, 6H, OCH_3_), 3.05–2.81 (m, 2H, −CH, −CH_2_), 2.09–2.06 (m, 1H, −CH_2_), 1.94–1.77 (m, 1H, −CH_2_), 1.75–1.58 (m, 1H, −CH_2_), 1.49–1.34 (m, 1H, −CH_2_), 1.04 (t, *J* = 7.1 Hz, 1H, −CH_2_); ^13^C NMR (151 MHz, DMSO-*d_6_*) *δ* ppm 155.6, 149.8, 148.9, 148.9, 148.8, 148.8, 134.4, 134.3, 129.3, 129.1, 127.2, 126.9, 122.8, 118.4, 113.9, 113.8, 112.8, 112.0, 109.7, 72.0, 57.4, 56.5, 56.0, 56.0, 55.9, 29.0, 28.8, 24.1, 19.1; LCMS: Calcd.: 563.2090, found (M+H): 564.0656; ESI-HRMS: M+Na: Calcd. (C_30_H_33_N_3_O_6_S): 586.1982, found: 586.1957.

##### 4-(7-(3-Cyanobenzylidene)-3-(3-cyanophenyl)-3,3a,4,5,6,7-hexahydro-2H-indazol-2-yl)benzenesulfonamide (**4d**)

Yield: (55%, white powder); mp: 209–211 °C; ^1^H NMR (600 MHz, DMSO-*d_6_*) *δ* ppm 8.17–7.90 (m, 3H, Ar-H), 7.89–7.67 (m, 4H, Ar-H), 7.66–7.35 (m, 5H, Ar-H, NH_2_), 7.27–7.00 (m, 2H, Ar-H,), 6.95 (d, *J* = 9.2 Hz, 1H, =CH), 5.00 (d, *J* = 11.6 Hz, 1H, −CH), 2.95–2.54 (m, 2H, −CH, −CH_2_), 2.28–1.95 (m, 1H, −CH_2_), 1.90–1.55 (m, 2H, −CH_2_), 1.49–1.32 (m, 1H, −CH_2_), 1.22–1.11 (m, 1H, −CH_2_); ^13^C NMR (151 MHz, DMSO-*d_6_*) *δ* ppm 168.3, 158.9, 147.9, 135.1, 133.2, 130.2, 127.5, 124.7, 119.1, 113.2, 112.1, 99.9, 70.8, 49.8, 28.1, 22.6; LCMS: Calcd.: 493.1572, found(M+H): 494.03939; ESI-HRMS: M+Na: Calcd. (C_28_H_23_N_5_O_2_S): 516.1465, found: 516.1493.

##### Ethyl-4-(7-(4-(ethoxycarbonyl)benzylidene)-2-(4-sulfamoylphenyl)-3,3a,4,5,6,7-hexahydro-2H-indazol-3-yl)benzoate (**4e**)

Yield: (81%, green crystals); mp: 223–225 °C; ^1^H NMR (600 MHz, DMSO-*d_6_*) *δ* ppm 7.97 (dd, *J* = 20.6, 8.6 Hz, 4H, Ar-H), 7.57 (dd, *J* = 9.0, 6.5 Hz, 4H, Ar-H), 7.53 (d, *J* = 8.6 Hz, 2H, Ar-H), 7.24 (d, *J* = 3.2 Hz, 1H, =CH), 7.07 (s, 2H, NH_2_), 6.97 (d, *J* = 9.6 Hz, 2H, Ar-H), 5.02 (d, *J* = 11.9 Hz, 1H, -CH), 4.30 (q, *J* = 7.7 Hz, 4H, -OCH_2_), 3.15–2.97 (m, 1H, –CH), 2.89 (d, *J* = 17.2 Hz, 1H, –CH_2_), 2.21–2.01 (m, 1H, –CH_2_), 1.87 (d, *J* = 16.5 Hz, 1H, –CH_2_), 1.82–1.63 (m, 1H, –CH_2_), 1.56–1.41 (m, 1H, –CH_2_), 1.38–1.22 (m, 7H, –CH_3_, –CH_2_); ^13^C NMR (151 MHz, DMSO-*d_6_*) *δ* ppm 165.9, 165.9, 155.0, 148.0, 147.2, 141.2, 135.0, 133.4, 130.7, 130.2, 129.9, 129.7, 128.9, 127.4, 126.7, 125.7, 113.9, 71.5, 61.3, 57.2, 29.0, 28.9, 24.0, 14.7; ESI-HRMS: M+Na: Calcd. (C_32_H_33_N_3_O_6_S): 610.1982, found: 610.1986.

### 3.2. Biology

#### 3.2.1. In Vitro COX-1/COX-2 Assays

The COX Colorimetric Inhibitor Screening Assay Kit (Cayman Chemical, Cat. 701050) was used to evaluate potential COX-1/COX-2 inhibitors [7]. The kit provides human recombinant COX-1 enzyme, COX-2 enzyme, assay buffer, hemin, arachidonic acid substrate, potassium hydroxide, colorimetric reagent (TMPD), 96-well plates, and covers. Kit components were stored and handled according to the manufacturer’s recommendations [41]. Supplementary S1 contains experimental details.

#### 3.2.2. 5-LOX Inhibitory Assay

The 5-Lipoxygenase Colorimetric Inhibitor Screening Assay Kit (Cayman Chemical, Cat. 760700) was employed to evaluate inhibitory potency against lipoxygenase (LO) enzymes [26]. The kit includes a purified soybean-derived 15-LO enzyme (usable as a proxy for 5-LO or other isoforms), 10× Assay Buffer, Developing Reagents 1 and 2, substrates (arachidonic and linoleic acids), potassium hydroxide solution, Zileuton as a positive control, a colorimetric 96-well plate, and covers [42]. Refer to Supplementary S1 for more experimental details.

#### 3.2.3. Soluble Epoxide Hydrolase (sEH) Assay

The inhibitory activity of compounds **3a**, **3b**, and **3f** against human recombinant soluble epoxide hydrolase (sEH) was evaluated using a fluorescence-based Soluble Epoxide Hydrolase Inhibitor Screening Assay Kit (Cayman Chemical, Ann Arbor, MI, USA; Item No. 10011671), according to the manufacturer’s protocol with slight optimization for dose–response analysis [6,43]. See Supplementary S1 for more details.

#### 3.2.4. Modulation of TNFα, LDH, and CK-MB

Compounds **3a**, **3b**, and **3f**, the most effective derivatives in all in vitro assays, were examined for modulatory impact against TNFα, LDH, and CK-MB. Lipopolysaccharide, a bacterial endotoxin derived from the outer membranes of Gram-negative bacteria, will be used to induce systemic inflammation. LPS is commonly used in experimental models to mimic acute inflammatory reactions by activating the innate immune system and stimulating the release of pro-inflammatory cytokines [44]. LPS was reconstituted in sterile physiological saline immediately before administration. Mice were administered a single intraperitoneal injection of LPS at a dosage of 5 mg/kg body weight, known to elicit a reliable systemic inflammatory response marked by elevated cytokine levels and signs of tissue damage [45]. See Supplementary S1 for experimental details.

## 4. Conclusions

In summary, this study presents a new class of tetrahydroindazole-sulfonamide hybrids, with compound **3b** emerging as a potent multi-target directed ligand (MTDL). Compound **3b** effectively inhibits COX-2 (IC_50_ = 0.08 µM, SI = 82), 5-LOX (IC_50_ = 0.46 µM), and sEH (21.95 nM) simultaneously. The synergistic action of the enzymes results in excellent cell defense. In LPS-challenged models, **3b** lowered TNF-α levels by 65.58%. Most notably, the molecule demonstrated remarkable cardioprotective activity, decreasing crucial markers of heart damage, LDH and CK-MB, by 76.26% and 76.76%, respectively. These findings, supported by molecular docking, identify **3b** as a superior lead candidate for modulating the arachidonic acid cascade while maintaining an outstanding cardiovascular safety profile.

**Future perspectives**

Future approaches will focus on optimizing the pharmacokinetic characteristics of the tetrahydroindazole-sulfonamide framework to provide sufficient oral bioavailability. Subsequent research will incorporate animal models of myocardial infarction and rheumatoid arthritis to validate the balanced inhibitory action of COX/5-LOX/sEH, demonstrating enhanced therapeutic efficiency with minimal gastrointestinal and renal adverse effects. These measures are essential to confirm compound **3b** as a viable therapeutic candidate for addressing complicated inflammatory and cardiovascular comorbidities.

## Figures and Tables

**Table 1:** IC_50_ values of compounds **3a**–**g** and **4a**–**e** against COX-1, COX-2, 5-LOX, and sEH.

Comp.	COX-1 (IC_50_ µM)	COX-2 (IC_50_ µM)	SI	5-LOX (IC_50_ µM)	sEH (IC_50_ nM)
**3a**	9.08 ± 0.41	0.27 ± 0.01	33	0.80 ± 0.03	27.56 ± 1.43
**3b**	6.54 ± 0.31	0.08 ± 0.004	82	0.46 ± 0.02	21.95 ± 0.78
**3c**	1.76 ± 0.01	1.37 ± 0.09	>1	0.87 ± 0.04	--
**3d**	7.18 ± 0.28	5.92 ± 0.22	>1	7.34 ± 0.44	--
**3e**	16.47 ± 0.86	4.17 ± 0.17	4	6.18 ± 0.37	--
**3f**	9.70 ± 0.50	0.89 ± 0.06	10	0.93 ± 0.07	66.29 ± 4.26
**3g**	0.84 ± 0.04	4.25 ± 0.33	--	1.10 ± 0.07	--
**4a**	4.97 ± 0.27	2.75 ± 0.12	2	6.47 ± 0.38	--
**4b**	3.74 ± 0.19	2.43 ± 0.17	>1	3.08 ± 0.14	--
**4c**	12.63 ± 0.59	2.14 ± 0.09	6	1.68 ± 0.06	--
**4d**	1.22 ± 0.07	1.09 ± 0.07	>1	5.91 ± 0.21	--
**4e**	11.29 ± 0.64	5.60 ± 0.29	2	2.87 ± 0.11	--
**Indomethacin**	1.11 ± 0.09	--	--	--	--
**Celecoxib**	4.58 ± 0.38	0.11 ± 0.01	42	--	39.15 ± 1.70
**Zileuton**	--	--	--	0.69 ± 0.03	--
**AUDA**	--	--	--	--	8.32 ± 0.39

--: Not Determined.

**Table 2:** Effect of compounds **3a**, **3b**, **3f**, and Celecoxib on Serum Levels of TNF-α, LDH, and CK-MB in LPS-induced inflammatory models.

Group	TNF-α (pg/mL)	TNF-α Inhibition (%) ^‡^	LDH (mIU/mL)	LDH Inhibition (%) ^‡^	CK-MB (ng/mL)	CK-MB Inhibition (%) ^‡^
**Normal Control**	20.85	—	88.73	—	1.10	—
**Positive Control (LPS)**	110.40	—	244.55	—	6.78	—
**3a**	59.36	57.00%	146.03	63.23%	4.25	44.54%
**3b**	51.67	65.58%	125.72	76.26%	2.42	76.76%
**3f**	69.04	46.19%	174.32	45.07%	6.08	12.32%
**Celecoxib**	41.75	76.66%	150.05	60.65%	3.25	62.15%

^‡^: Inhibition percentages represent the reduction of the LPS-induced increase relative to the normal control.

## Data Availability

The original contributions presented in this study are included in the article/Supplementary Material. Further inquiries can be directed to the corresponding authors.

## Supplementary Materials

The following supporting information can be downloaded at: https://www.mdpi.com/article/10.3390/ph19060843/s1.
