# Supplementary material for: Design, Synthesis, and Biological Evaluation of Tetrahydroindazole-Based Sulfonamides as Potential Multi-Target Anti-Inflammatory Agents
Source: Pharmaceuticals (Basel). 2026 May 28;19(6):843. doi: 10.3390/ph19060843 (PMC13305599; doi:10.3390/ph19060843)
Supplement: Supplementary file 1 [file pharmaceuticals-19-00843-s001.zip › pharmaceuticals-4324176-supplementary.pdf]

# Design, Synthesis, and Biological Evaluation of Tetrahydroindazole-Based Sulfonamides as Potential Multi-Target Anti-Inflammatory Agents

Mohammed A. I. Elbastawesy 1, Ahmed H. Abdelhafez 2, Abdullah Yahya Abdullah Alzahrani 3, Bandar A. Alyami 4, Hanyu Ling 5, Mahmoud S. Abdelbaset 1, Ahmed A. Gaber 6, Bahaa G. M. Youssif 7, Stefan Brase 8,* and Hiroyuki Konno 5,*

## Supplementary Data

### 1- Compound 3a

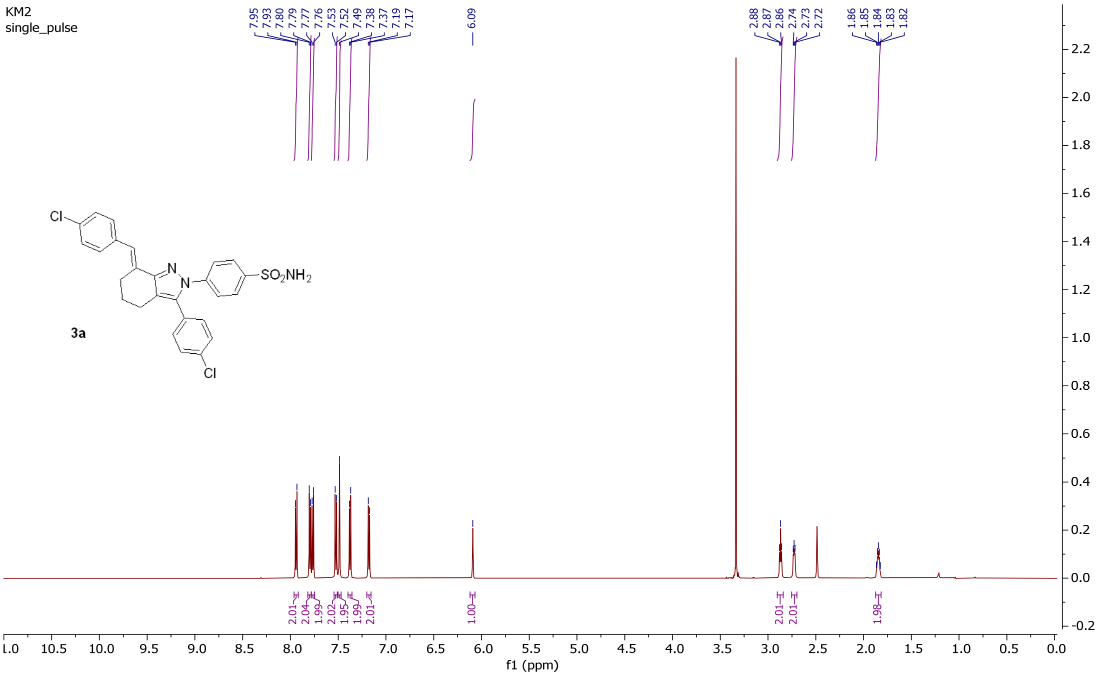


**Supplementary Figure S1. $^{1}$H NMR spectrum of compound 3a**

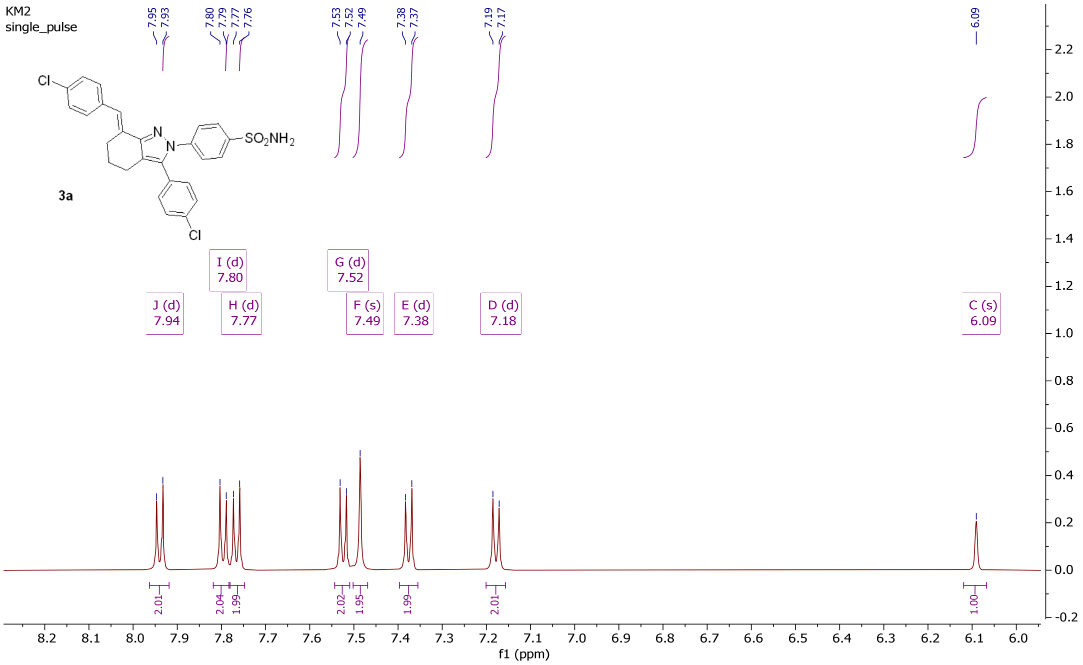


## Supplementary Figure S2. Expanded $^{1}$H NMR spectrum of compound 3a

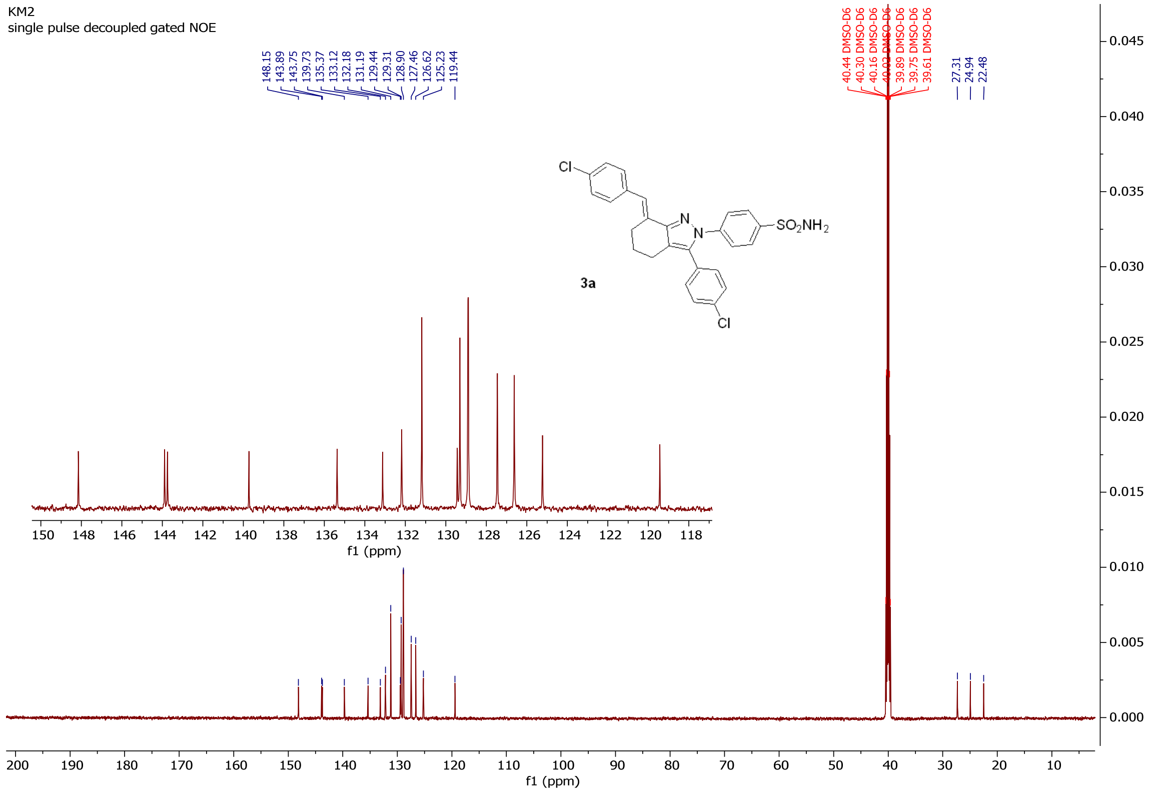


## Supplementary Figure S3. $^{13}$C NMR spectrum of compound 3a

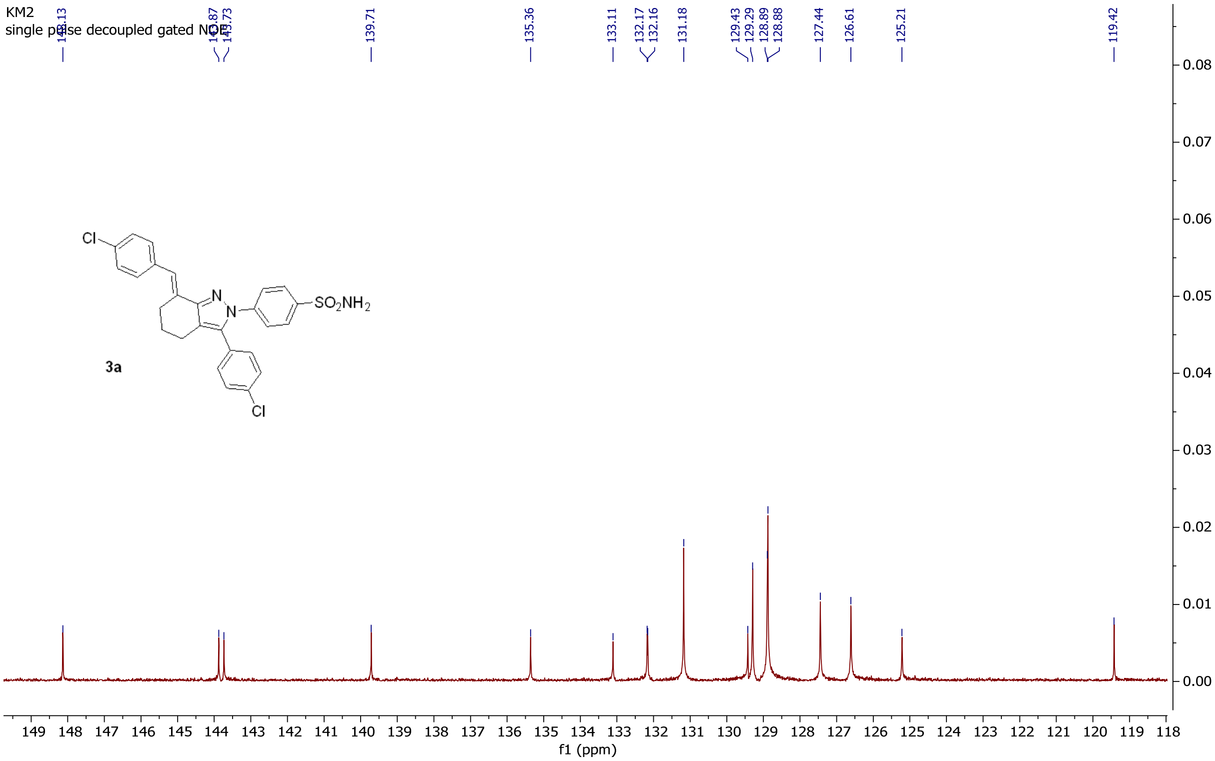


**Supplementary Figure S4. Expanded $^{13}C$ NMR spectrum of compound 3a**

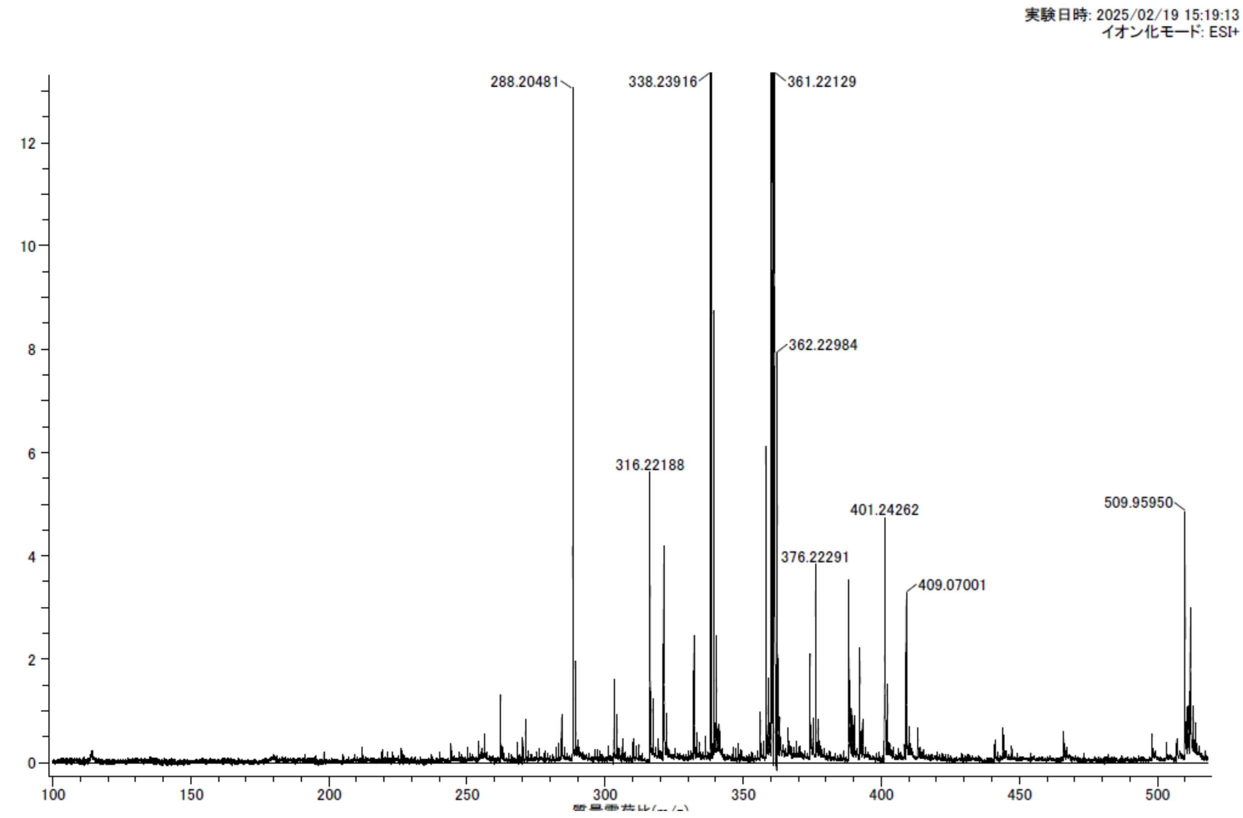

Supplementary Figure S5. LCMS spectrum of compound 3a

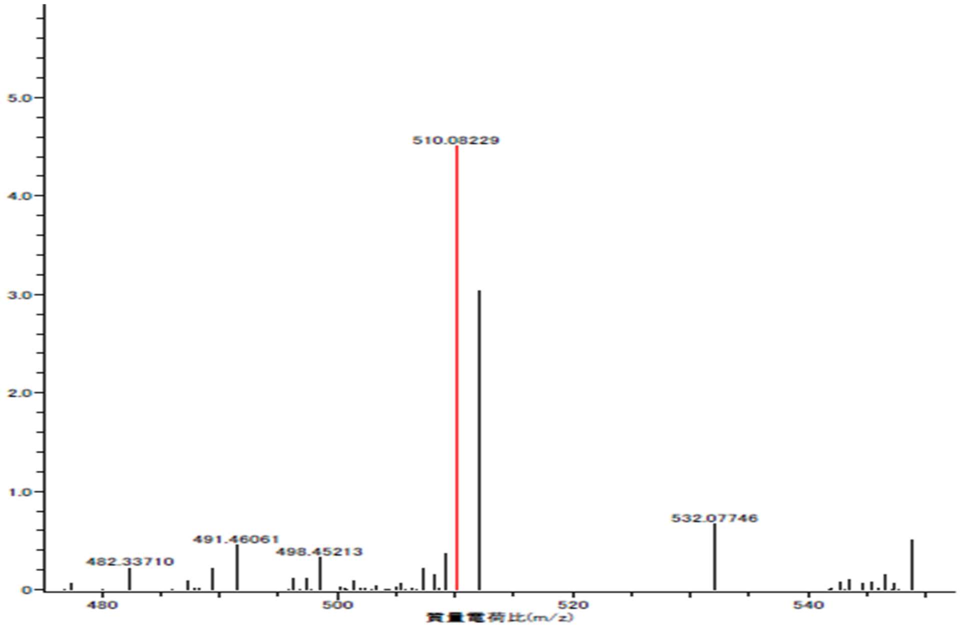


Supplementary Figure S6. ESI-HRMS spectrum of compound 3a

## 2- Compound 3b

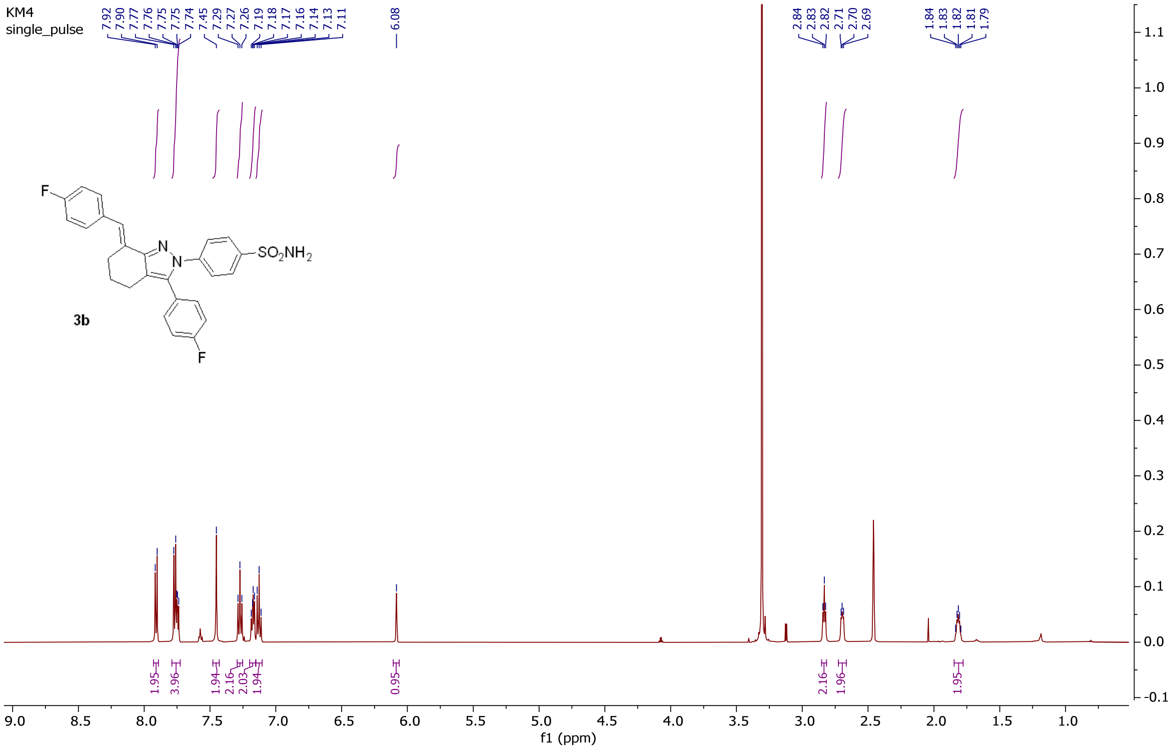


## Supplementary Figure S7. $^1$H NMR spectrum of compound 3b

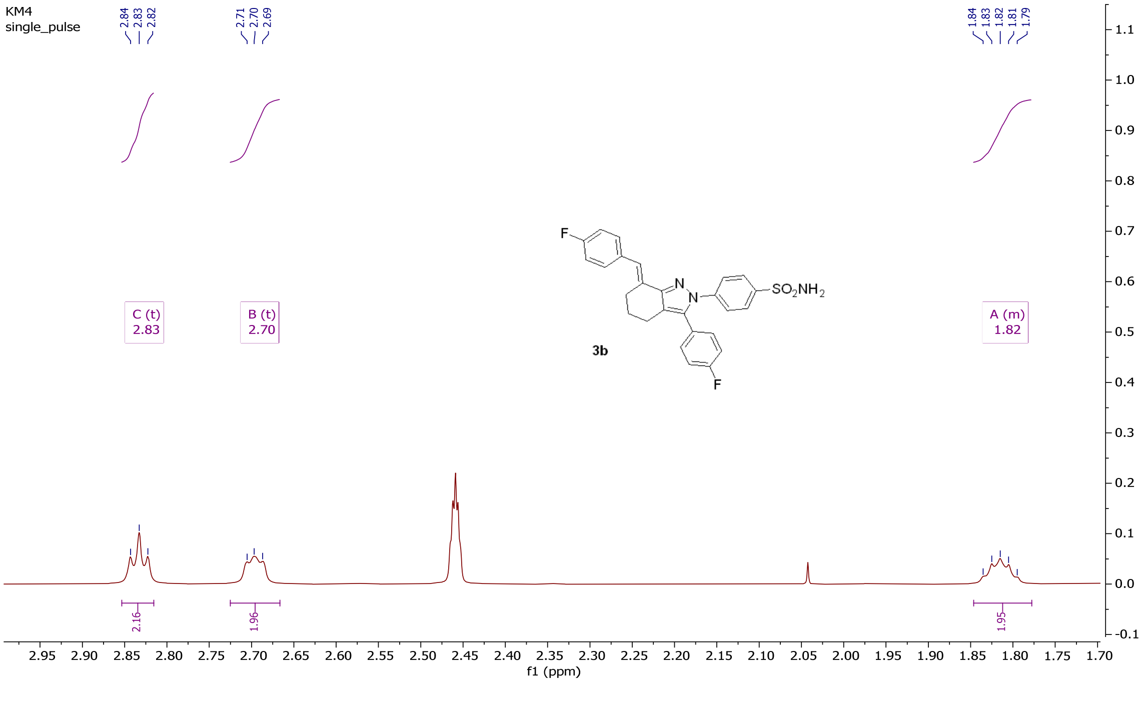


## Supplementary Figure S8. Expanded $^1$H NMR spectrum of compound 3b

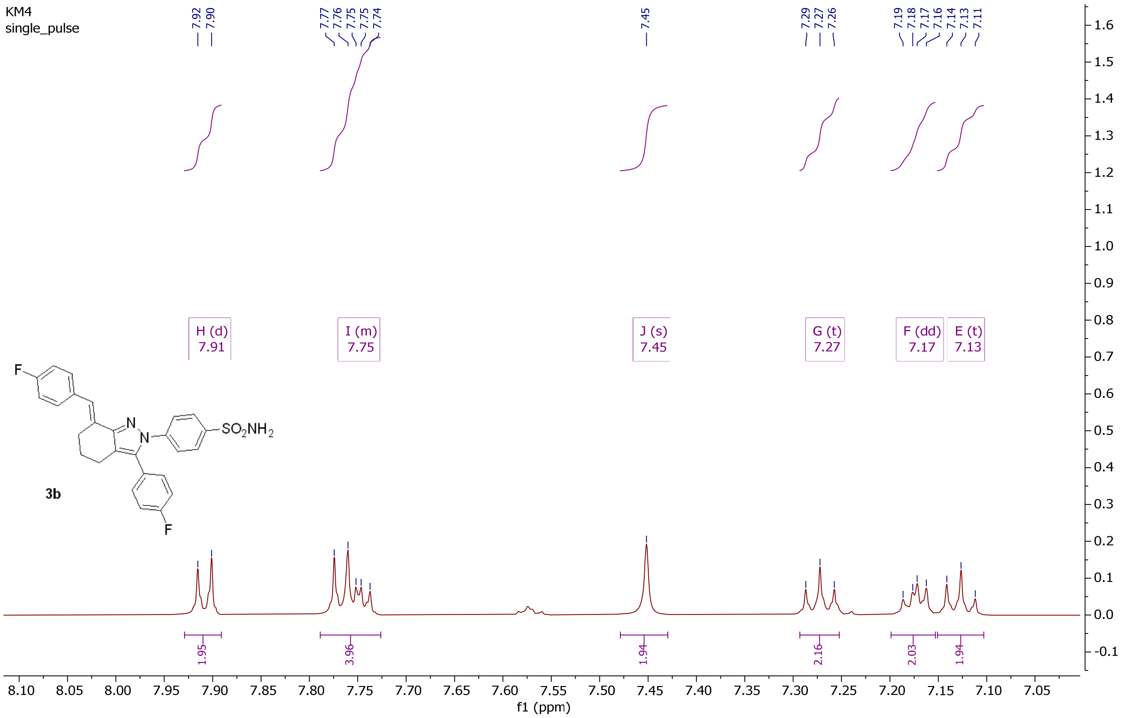

Supplementary Figure S9. Expanded $^1H$ NMR spectrum of compound 3b

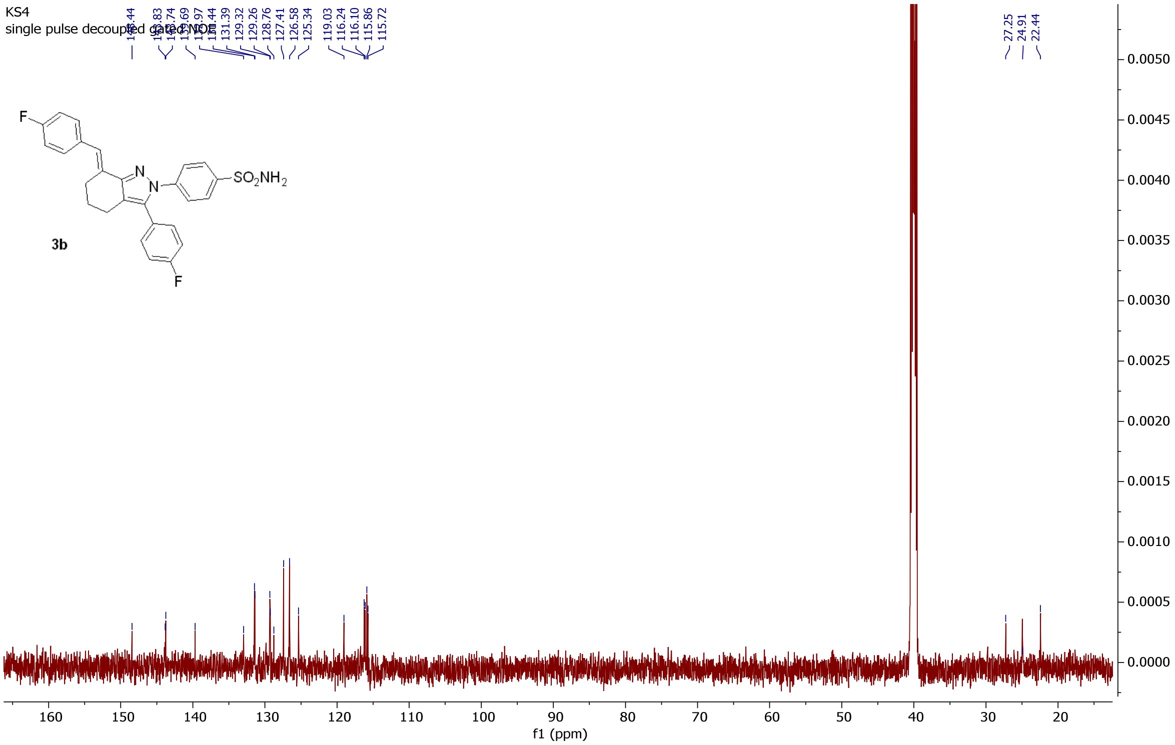

Supplementary Figure S10. $^{13}C$ NMR spectrum of compound 3b

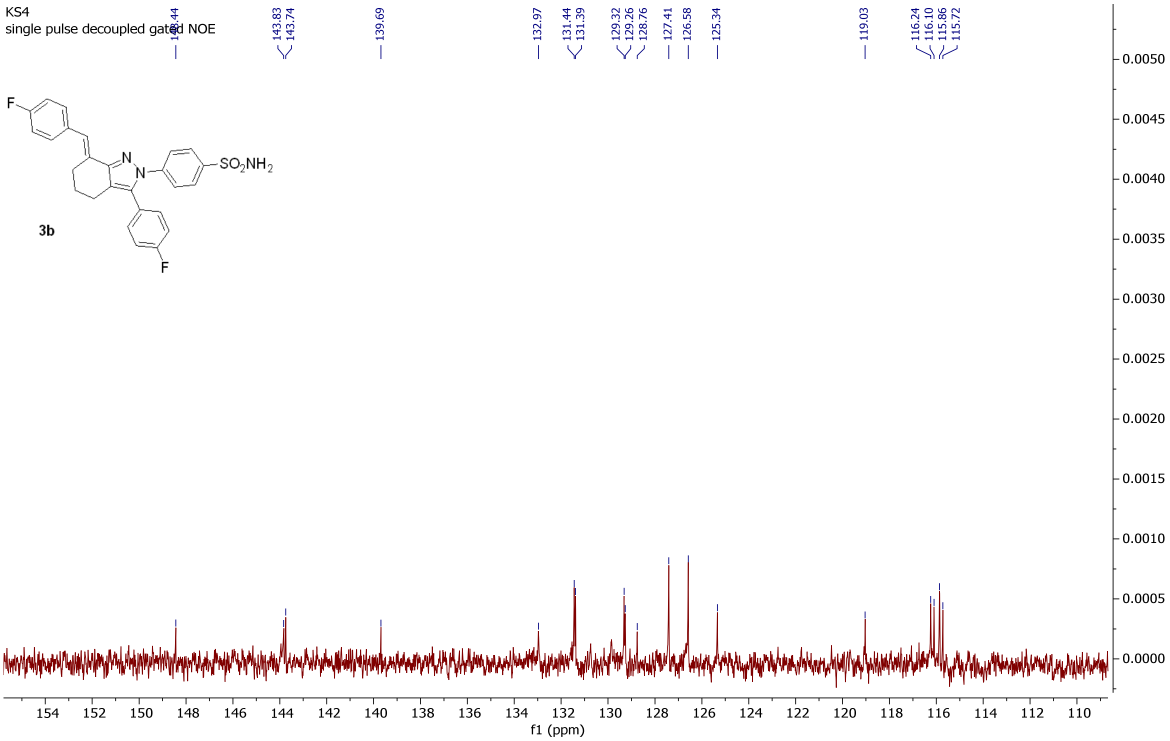


## Supplementary Figure S11. Expanded $^{13}C$ NMR spectrum of compound 3b

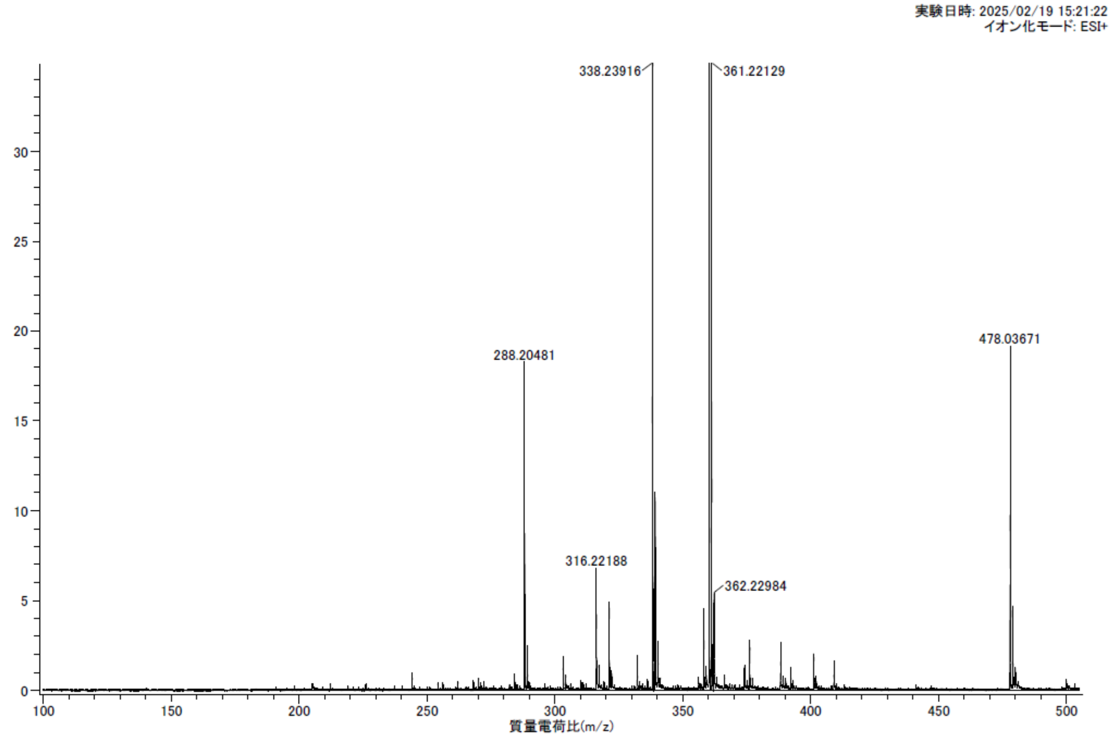


## Supplementary Figure S12. LCMS spectrum of compound 3b

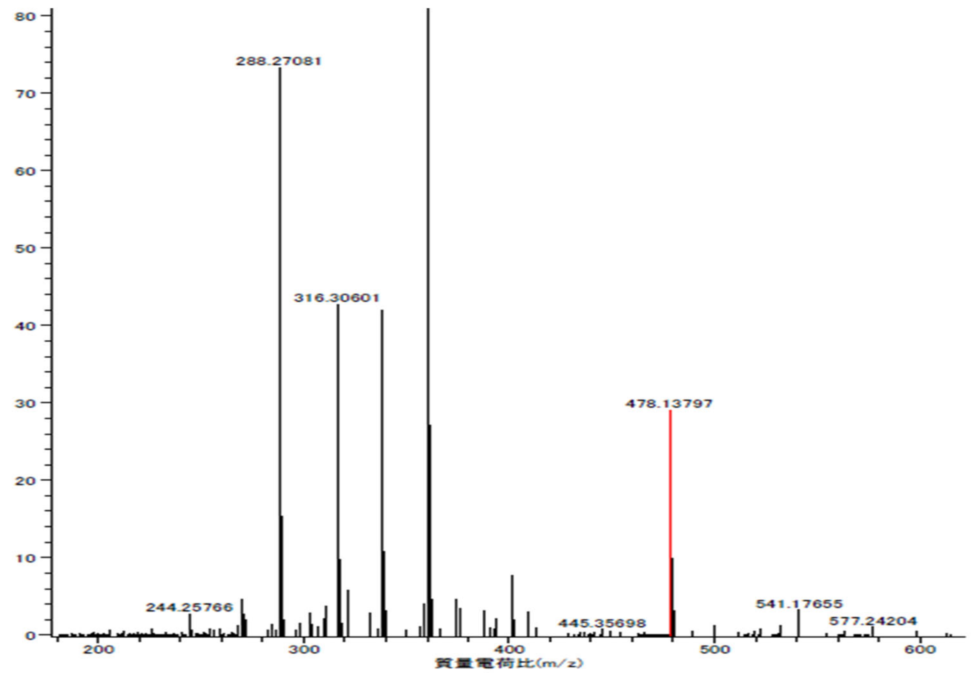


**Supplementary Figure S13. ESI-HRMS spectrum of compound 3b**

## 3- Compound 3c

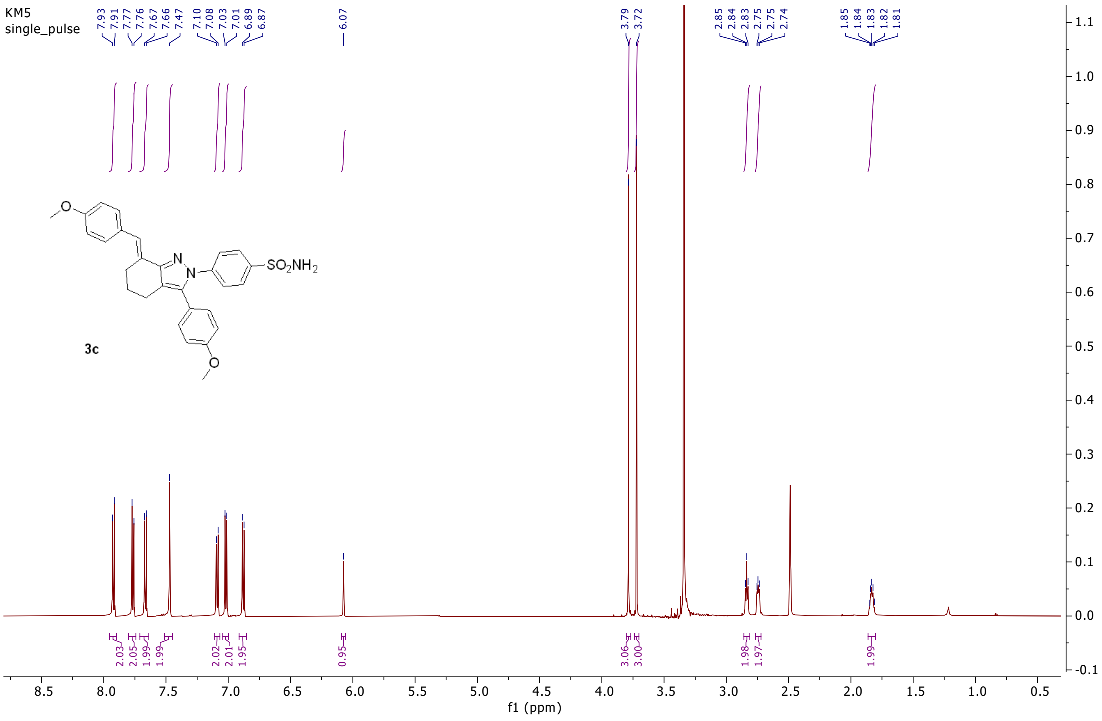


**Supplementary Figure S14. $^{1}$H NMR spectrum of compound 3c**

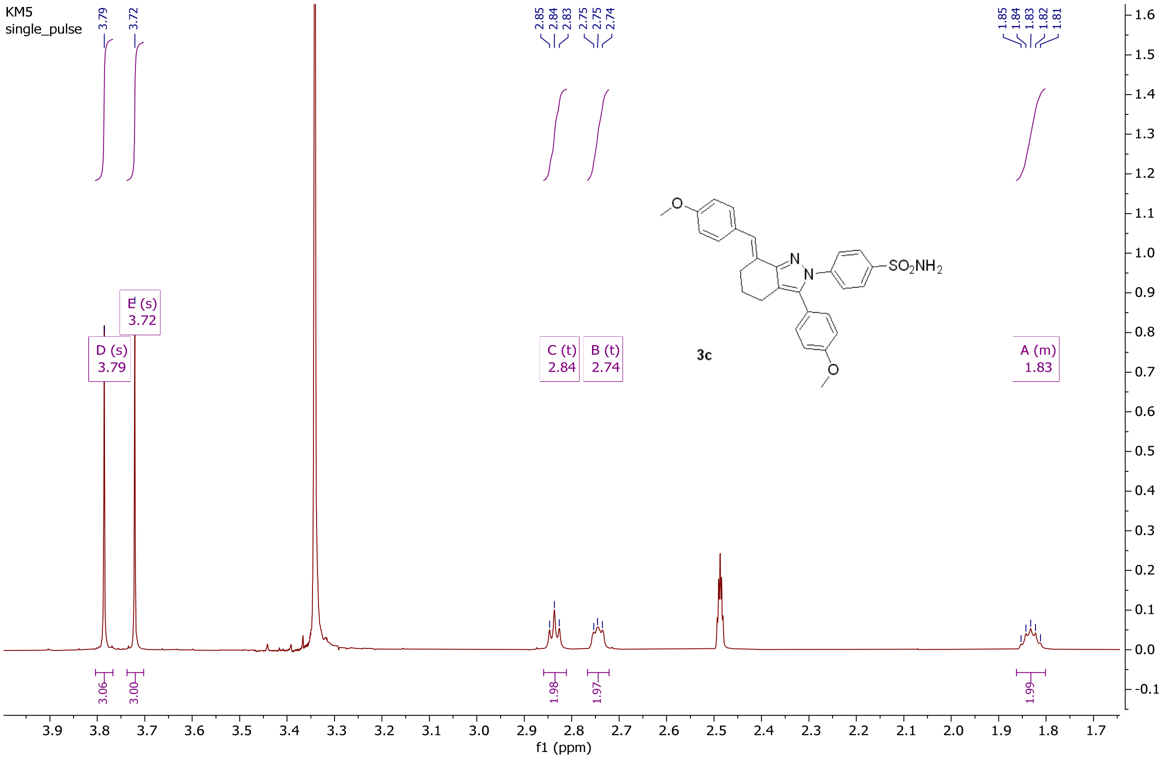


**Supplementary Figure S15. Expanded $^1$H NMR spectrum of compound 3c**

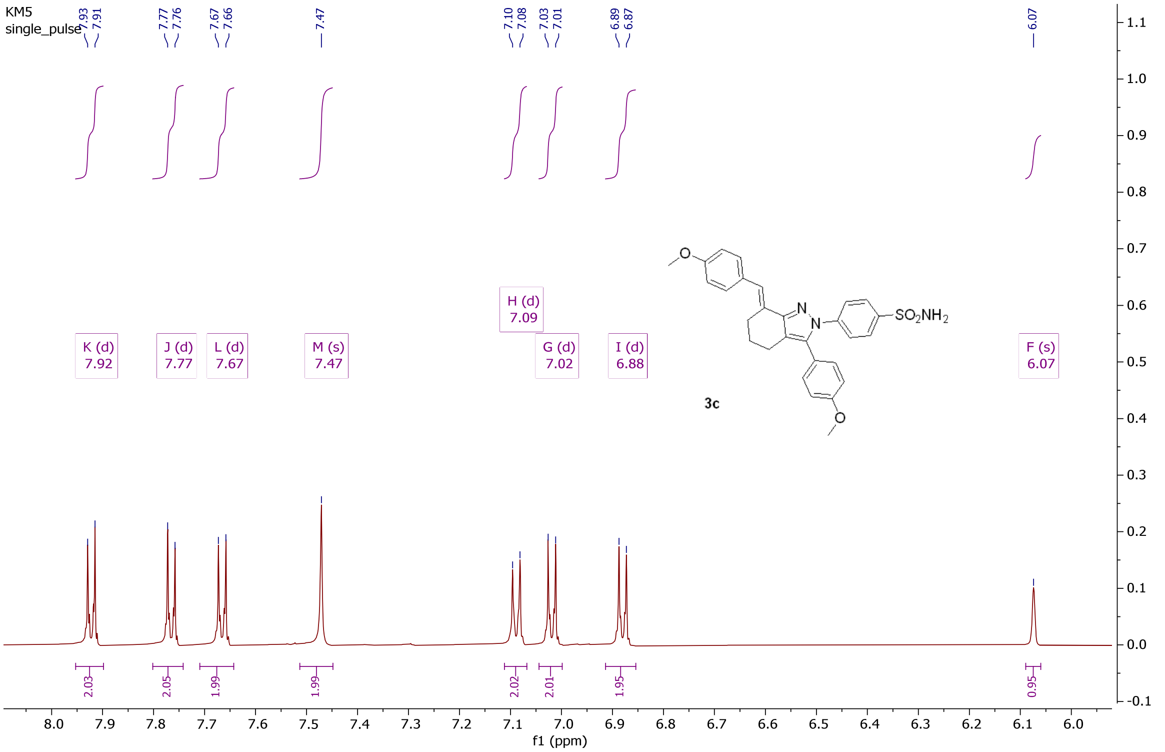


**Supplementary Figure S16. Expanded $^1$H NMR spectrum of compound 3c**

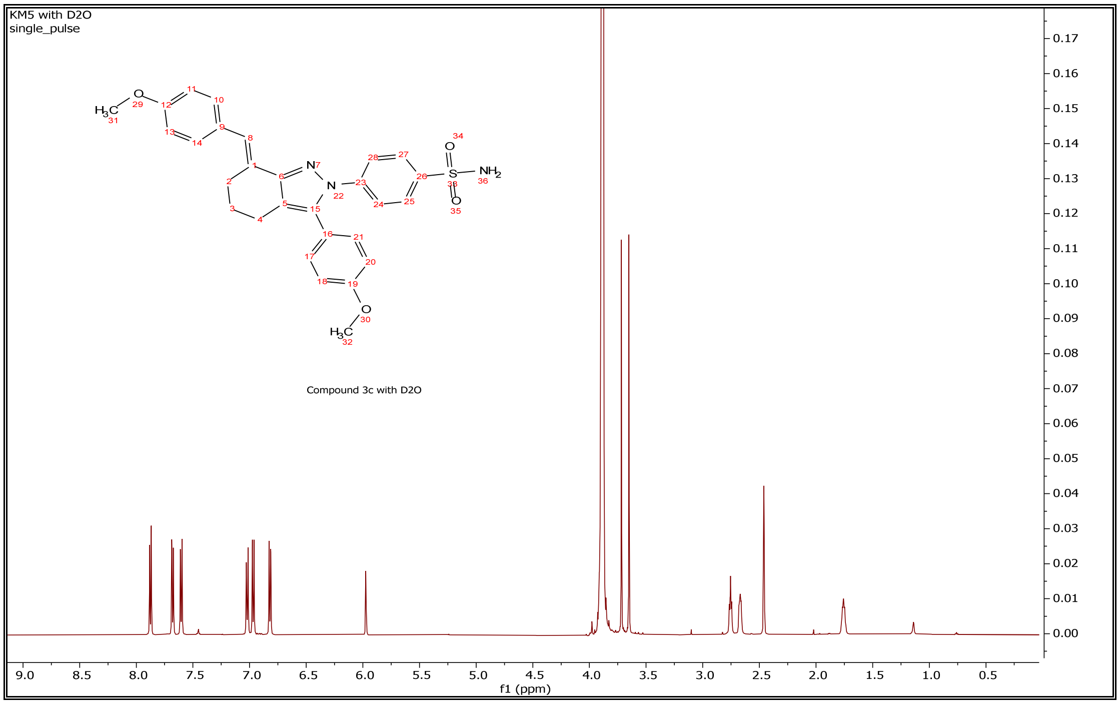


**Supplementary Figure S17. $^{1}$H NMR spectrum of compound 3c with $D_2O$**

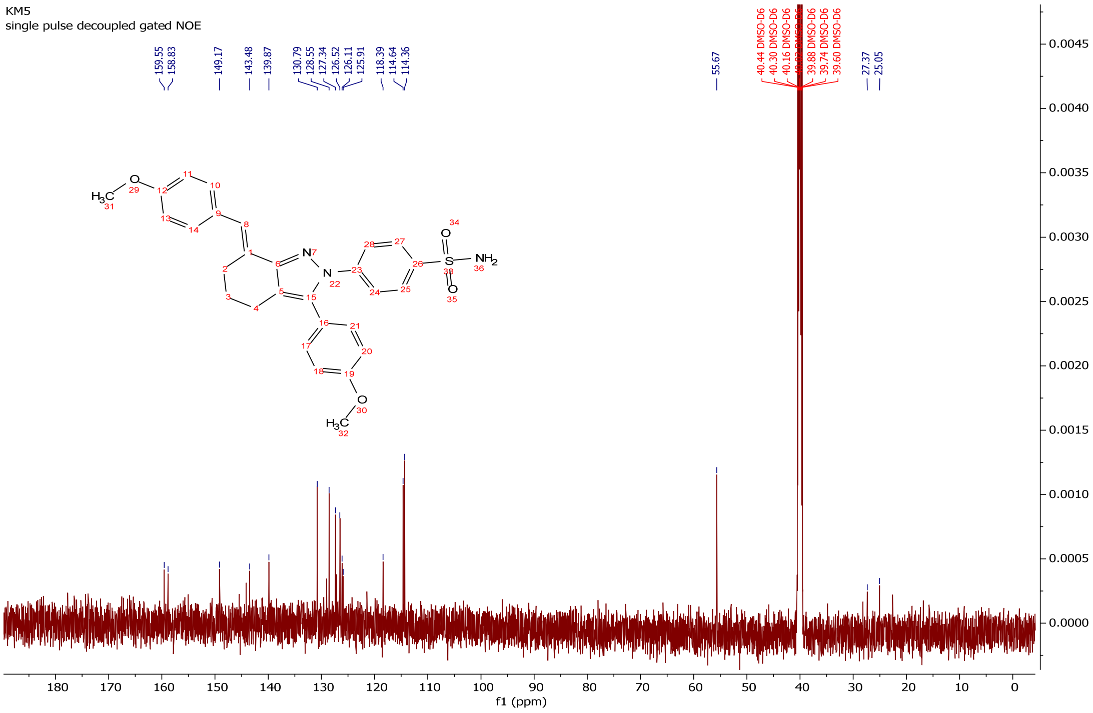


**Supplementary Figure S18. $^{13}$C NMR spectrum of compound 3c**

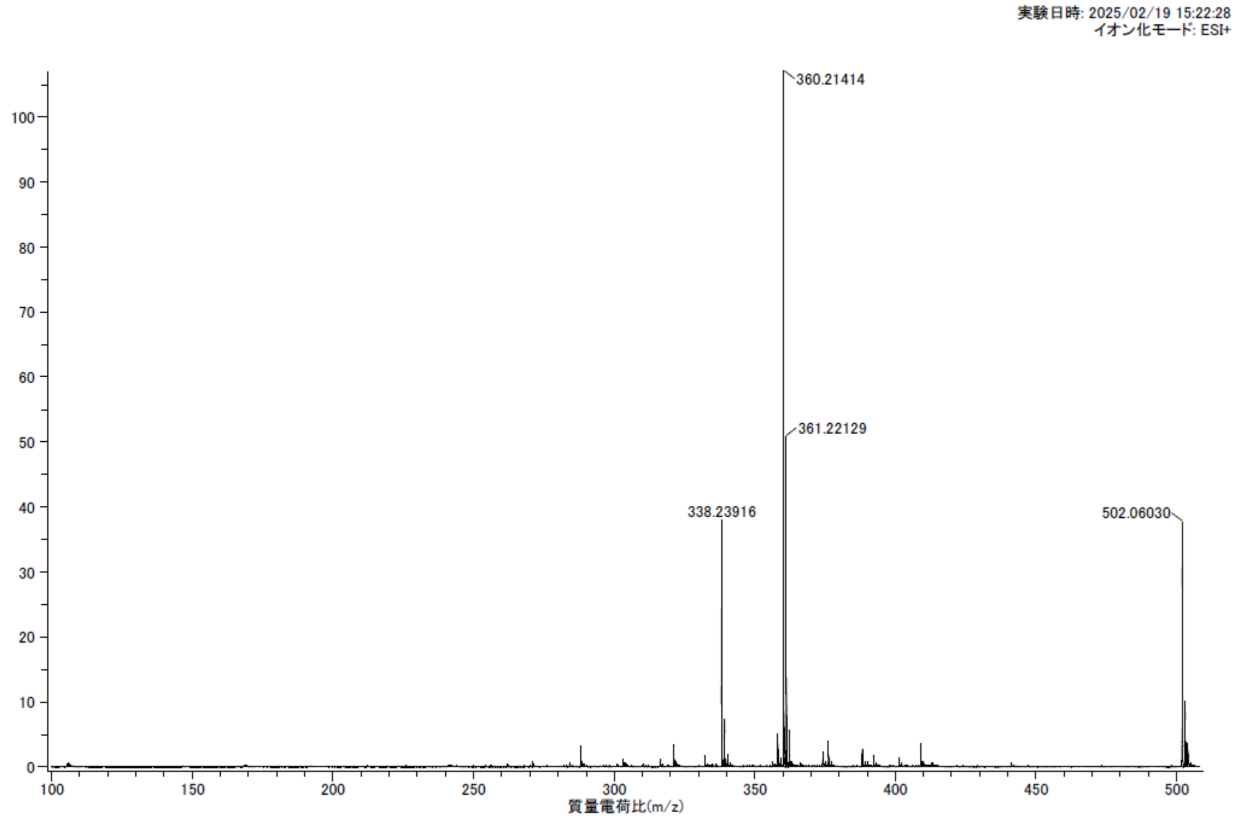


**Supplementary Figure S19. LCMS spectrum of compound 3c**

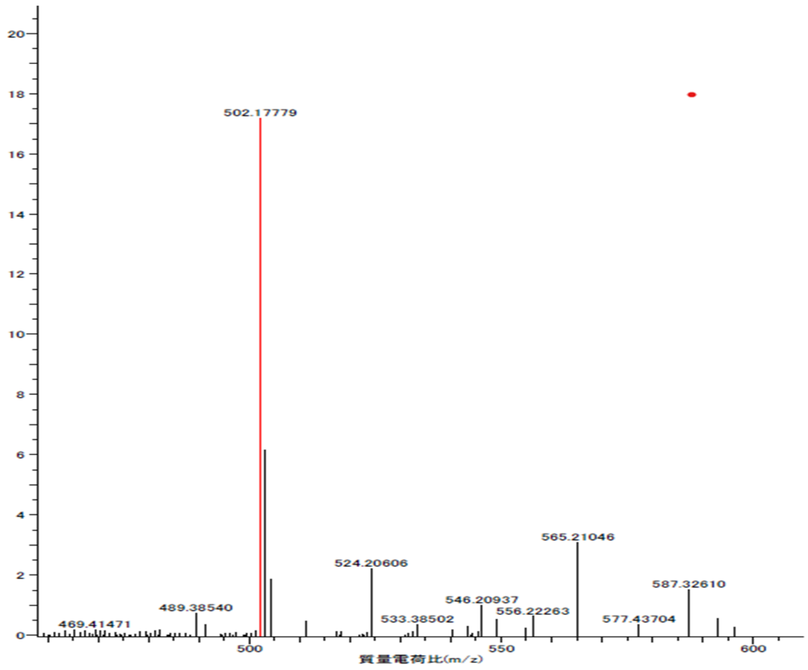


**Supplementary Figure S20. ESI-HRMS spectrum of compound 3c**

## 4- Compound 3d

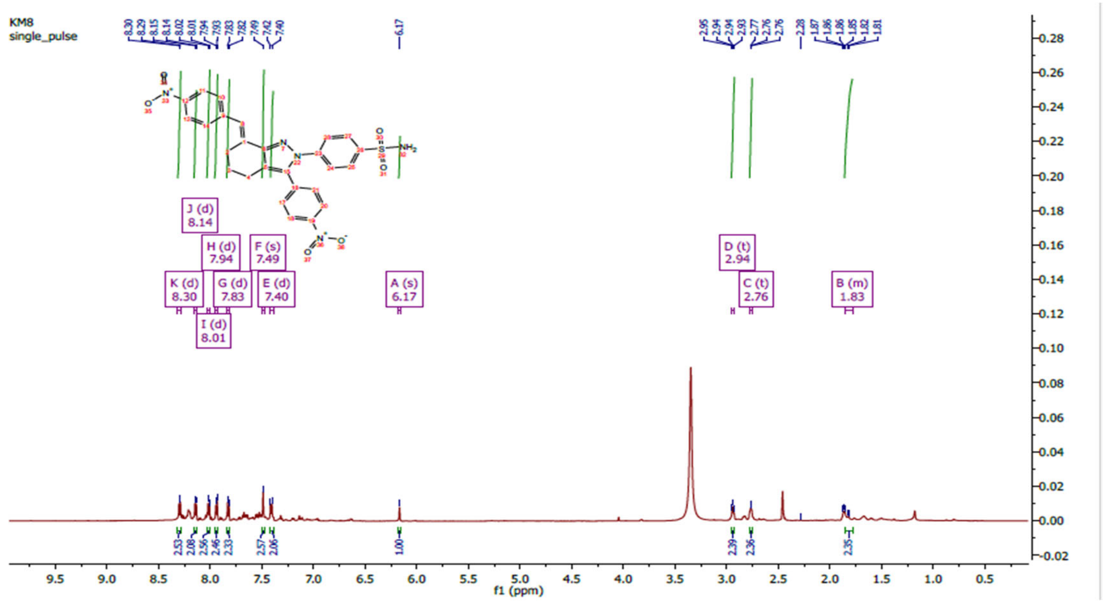

Supplementary Figure S21. $^{1}$H NMR spectrum of compound 3d

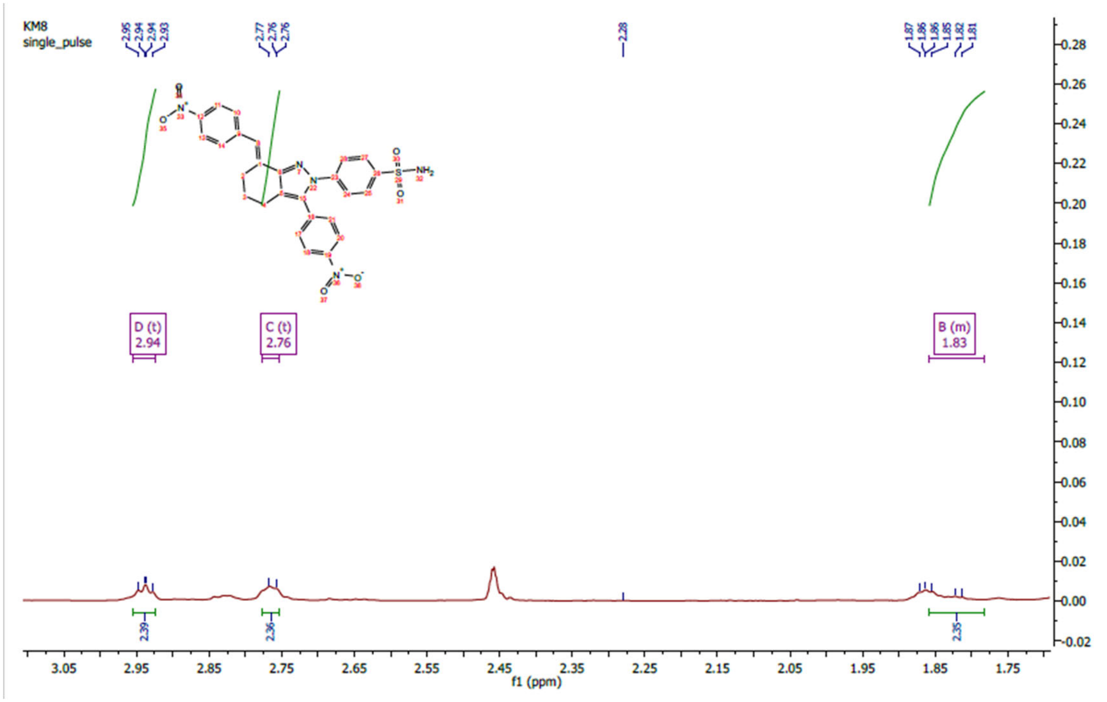

Supplementary Figure S22. Expanded $^{1}$H NMR spectrum of compound 3d

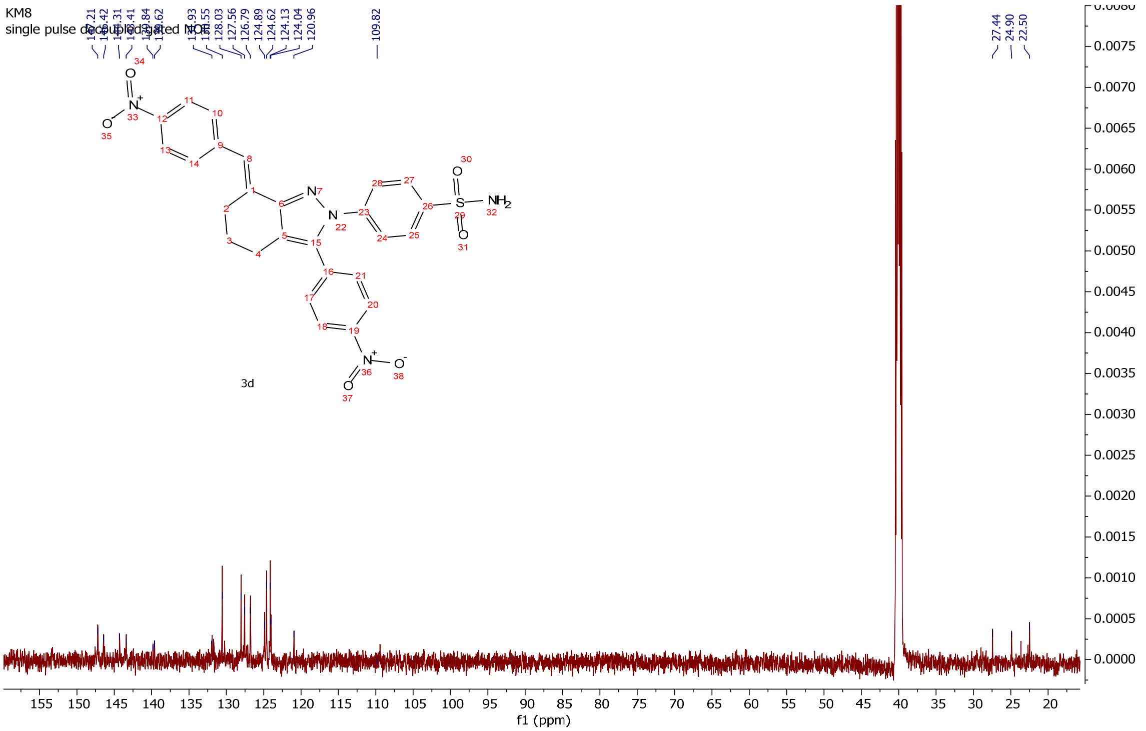


## Supplementary Figure S23. $^{13}C$ NMR spectrum of compound 3d

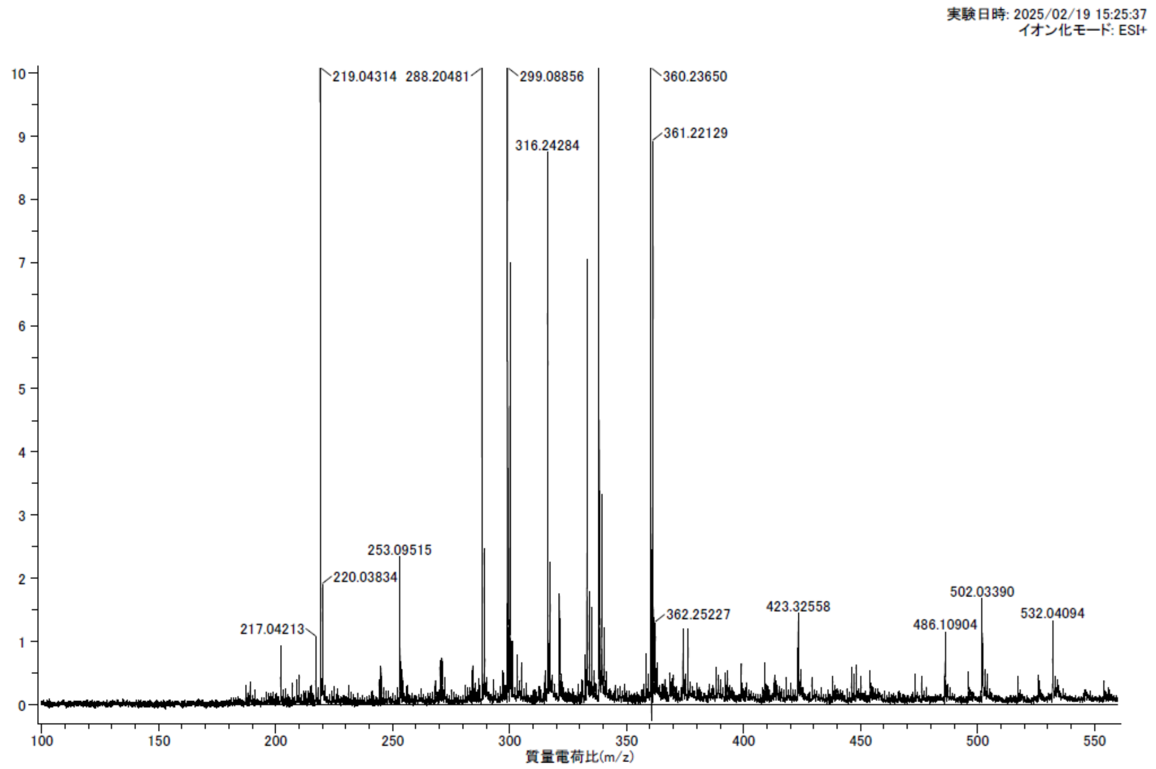


## Supplementary Figure S24. LCMS spectrum of compound 3d

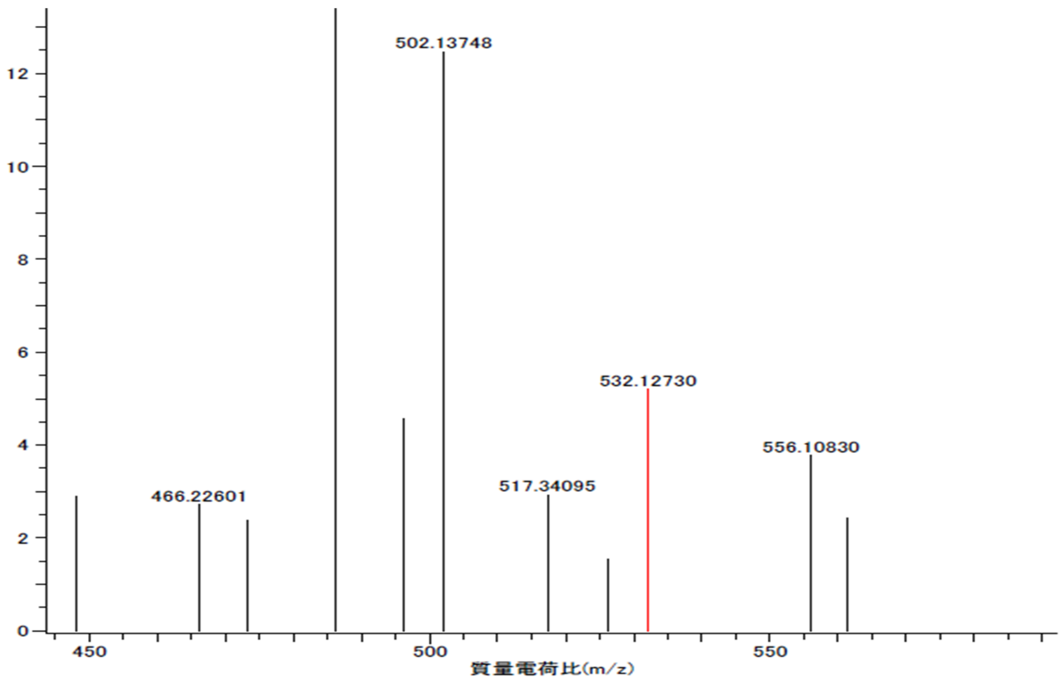


**Supplementary Figure S25. ESI-HRMS spectrum of compound 3d**

## 5- Compound 3e

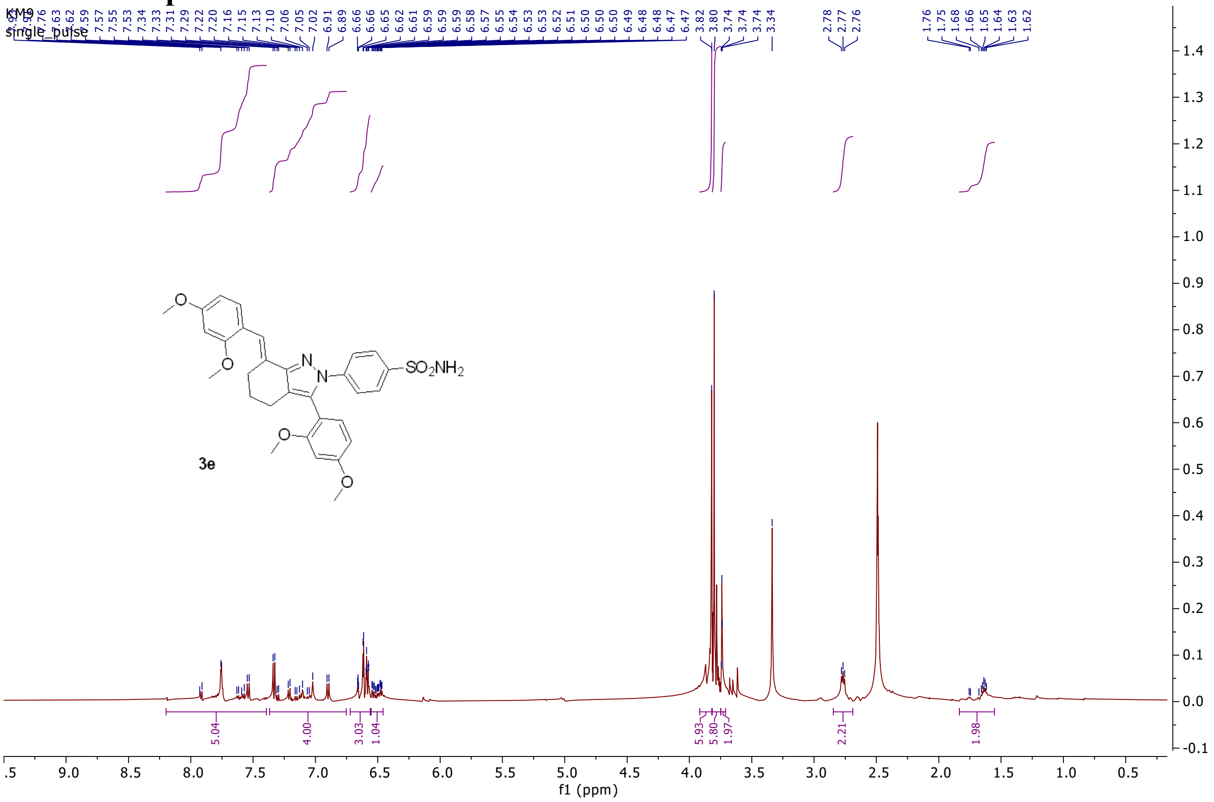


**Supplementary Figure S26. $^1$H NMR spectrum of compound 3e**

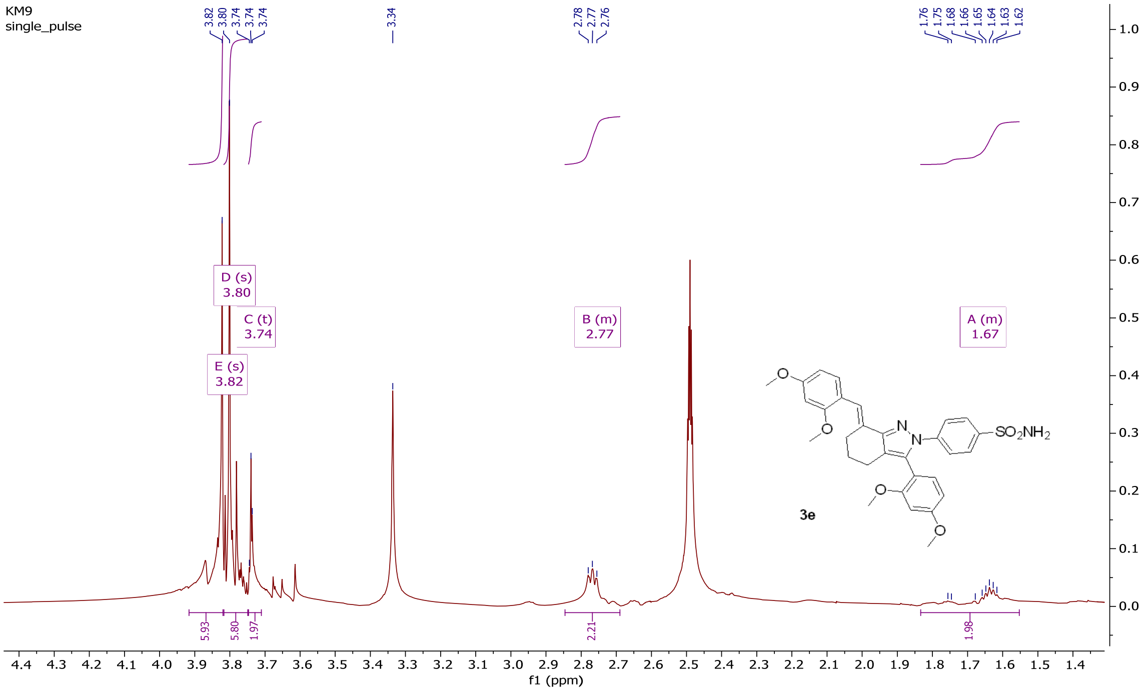


# Supplementary Figure S27. Expanded $^{1}H$ NMR spectrum of compound 3e

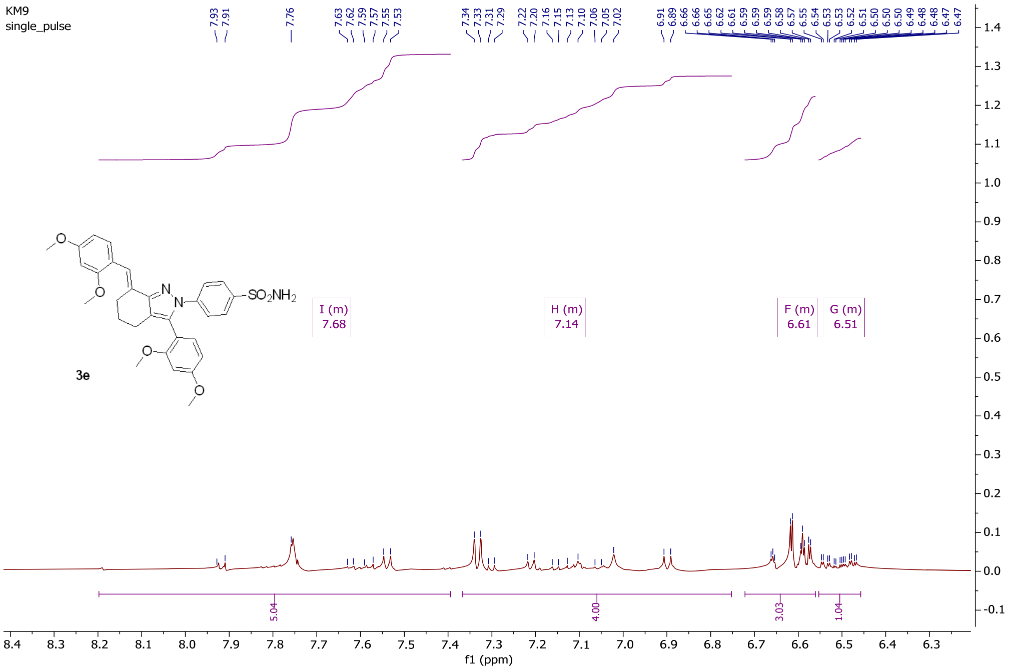


$^{1}H$ NMR (600 MHz, )δ 8.20 – 7.39 (m, 5H), 7.37 – 6.75 (m, 4H), 6.72 – 6.56 (m, 3H), 6.55 – 6.46 (m, 1H).

# Supplementary Figure S28. Expanded $^{1}H$ NMR spectrum of compound 3e

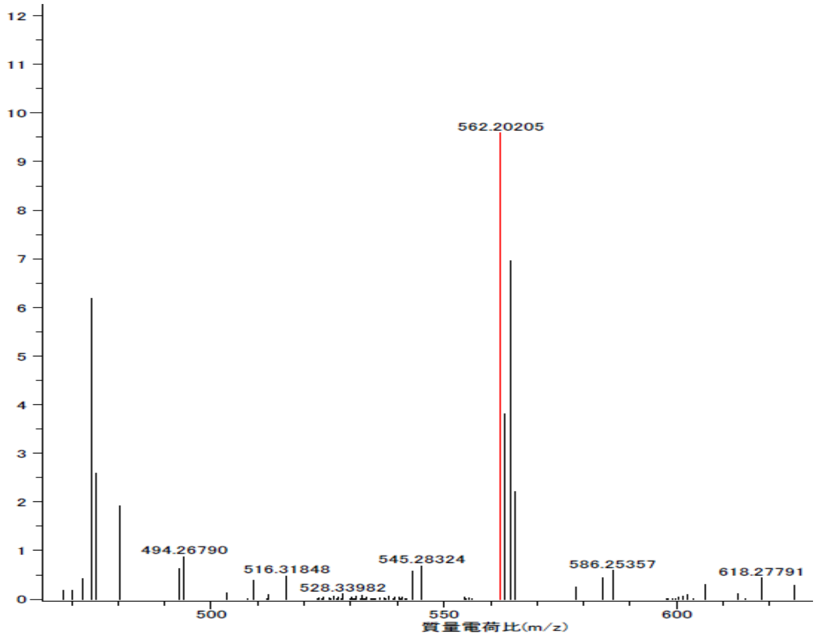


**Supplementary Figure S29. ESI-HRMS spectrum of compound 3e**

## 6- Compound 3f

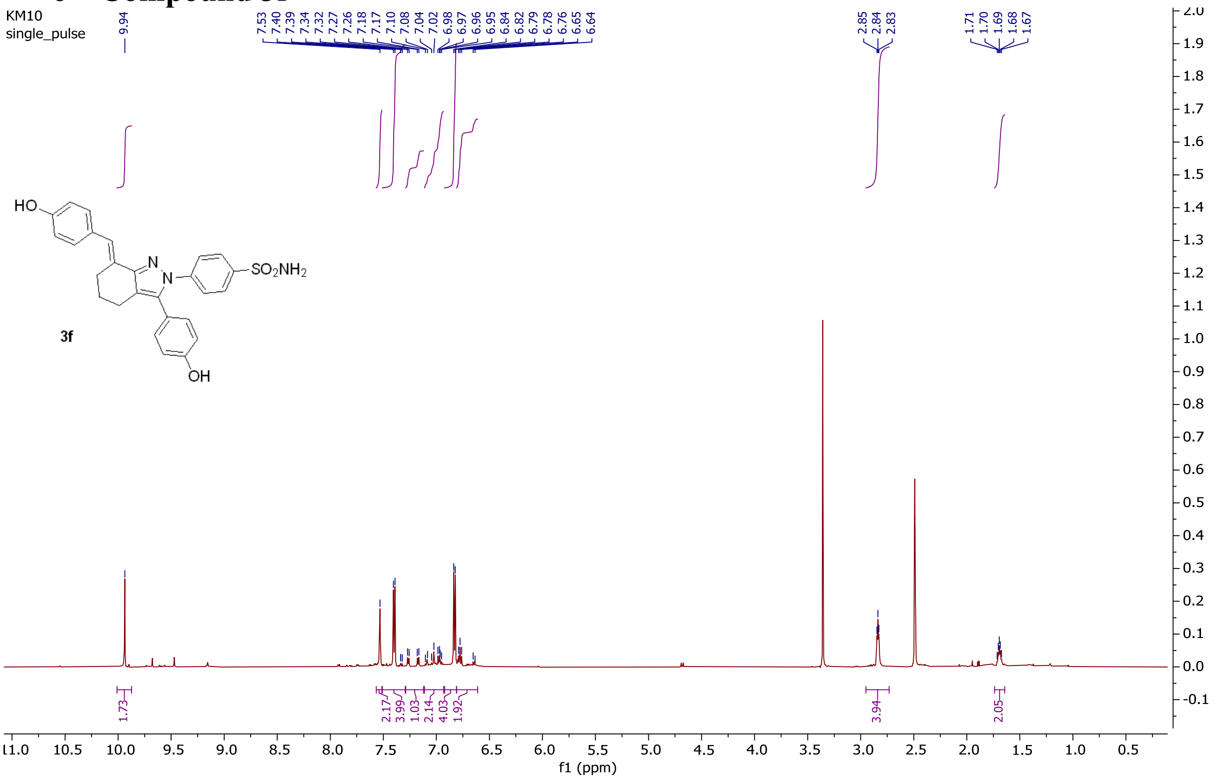


**Supplementary Figure S30. $^1$H NMR spectrum of compound 3f**

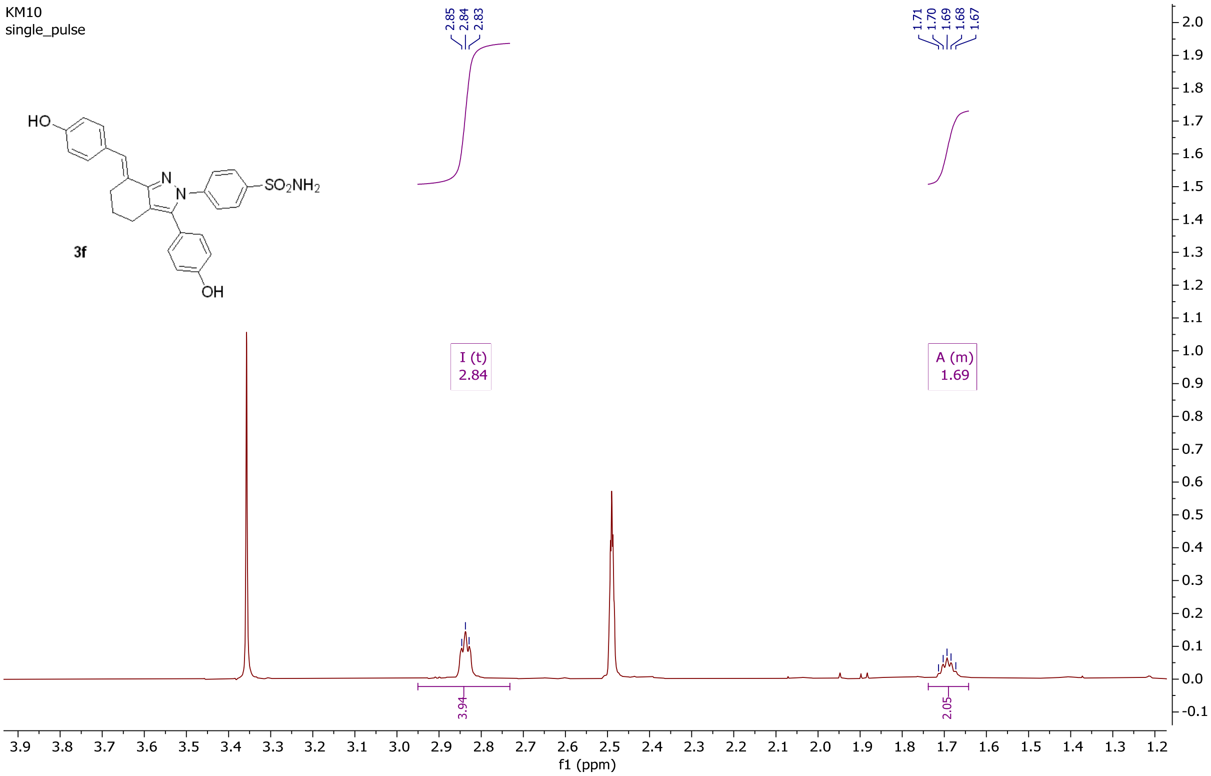


# Supplementary Figure S31. Expanded $^{1}$H NMR spectrum of compound 3f

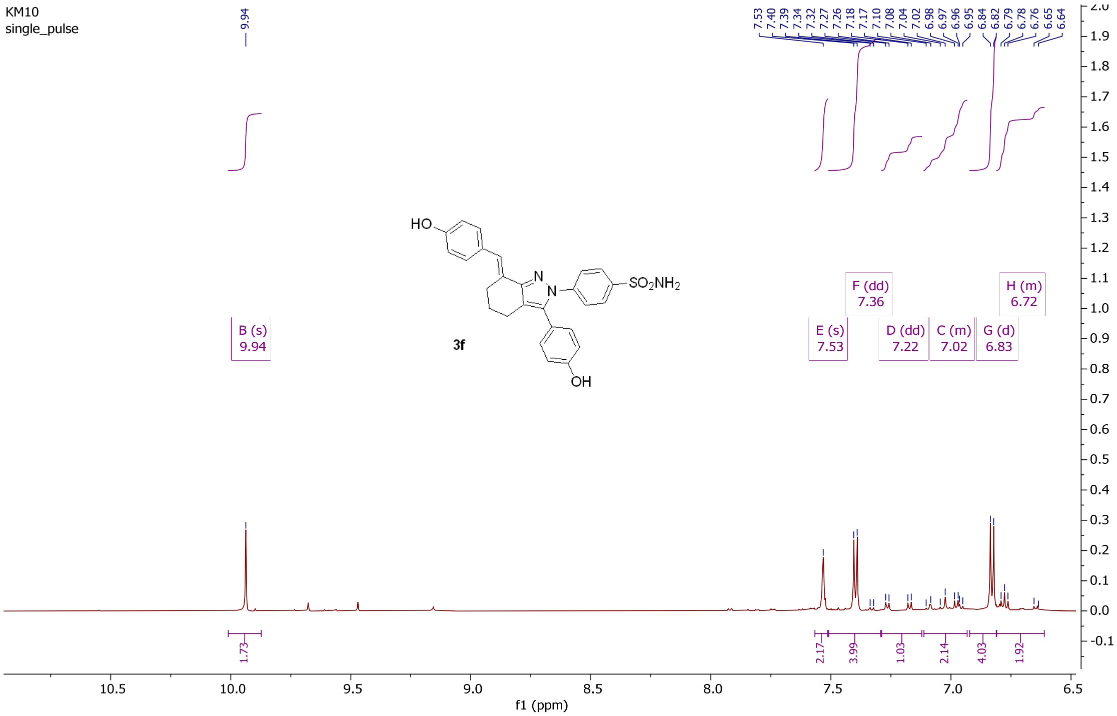


# Supplementary Figure S32. Expanded $^{1}$H NMR spectrum of compound 3f

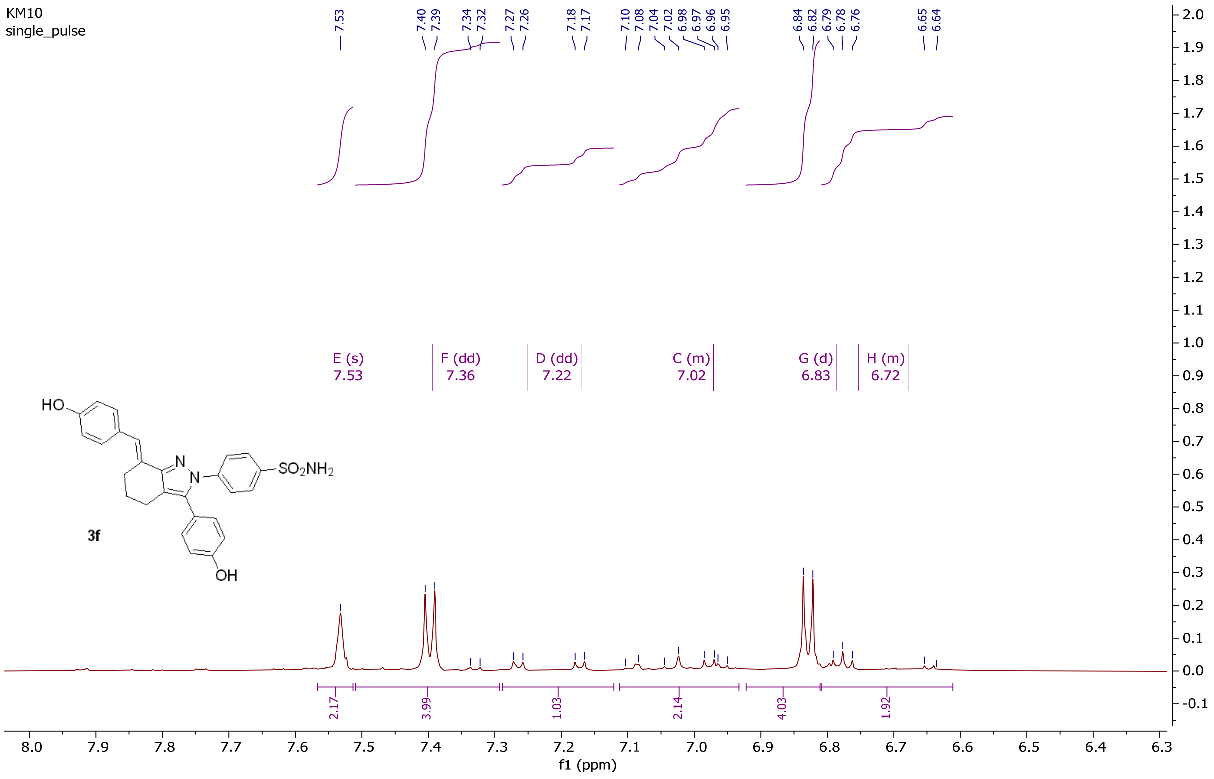


## Supplementary Figure S33. Expanded $^{1}$H NMR spectrum of compound 3f

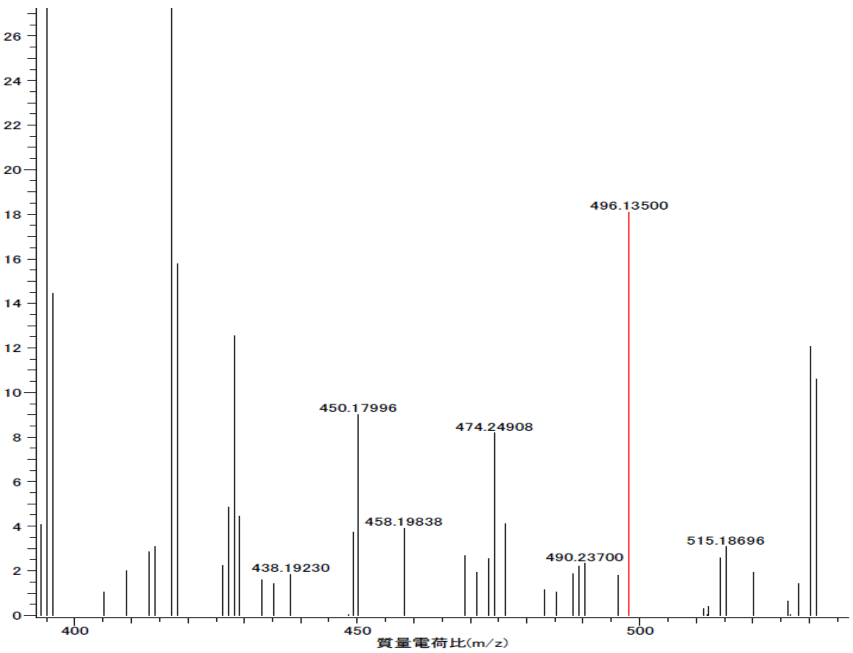


## Supplementary Figure S34. ESI-HRMS spectrum of compound 3f

## 7- Compound 3g

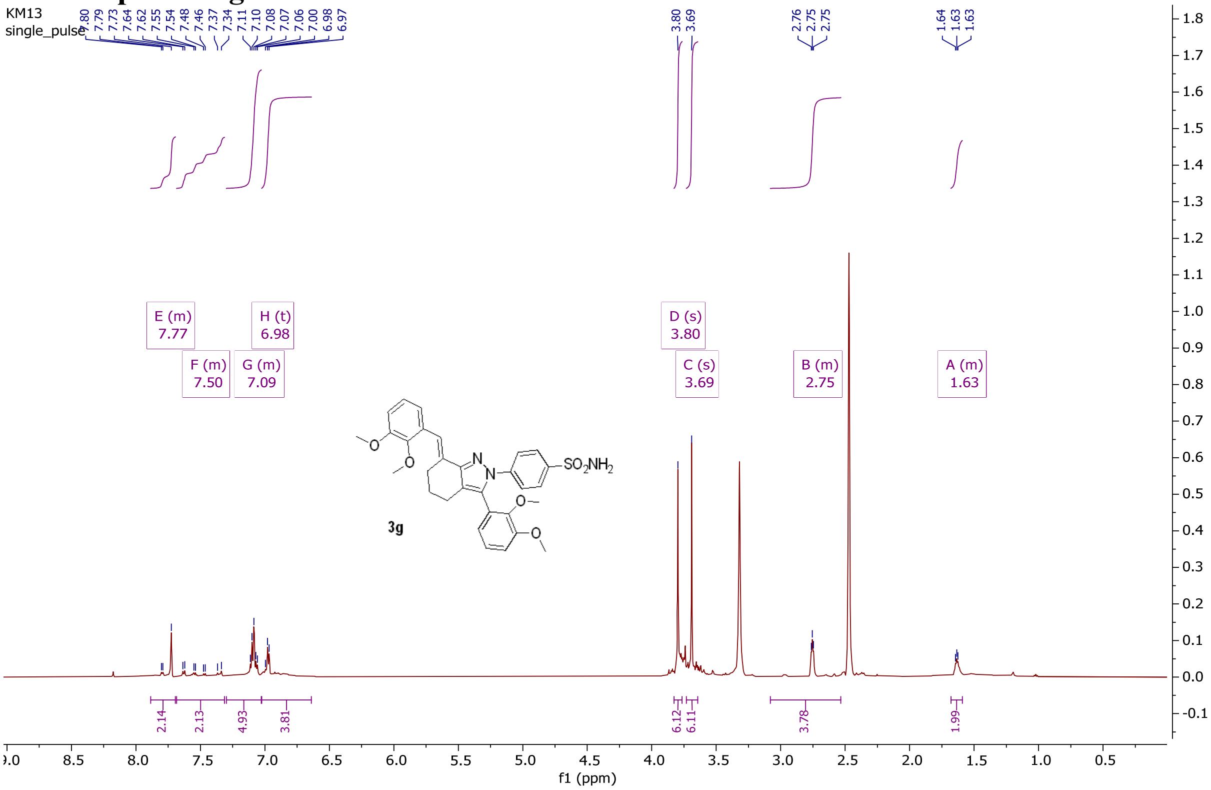


**Supplementary Figure S35. ¹H NMR spectrum of compound 3g**

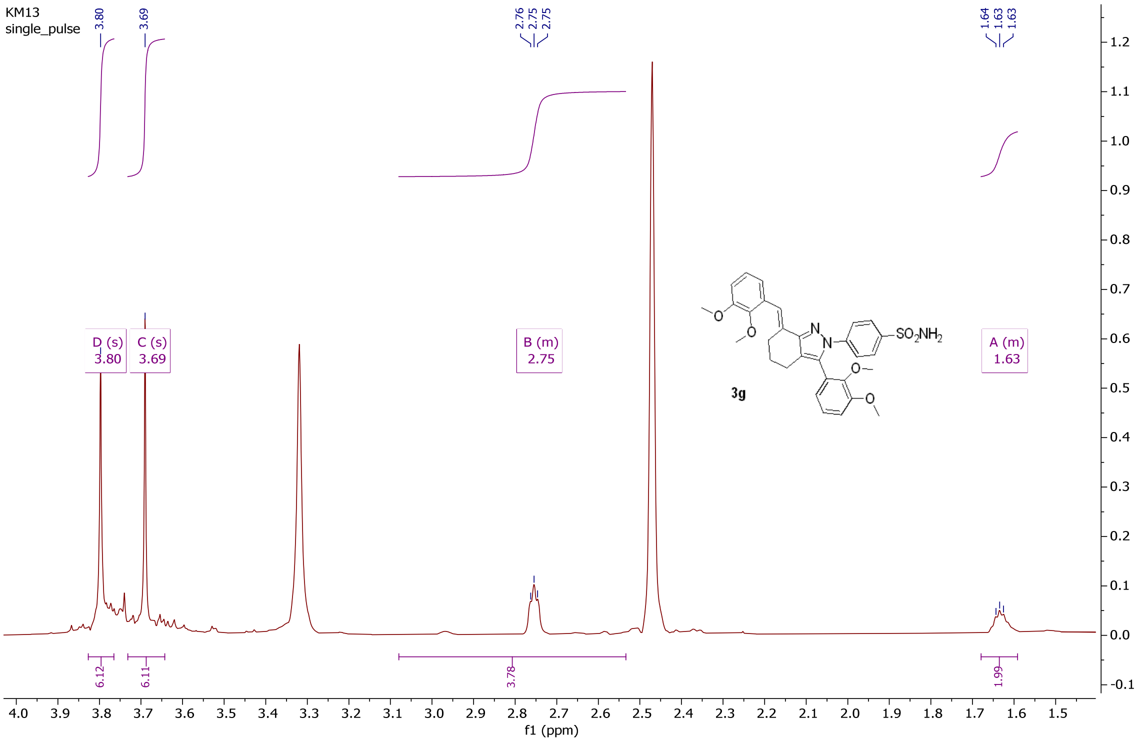


**Supplementary Figure S36. Expanded ¹H NMR spectrum of compound 3g**

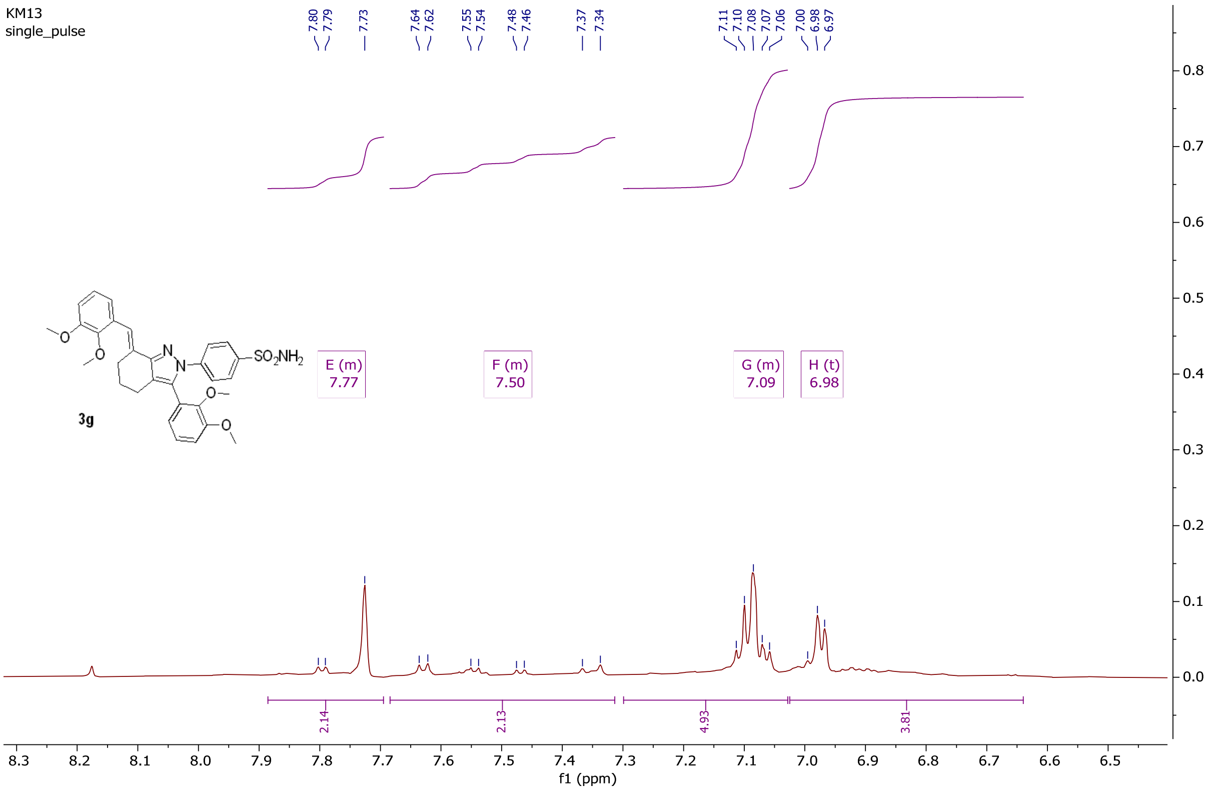


Supplementary Figure S37. Expanded $^{1}$H NMR spectrum of compound 3g

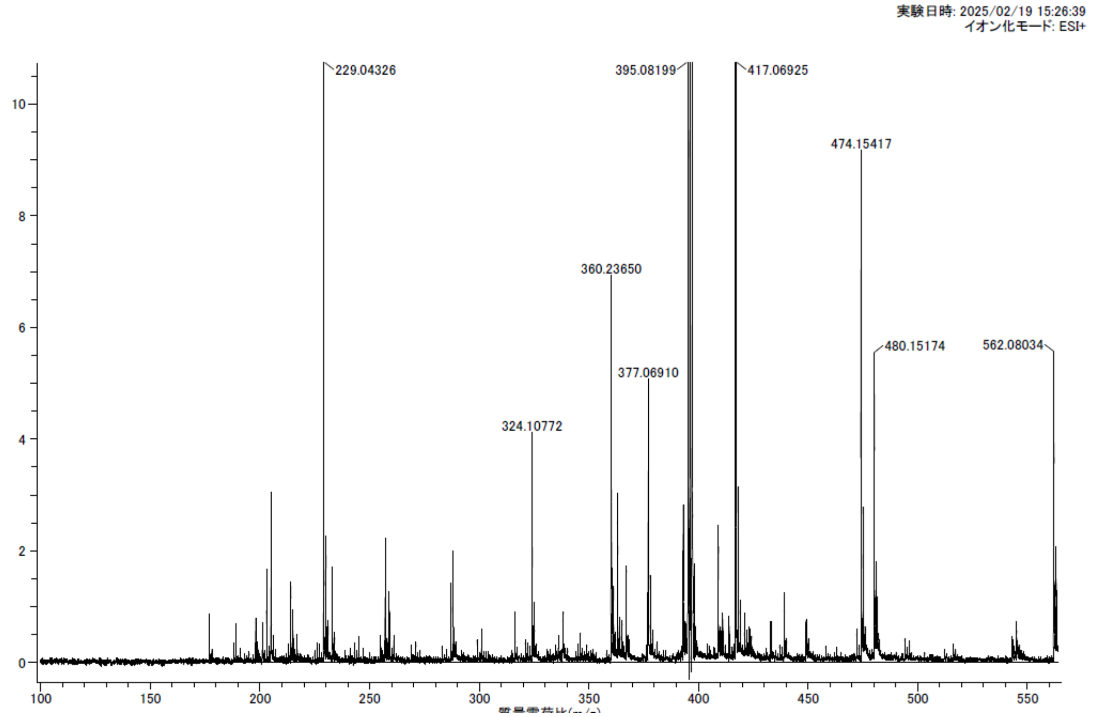


Supplementary Figure S38. LCMS spectrum of compound 3g

## 8- Compound 4a

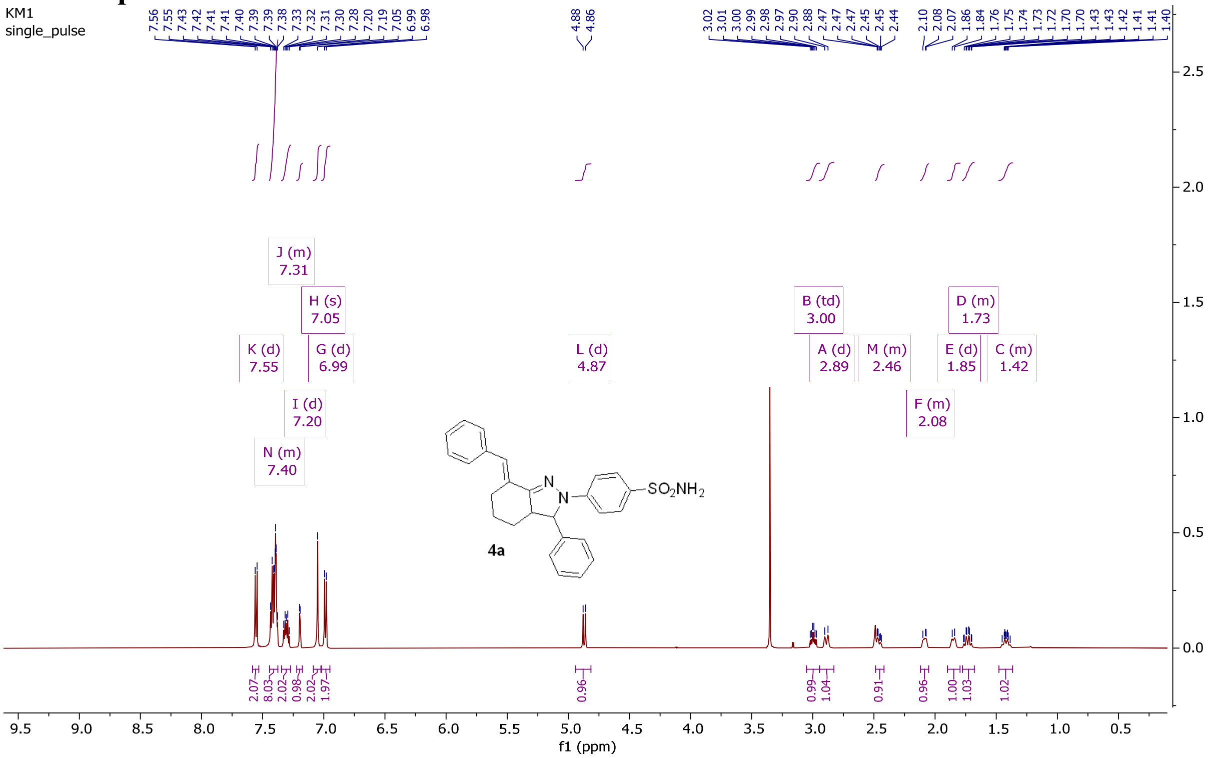


**Supplementary Figure S39. $^1$H NMR spectrum of compound 4a**

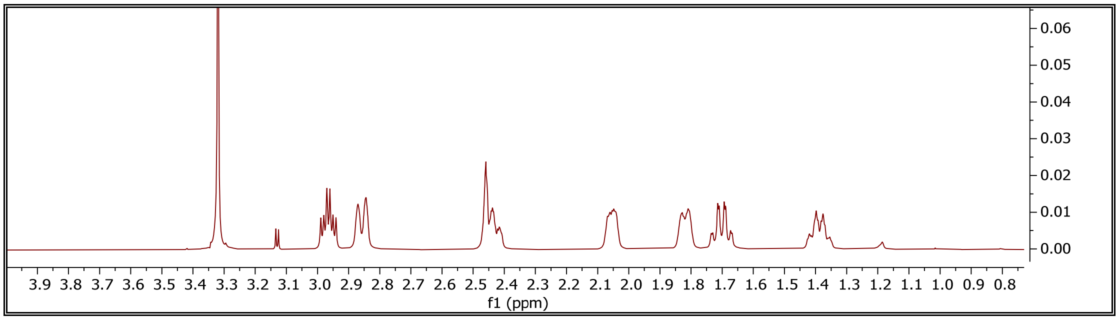


**Supplementary Figure S40. Expanded $^1$H NMR spectrum of compound 4a**

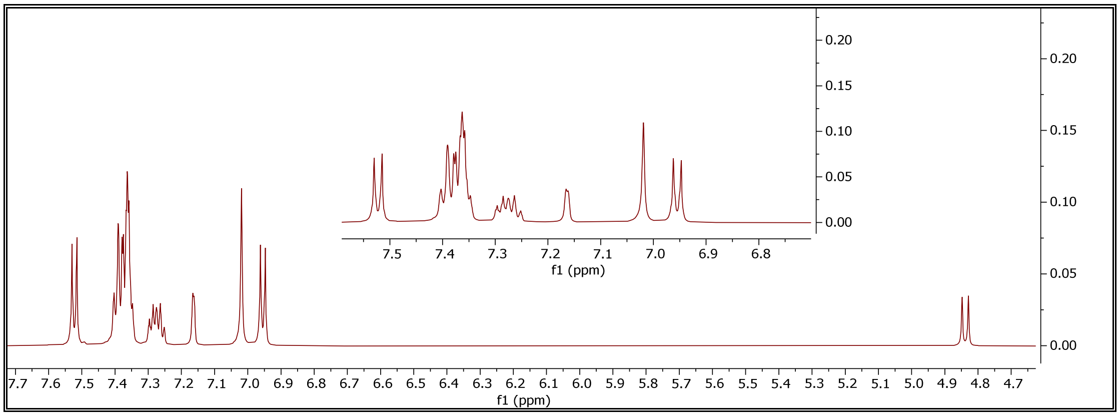


## Supplementary Figure S41. Expanded $^{1}$H NMR spectrum of compound 4a

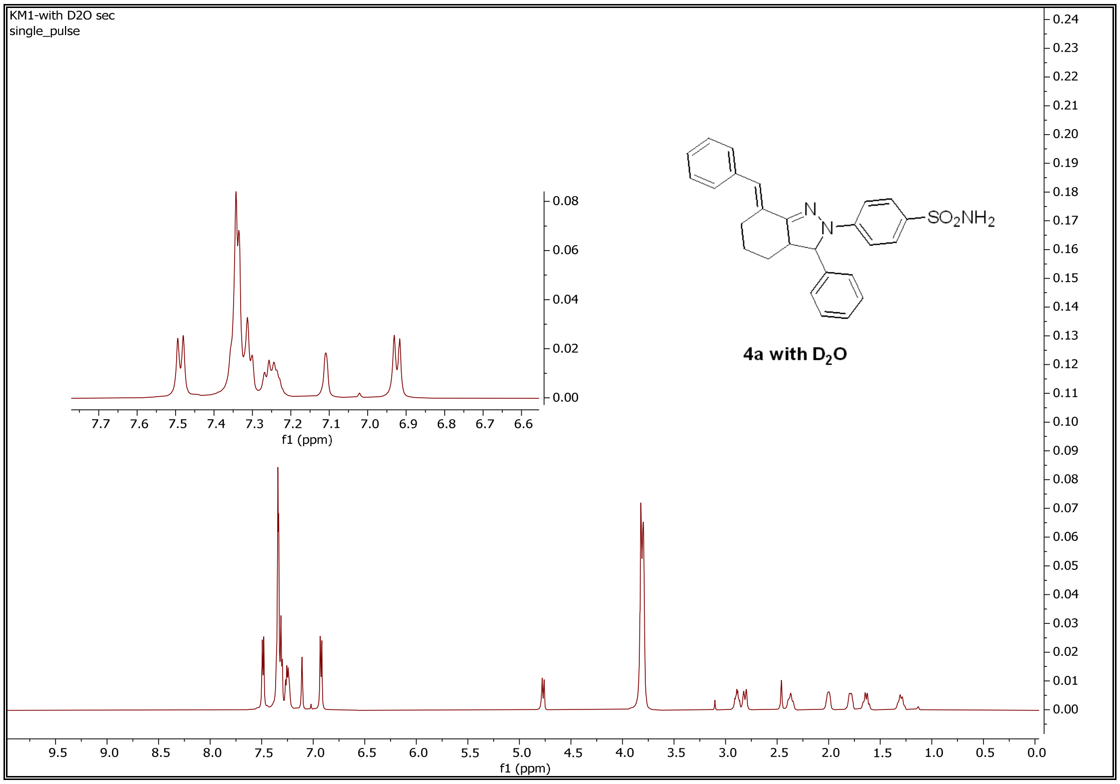


## Supplementary Figure S42. Expanded $^{1}$H NMR spectrum of compound 4a

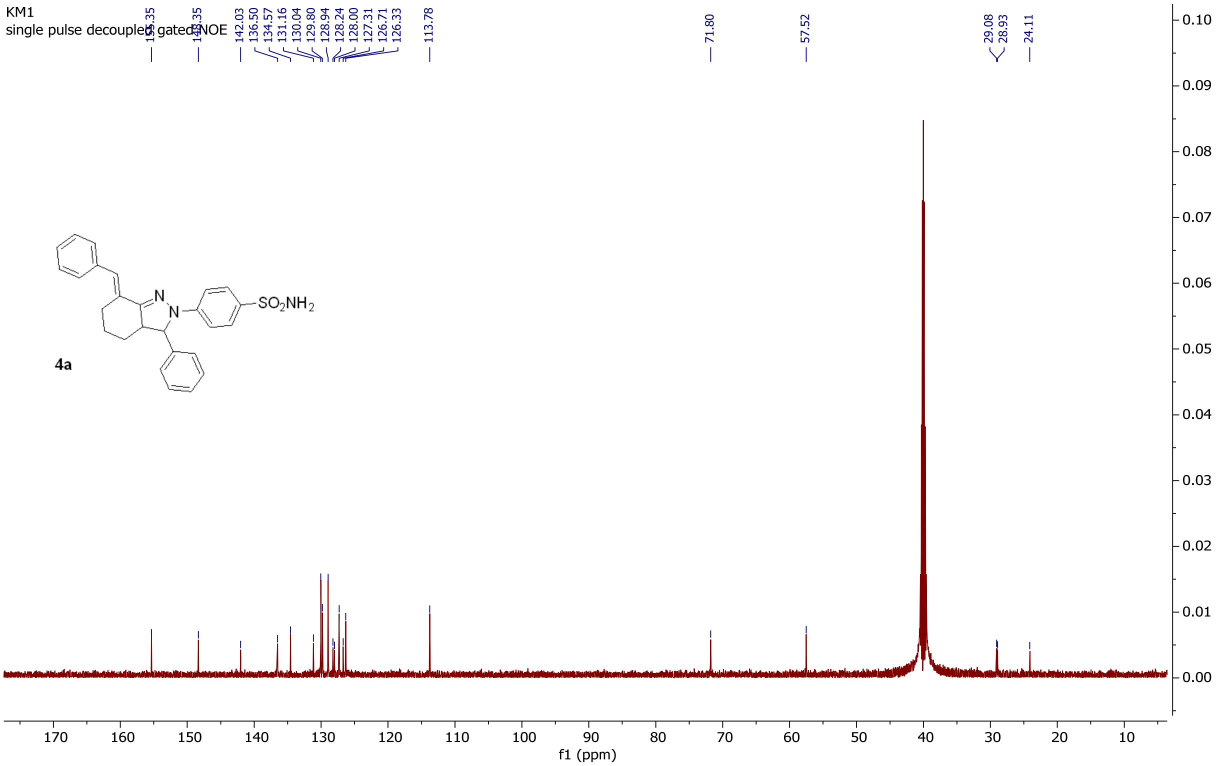


## Supplementary Figure S43. $^{13}C$ NMR spectrum of compound 4a

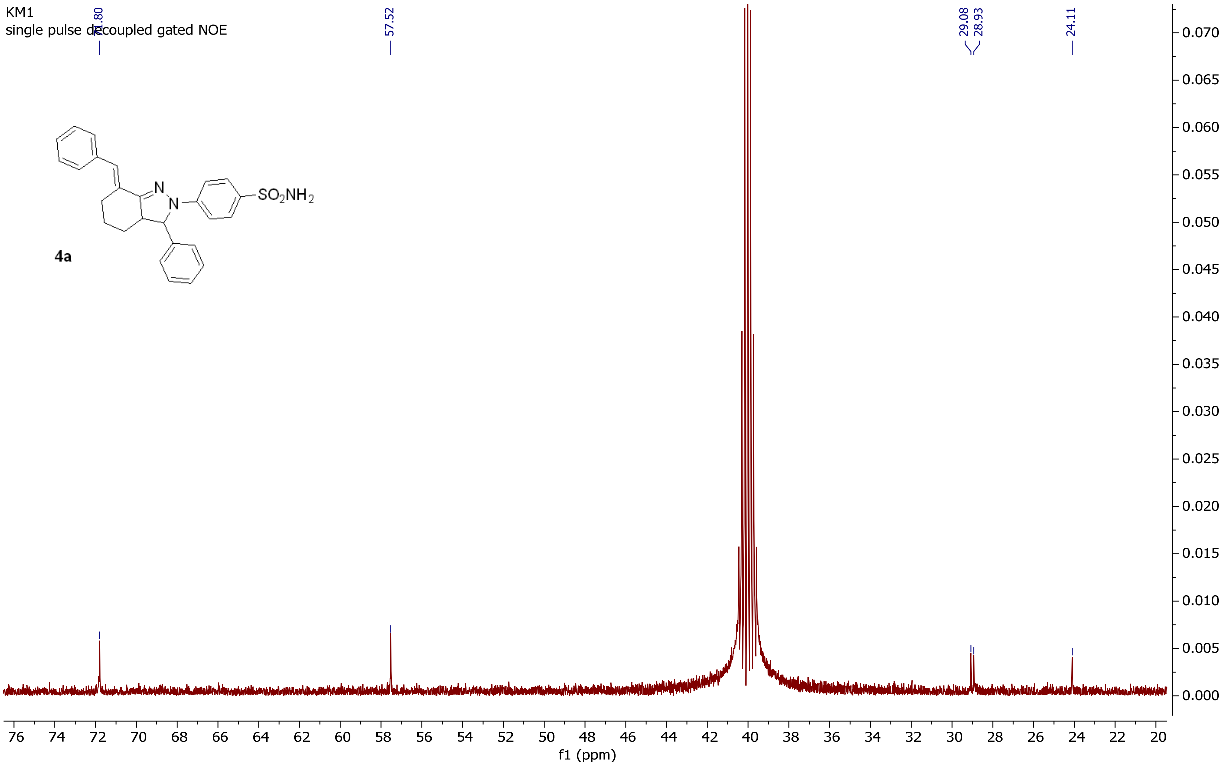


## Supplementary Figure S44. Expanded $^{13}C$ NMR spectrum of compound 4a

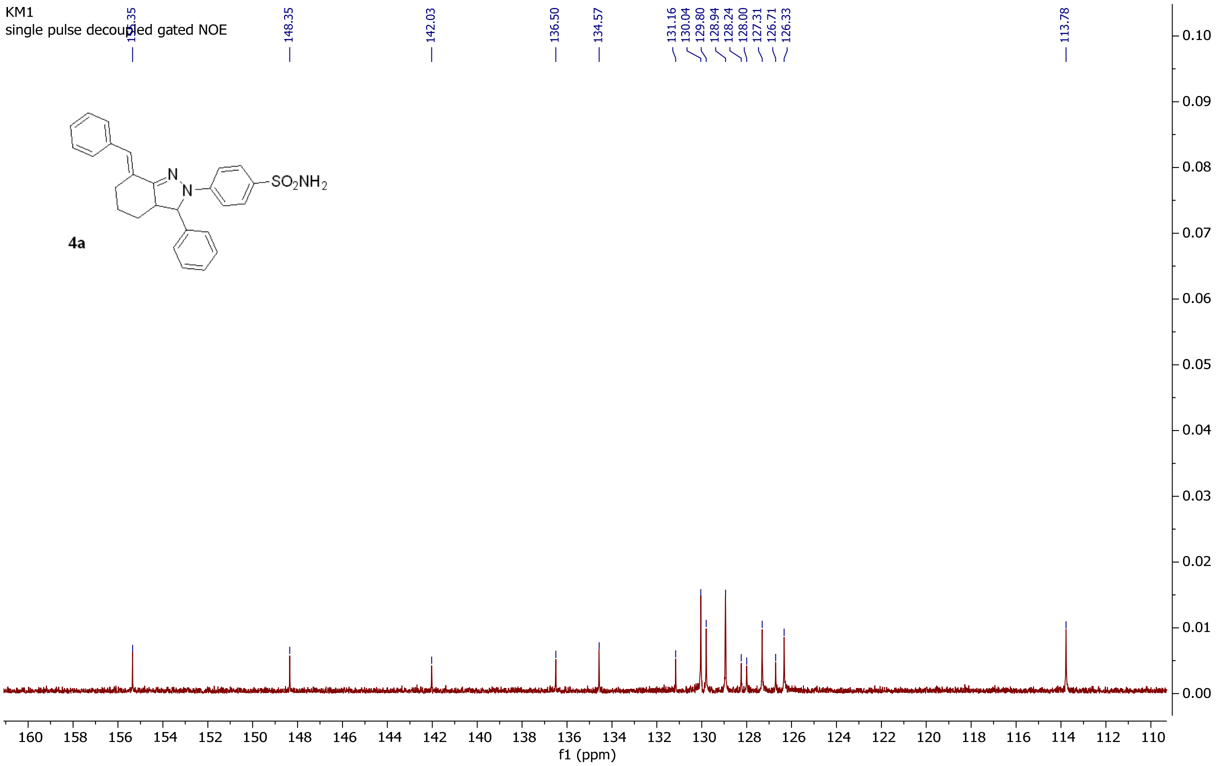


# Supplementary Figure S45. Expanded $^{13}$C NMR spectrum of compound 4a

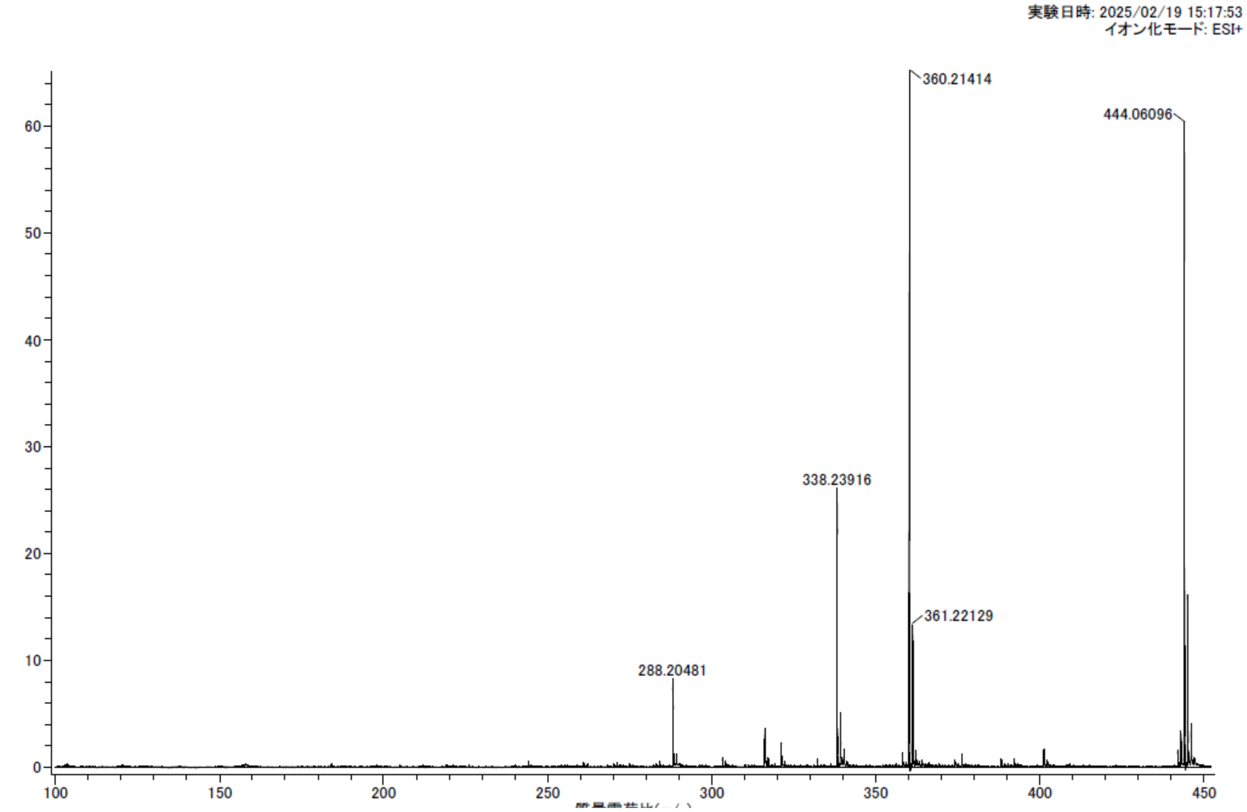


# Supplementary Figure S46. LCMS spectrum of compound 4a

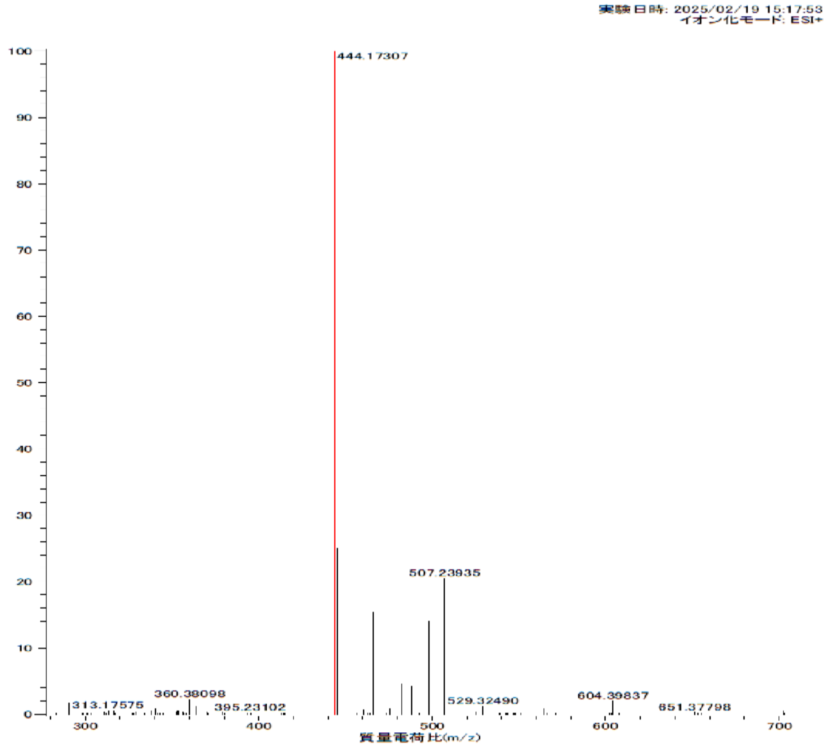


**Supplementary Figure S47. ESI-HRMS spectrum of compound 4a**

## 9- Compound 4b

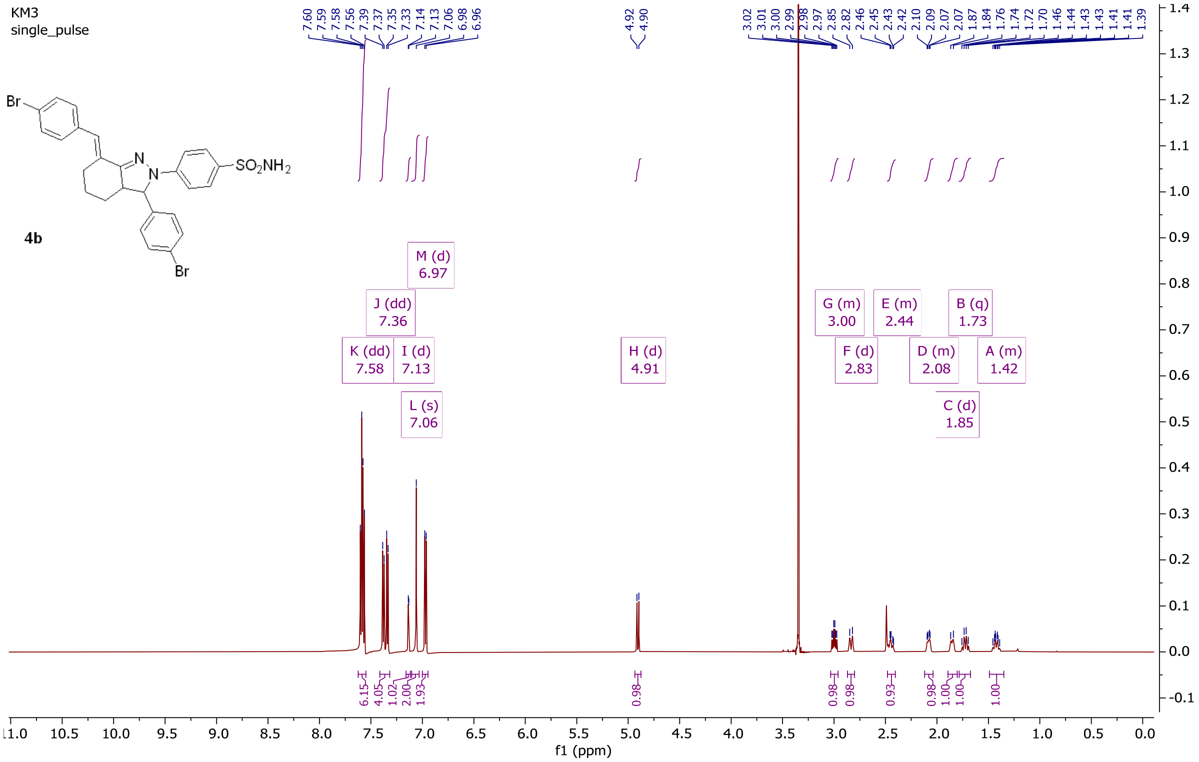


**Supplementary Figure S48. $^{1}$H NMR spectrum of compound 4b**

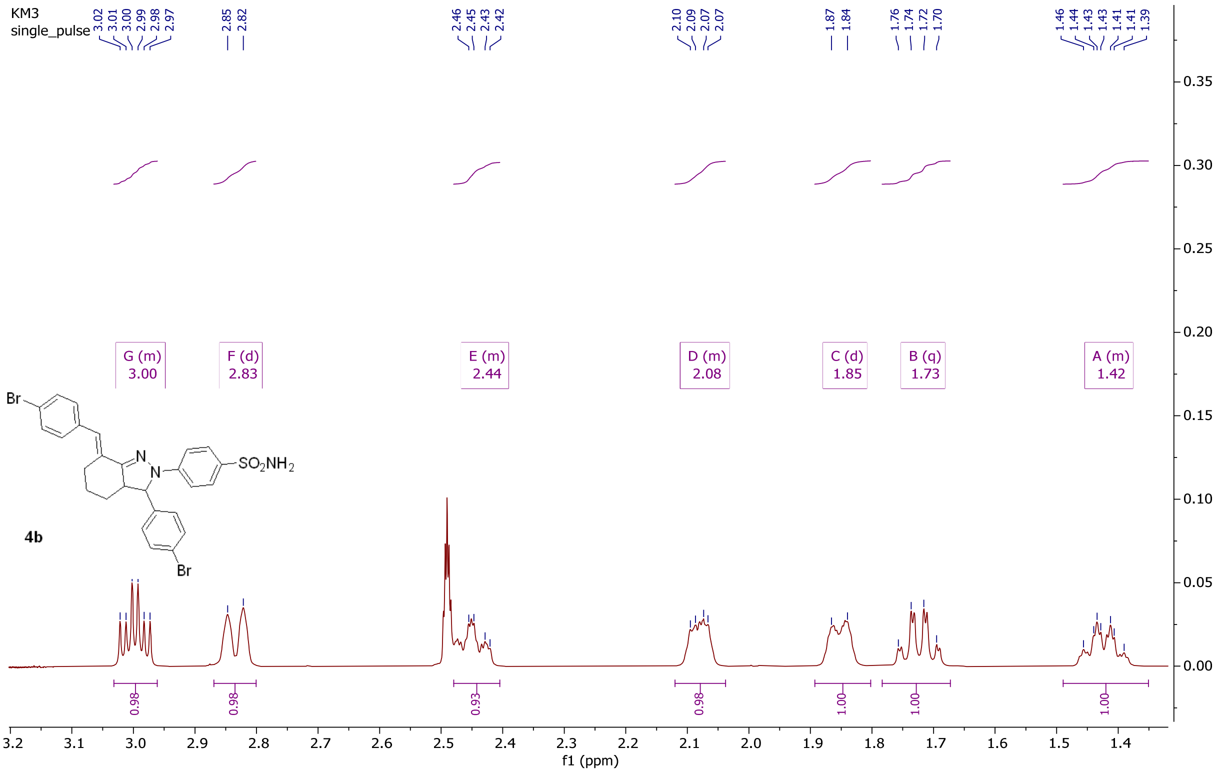

**Supplementary Figure S49. Expanded $^1$H NMR spectrum of compound 4b**

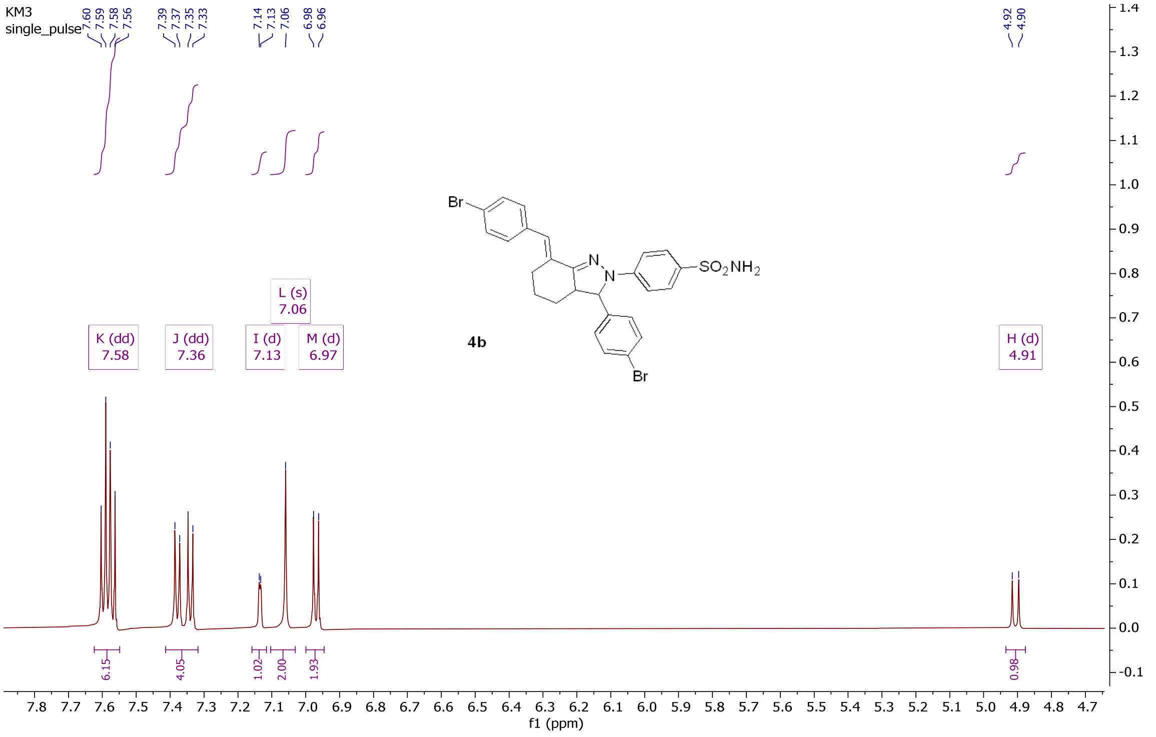

**Supplementary Figure S50. Expanded $^1$H NMR spectrum of compound 4b**

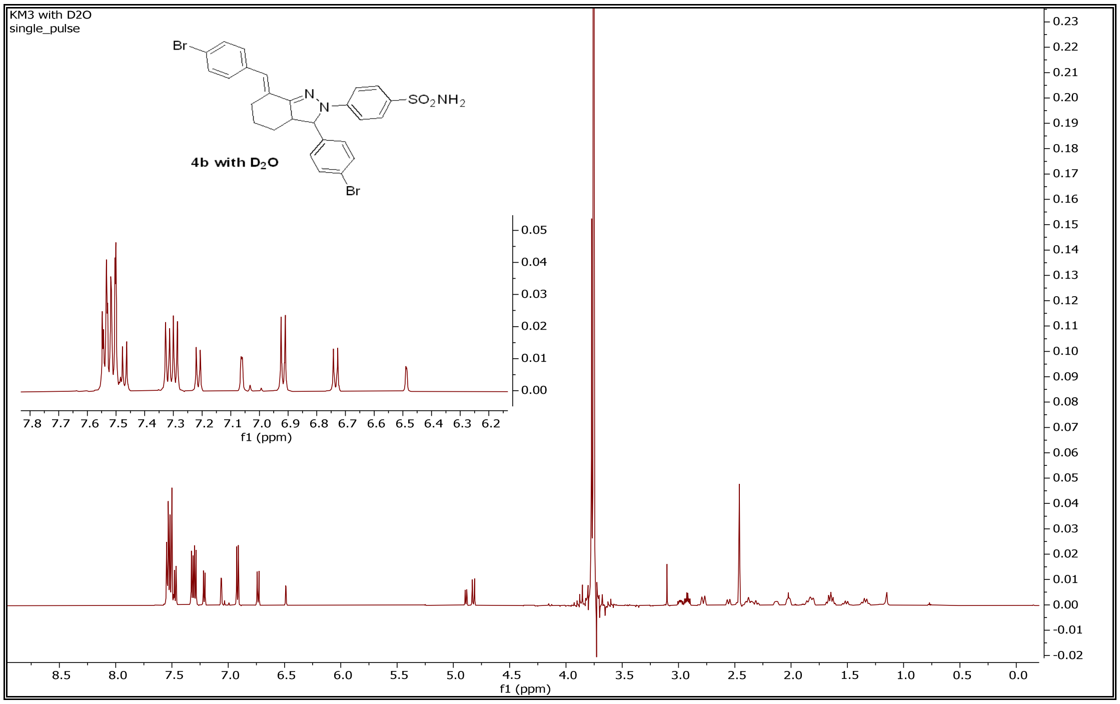


**Supplementary Figure S51. ¹H NMR spectrum of compound 4b with D2O**

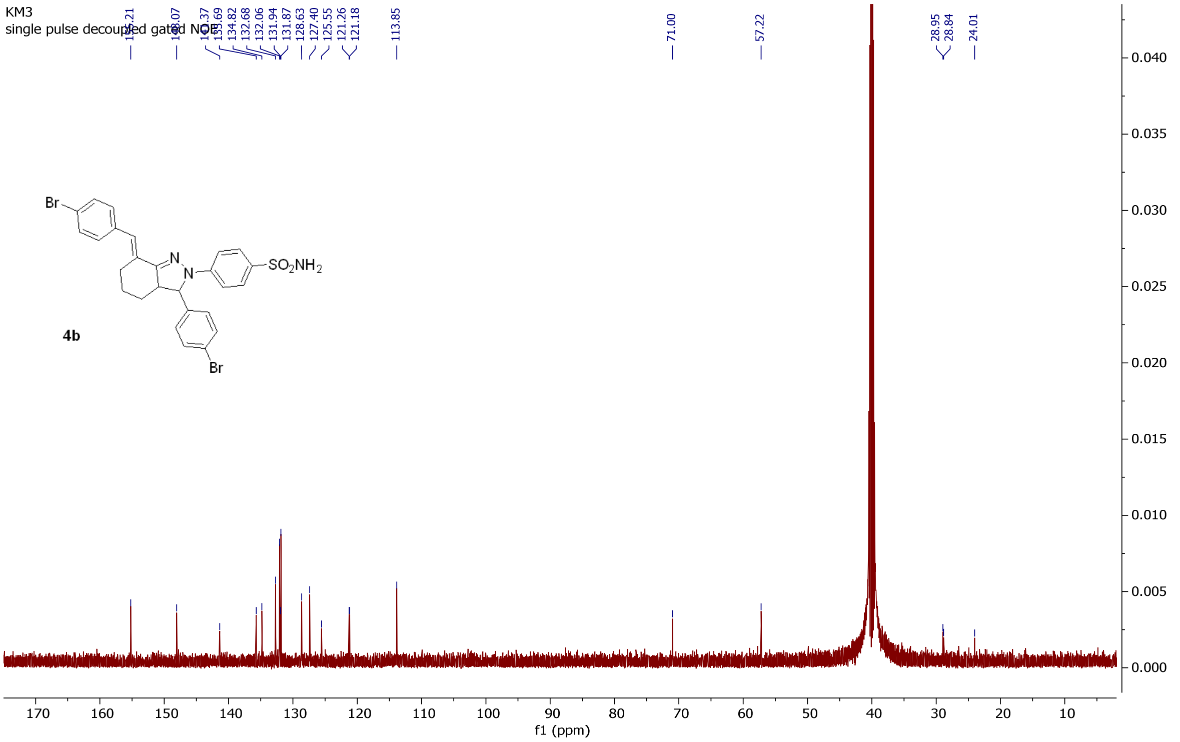


**Supplementary Figure S52. ¹³C NMR spectrum of compound 4b**

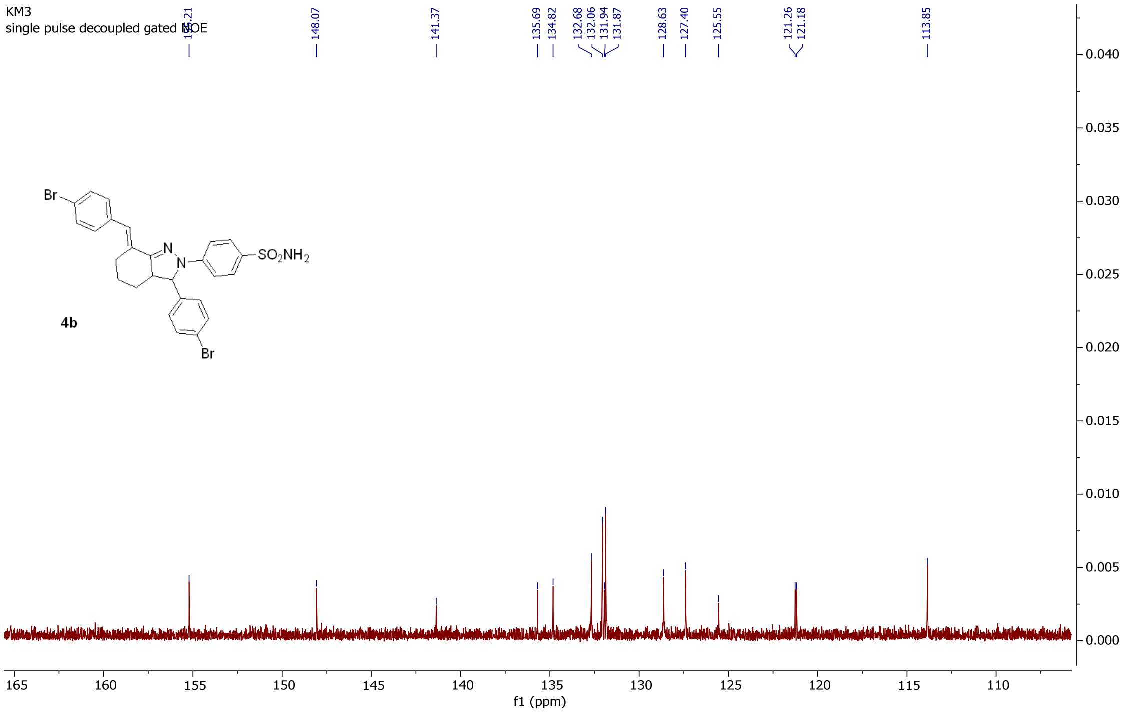


**Supplementary Figure S53. Expanded $^{13}$C NMR spectrum of compound 4b**

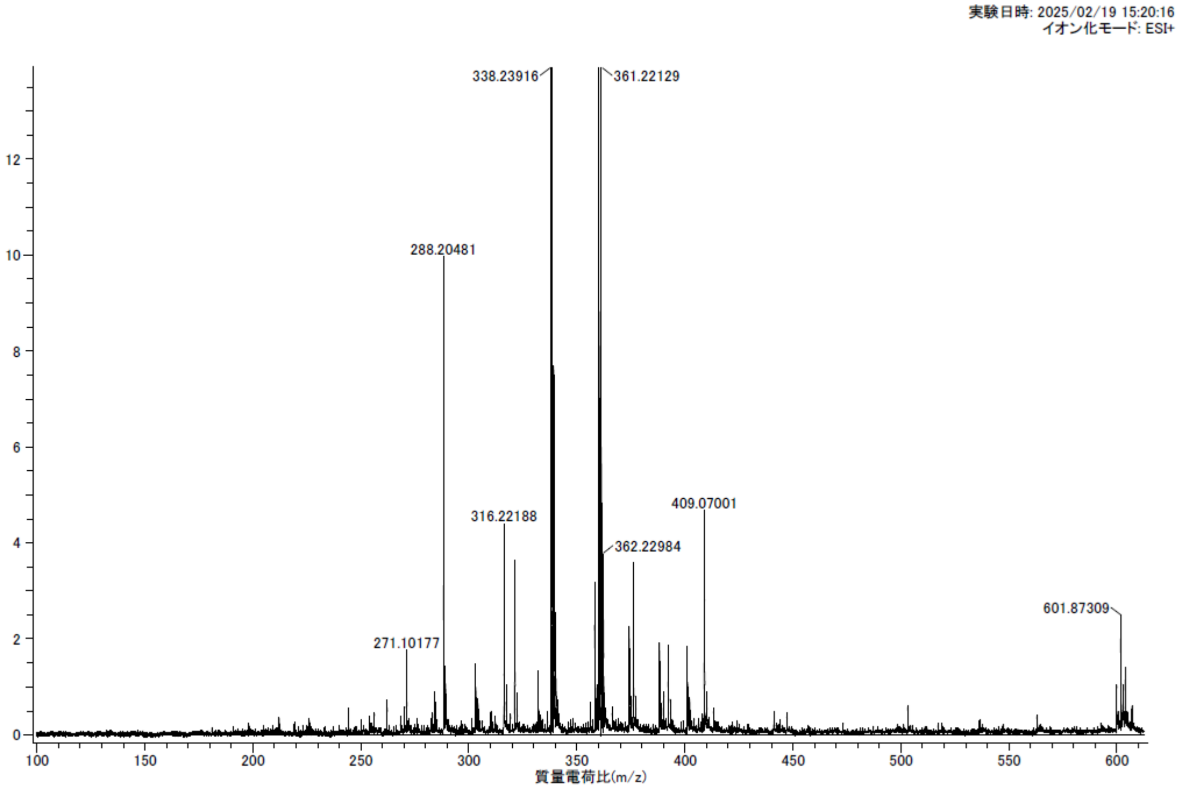


**Supplementary Figure S54. LCMS spectrum of compound 4b**

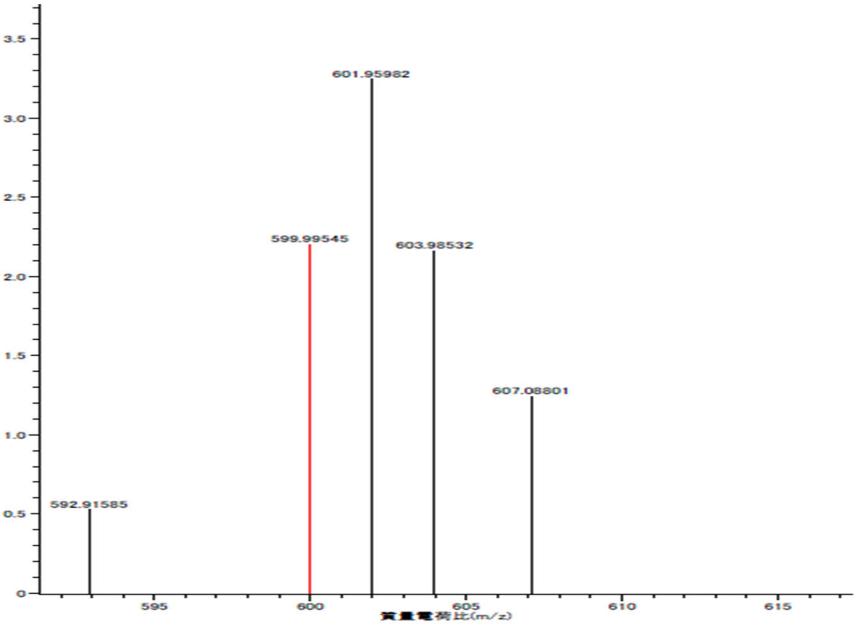


**Supplementary Figure S55. ESI-HRMS spectrum of compound 4b**

## 10- Compound 4c

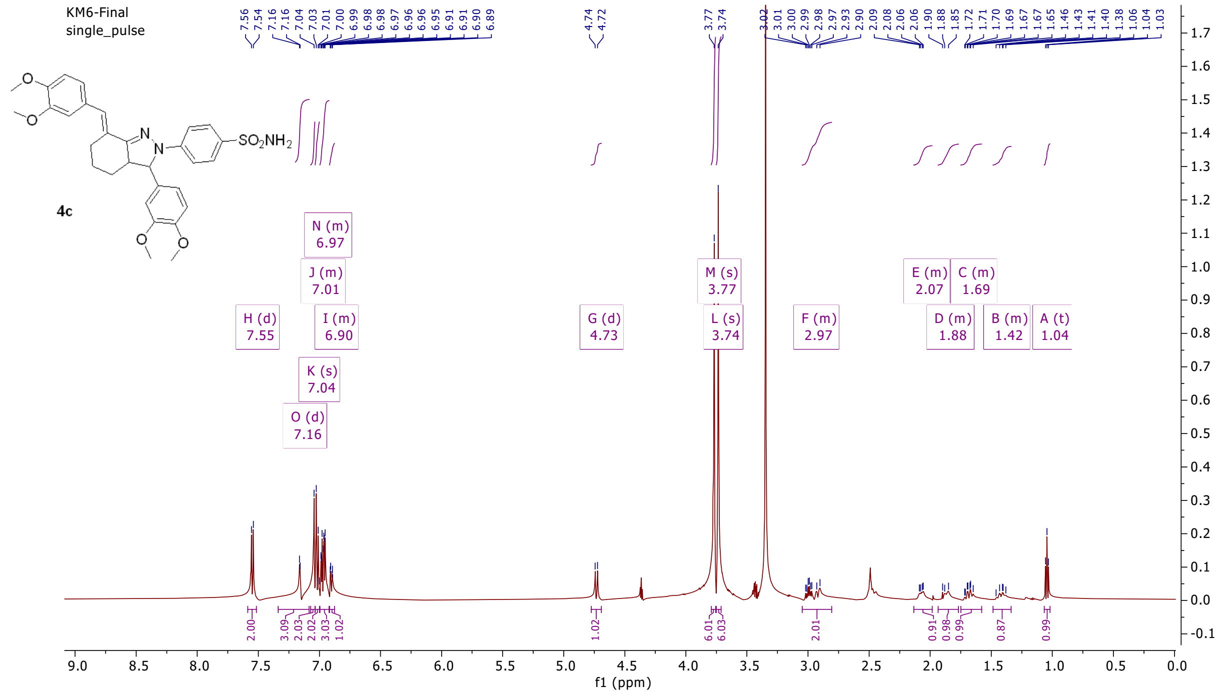


**Supplementary Figure S56. $^{1}$H NMR spectrum of compound 4c**

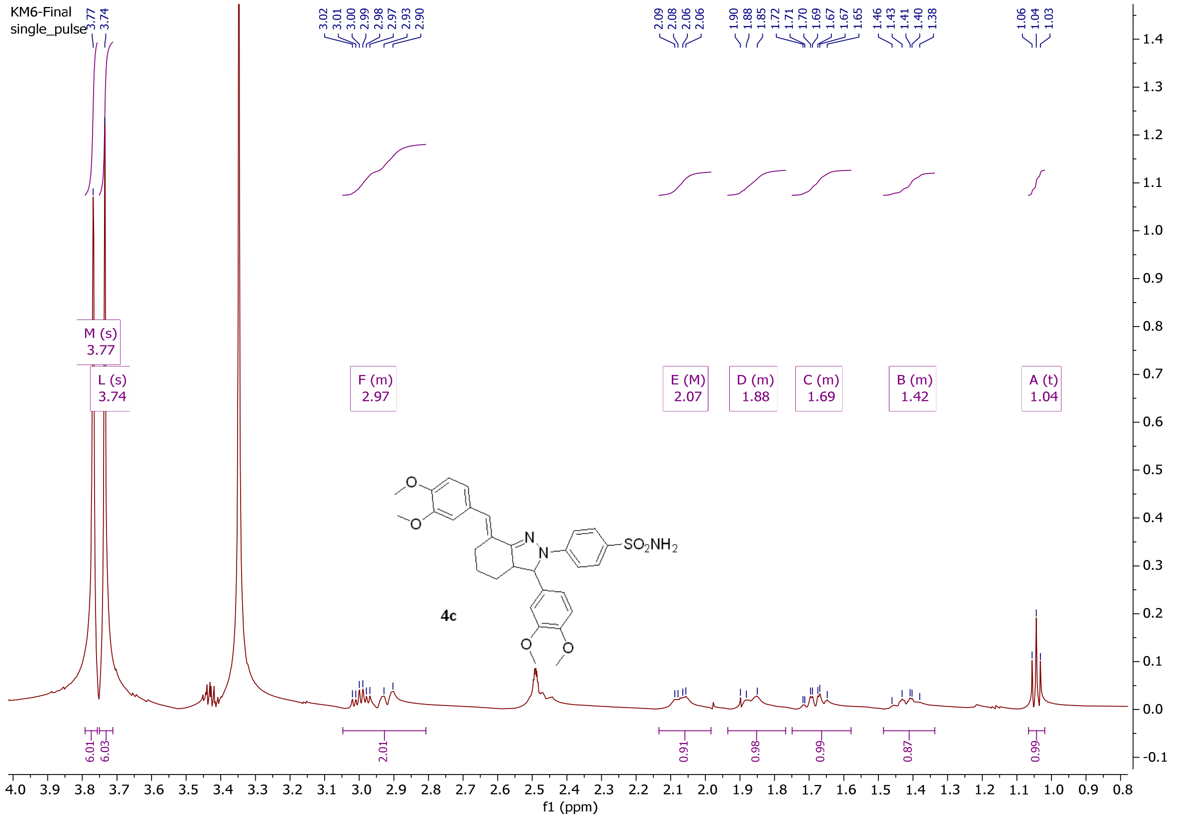

## Supplementary Figure S57. Expanded $^{1}H$ NMR spectrum of compound 4c

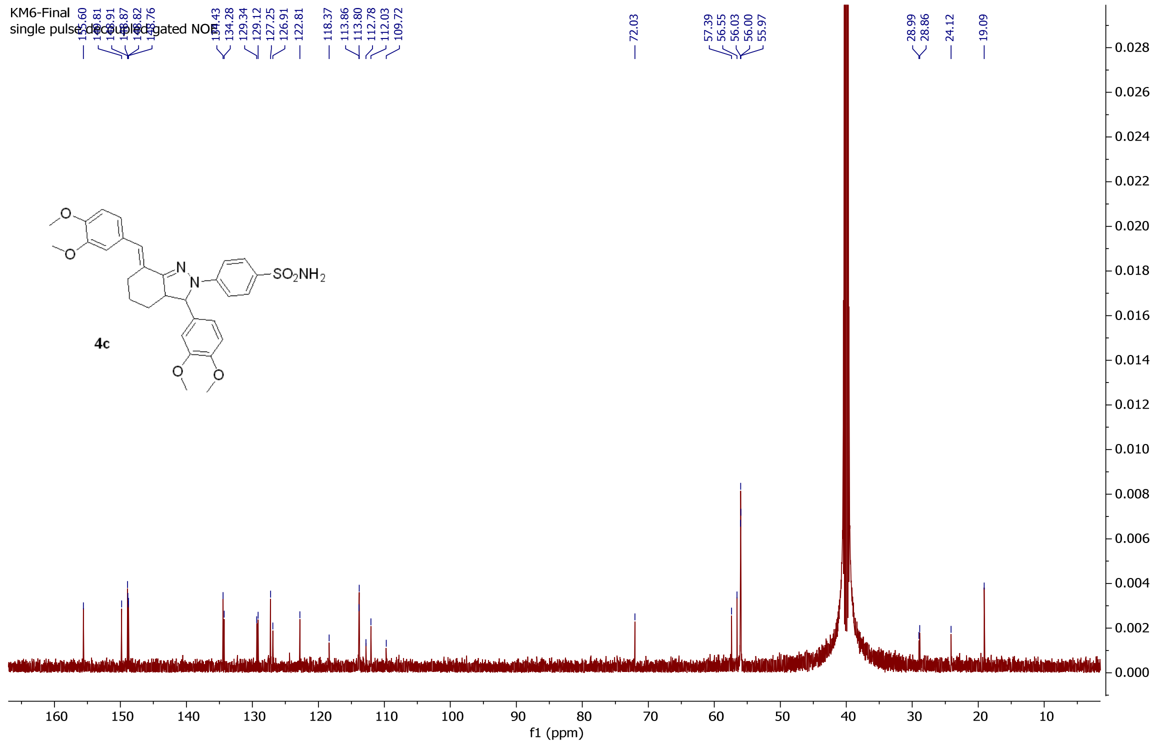

## Supplementary Figure S58. $^{13}C$ NMR spectrum of compound 4c

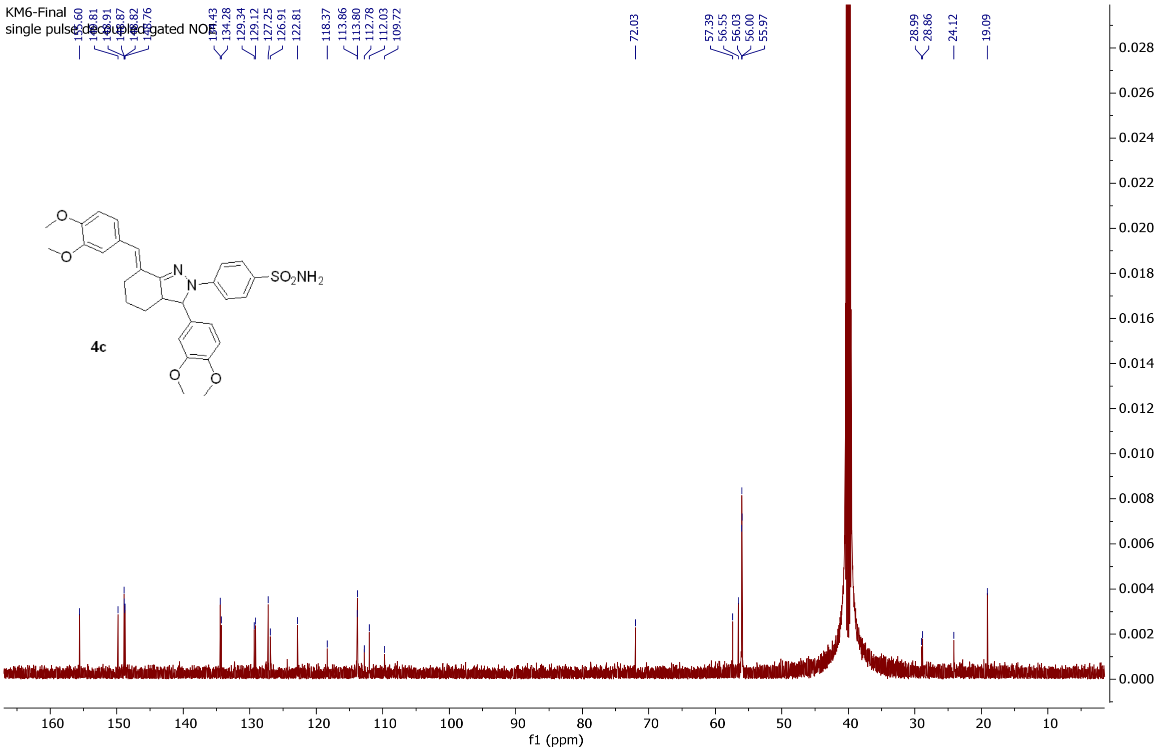


**Supplementary Figure S59. Expanded $^{13}C$ NMR spectrum of compound 4c**

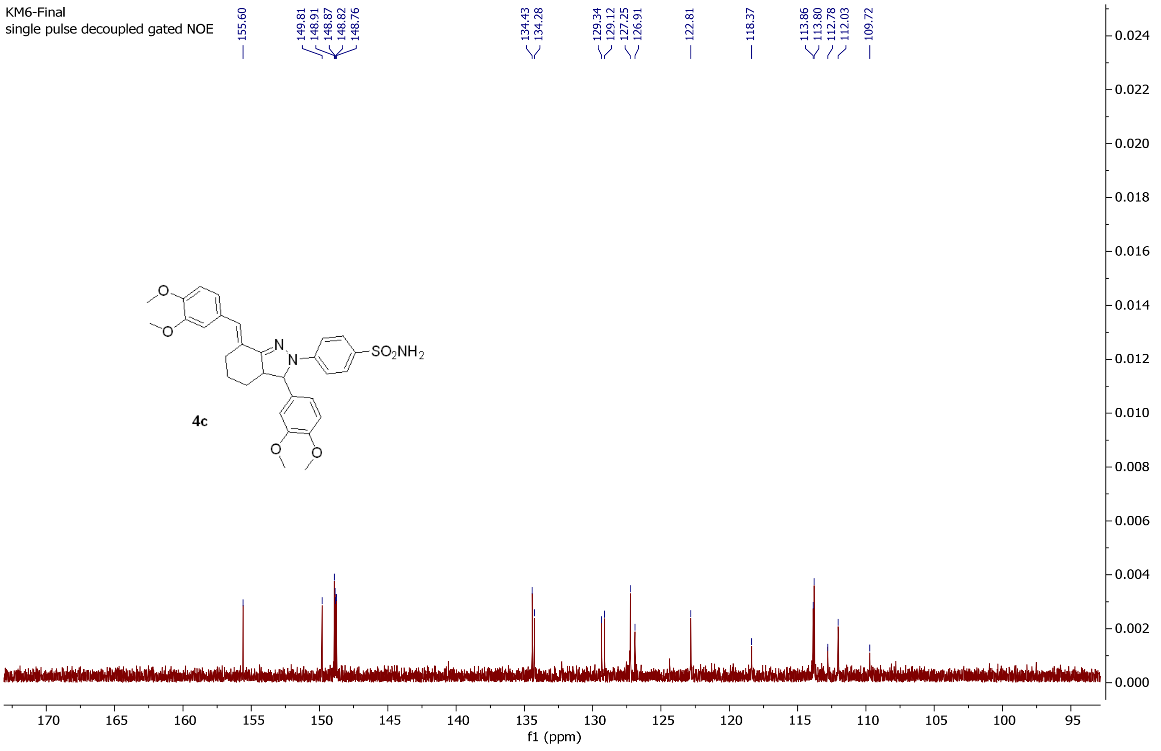


**Supplementary Figure S60. Expanded $^{13}C$ NMR spectrum of compound 4c**

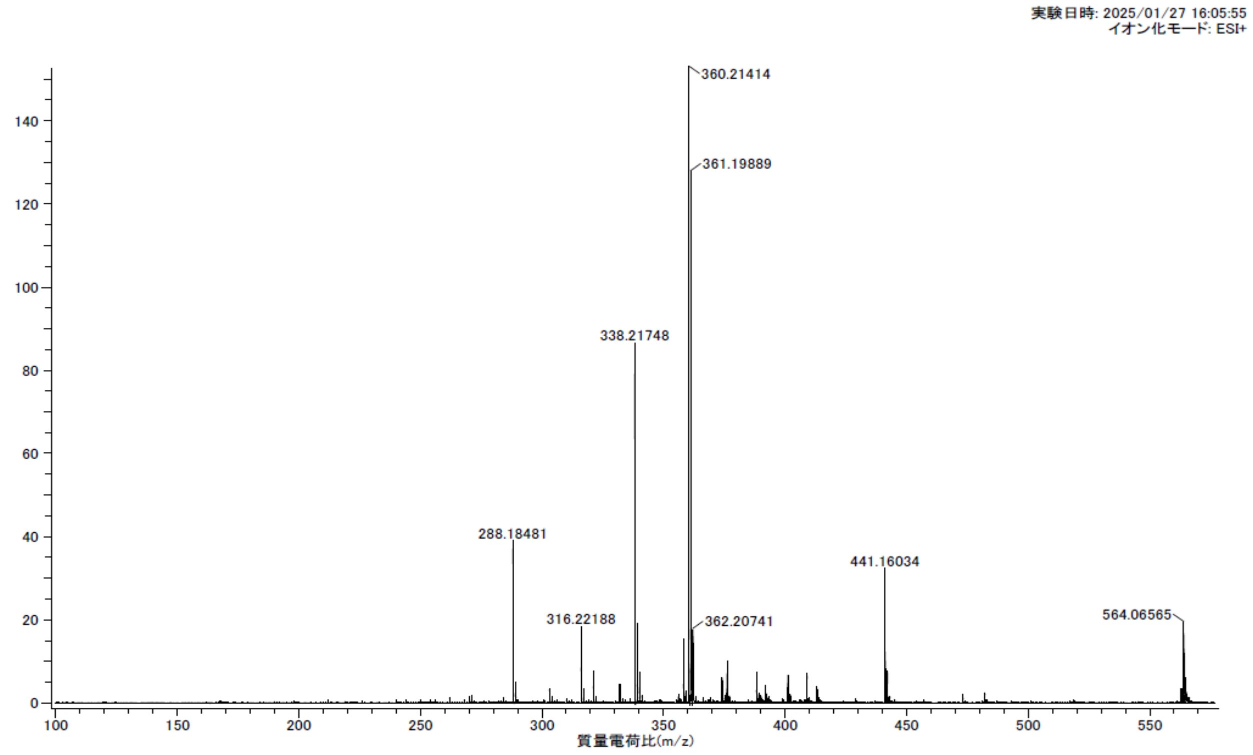


**Supplementary Figure S61. LCMS spectrum of compound 4c**

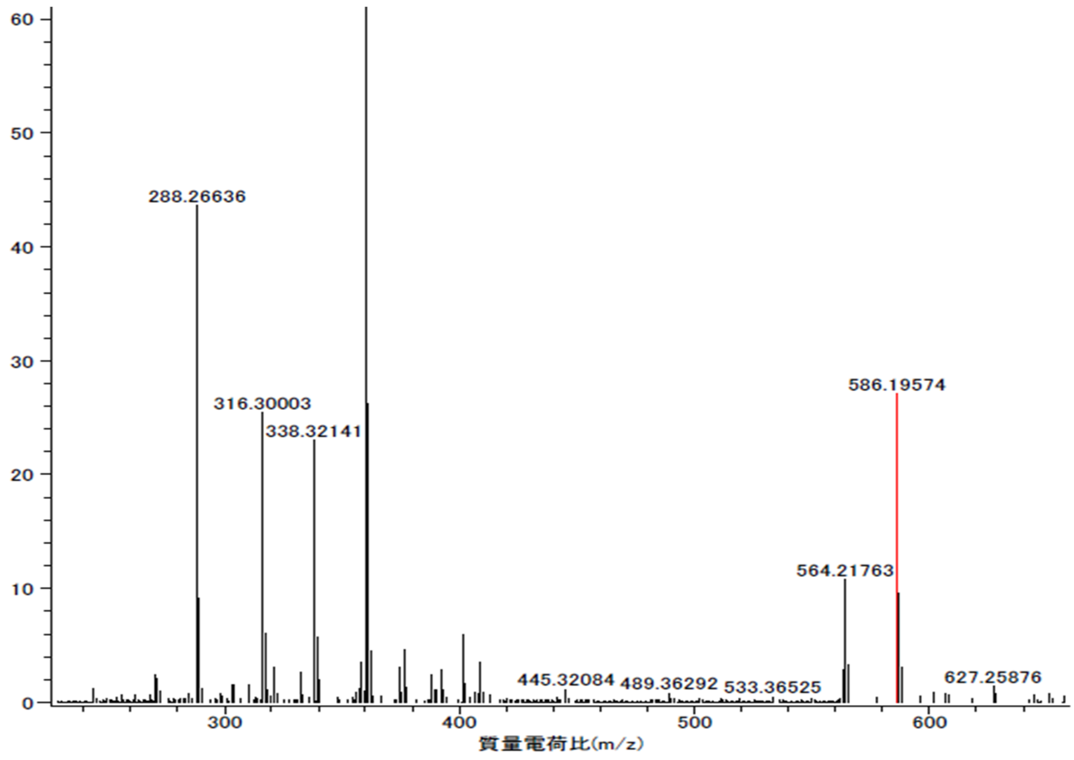


**Supplementary Figure S62. ESI-HRMS spectrum of compound 4c**

# 11- Compound 4d

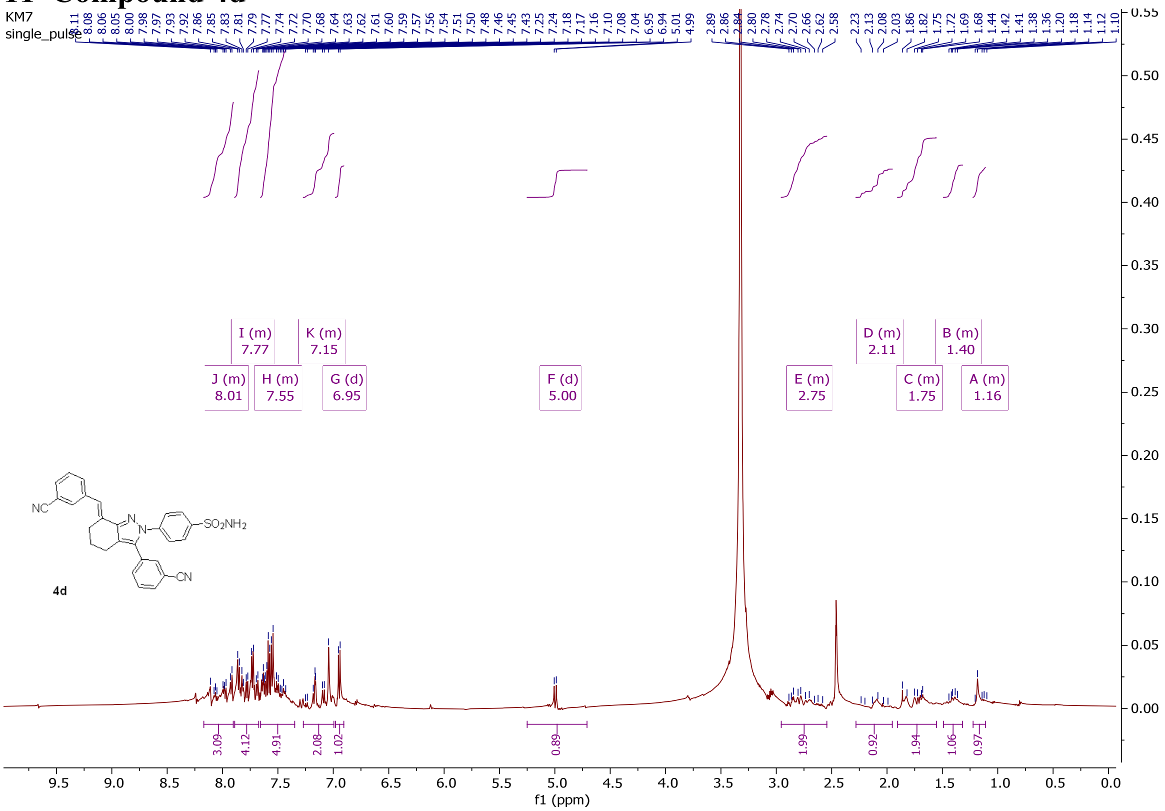


## Supplementary Figure S63. $^1$H NMR spectrum of compound 4d

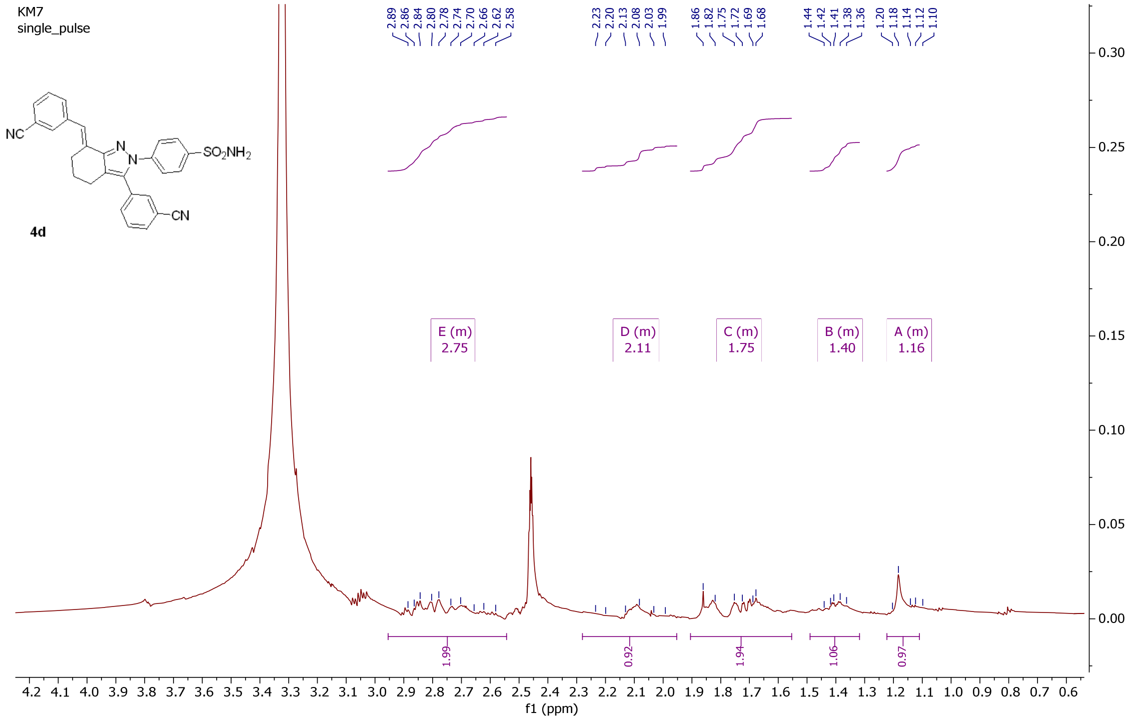


## Supplementary Figure S64. Expanded $^1$H NMR spectrum of compound 4d

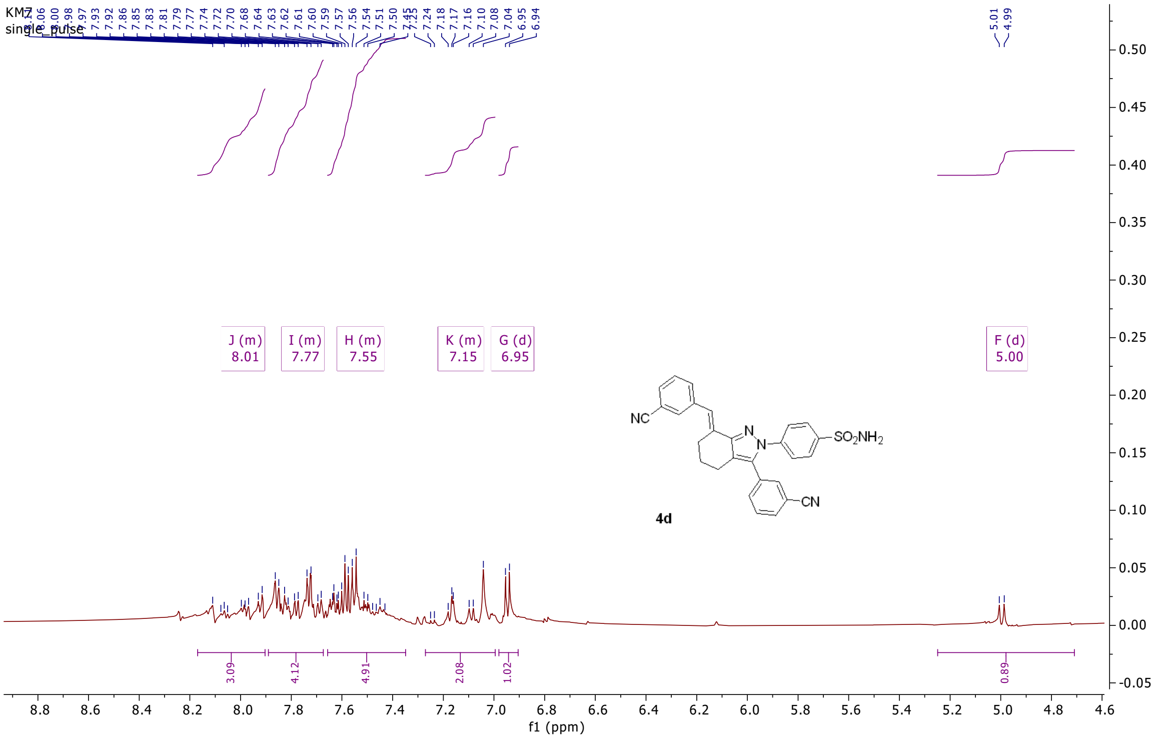

**Supplementary Figure S65. Expanded $^{1}H$ NMR spectrum of compound 4d**

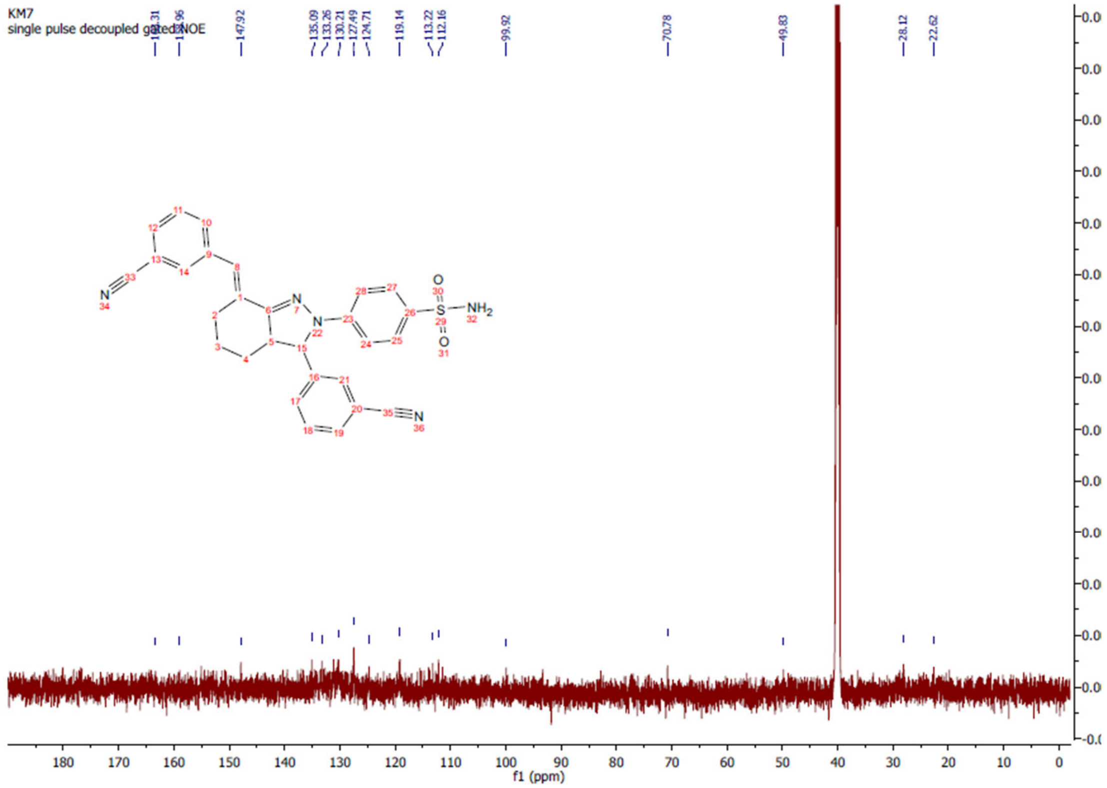

**Supplementary Figure S66. $^{13}C$ NMR spectrum of compound 4d**

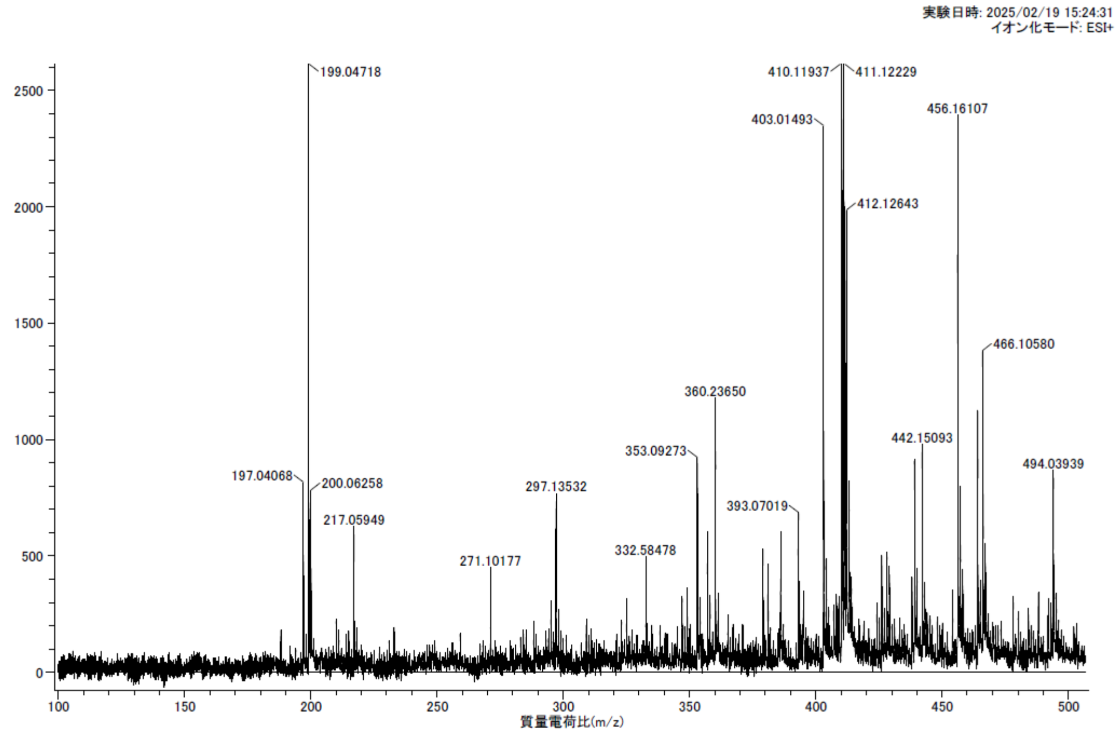


Supplementary Figure S67. LCMS spectrum of compound 4d

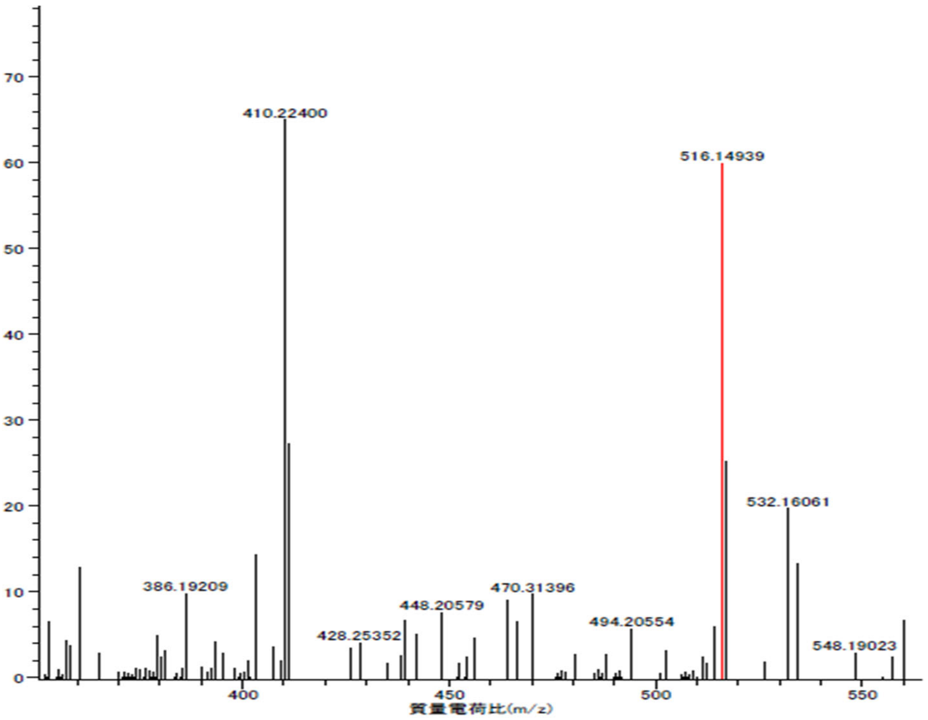


Supplementary Figure S68. ESI-HRMS spectrum of compound 4d

# 12- Compound 4e

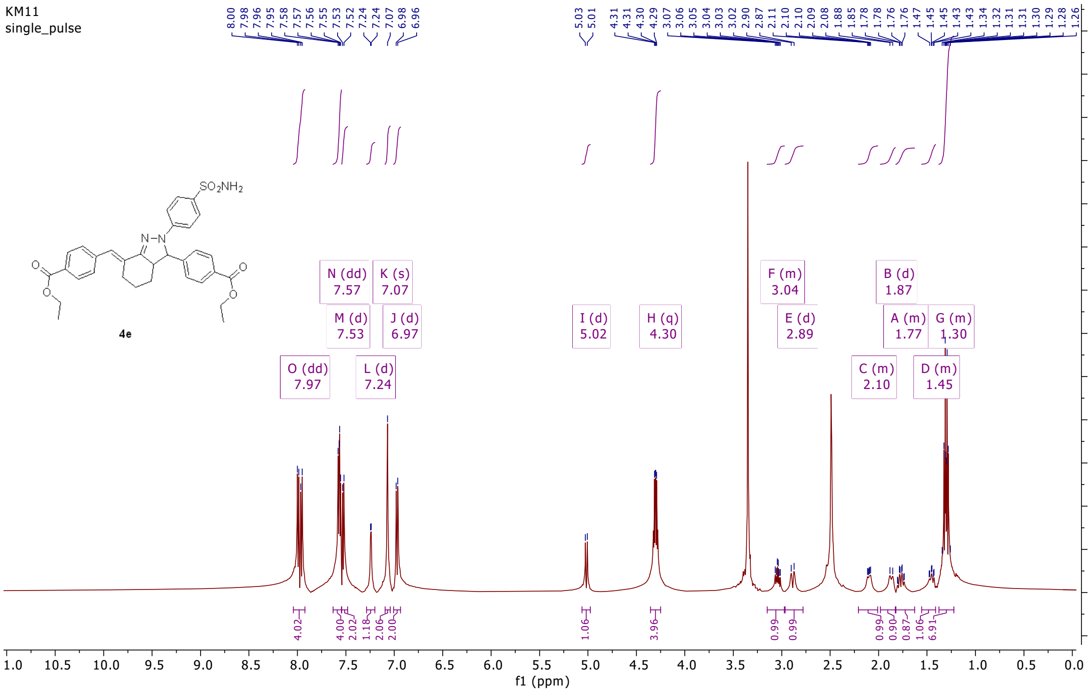


$^1$H NMR (600 MHz, )δ 7.97 (dd, *J* = 20.6, 8.6 Hz, 4H), 7.57 (dd, *J* = 9.0, 6.5 Hz, 4H), 7.53 (d, *J* = 8.6 Hz, 2H), 7.24 (d, *J* = 3.2 Hz, 1H), 7.07 (s, 2H), 6.97 (d, *J* = 9.6 Hz, 2H), 5.02 (d, *J* = 11.9 Hz, 1H), 4.30 (q, *J* = 7.7, 4.7 Hz, 4H), 3.15 – 2.97 (m, *J* = 12.3, 6.1 Hz, 1H), 2.89 (d, *J* = 17.2 Hz, 1H), 2.21 – 2.01 (m, 1H), 1.87 (d, *J* = 16.5 Hz, 1H), 1.82 – 1.63 (m, *J* = 25.8, 12.8, 2.9 Hz, 1H), 1.56 – 1.41 (m, 1H), 1.38 – 1.22 (m, 7H).

## Supplementary Figure S69. $^1$H NMR spectrum of compound 4e

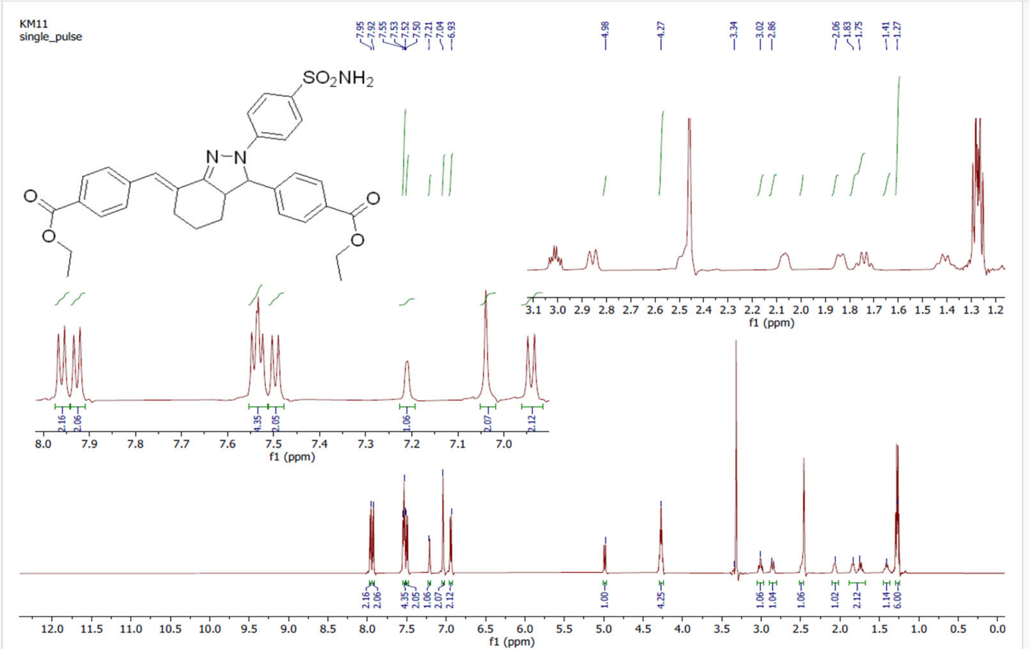


## Supplementary Figure S70. Expanded $^1$H NMR spectrum of compound 4e

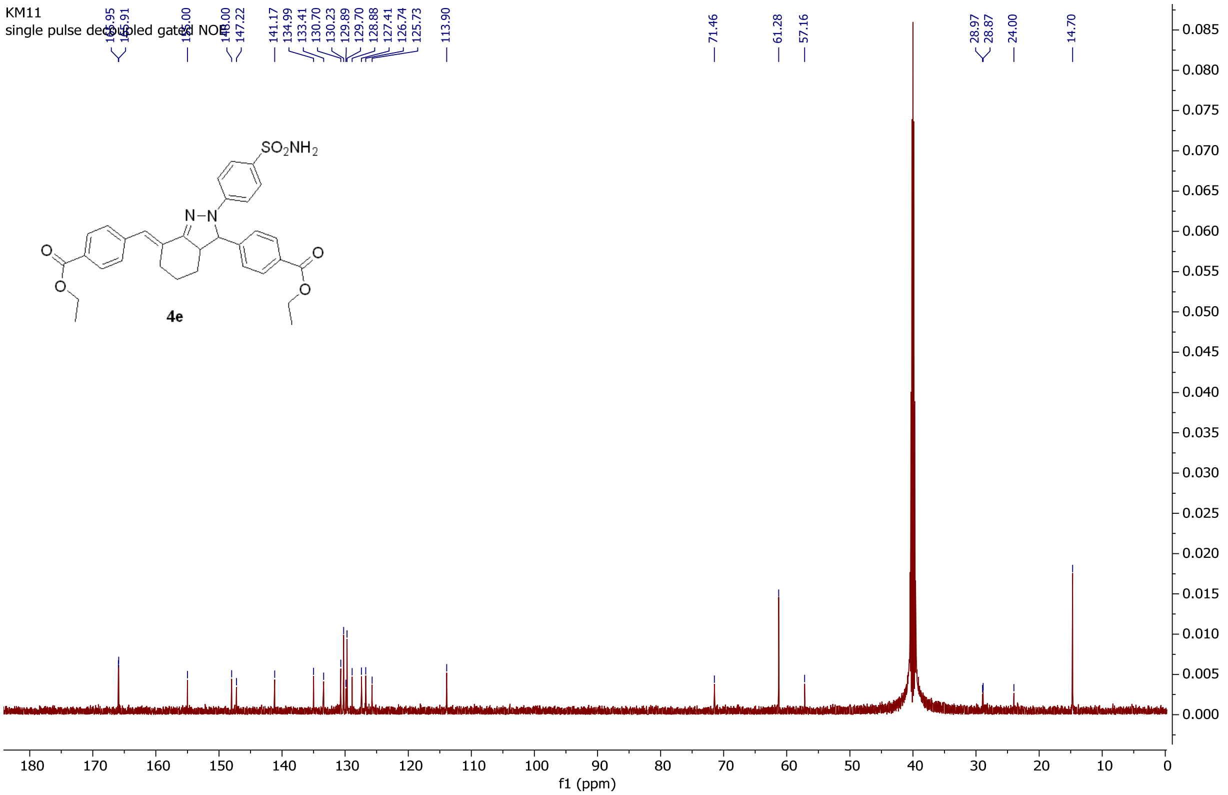


**Supplementary Figure S71. $^{13}$C NMR spectrum of compound 4e**

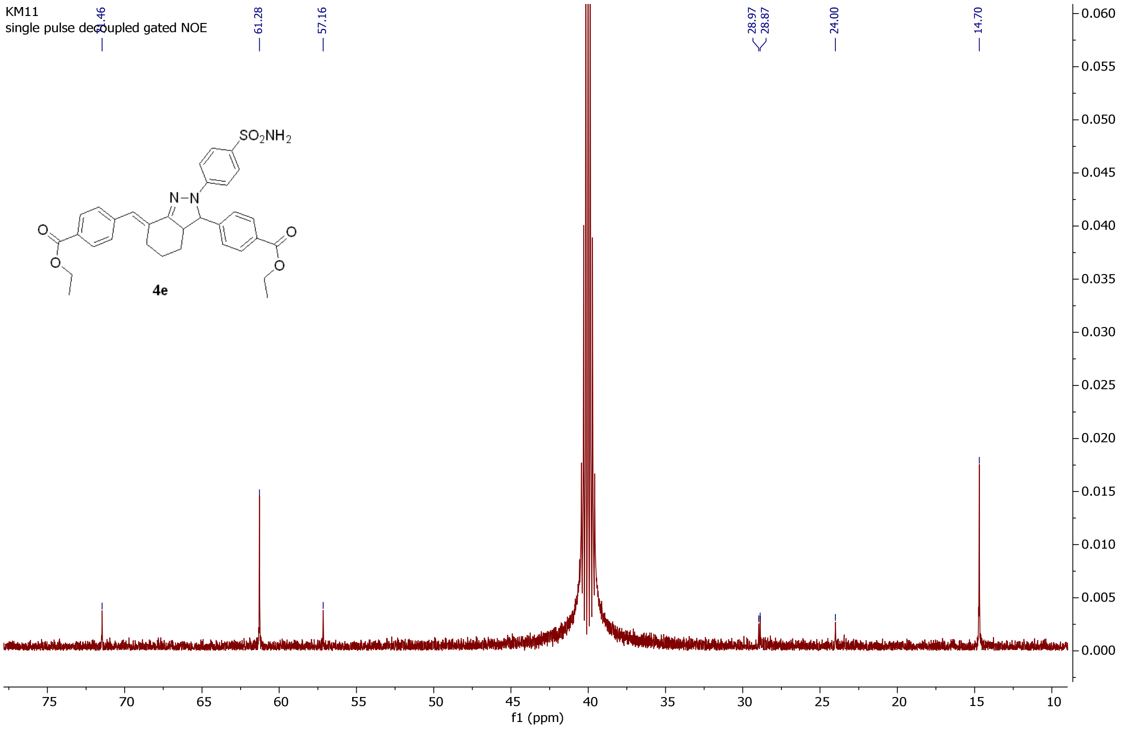


**Supplementary Figure S72. Expanded $^{13}$C NMR spectrum of compound 4e**

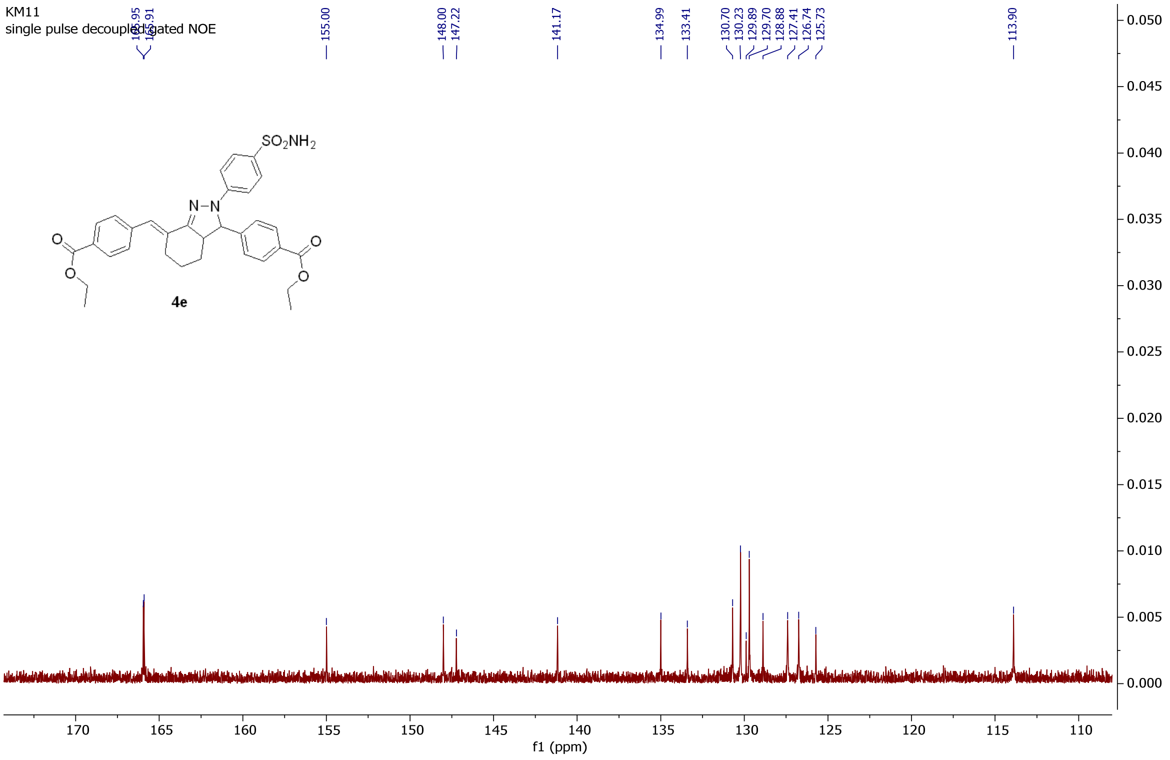


# Supplementary Figure S73. Expanded $^{13}C$ NMR spectrum of compound 4e

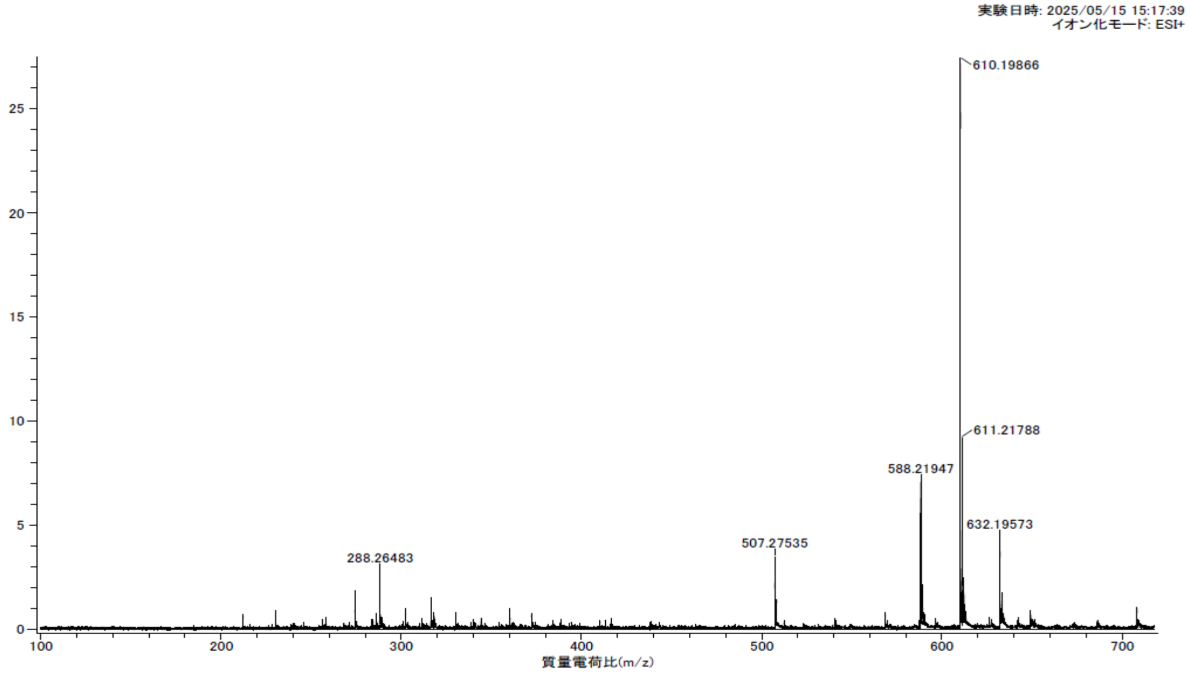


# Supplementary Figure S74. ESI-HRMS spectrum of compound 4e

# Supplementary S1

## Experimental

### General details

All chemicals used for the preparation of the target compounds are of analytical grade and used without further purification. Solvents were purified and freshly distilled before use according to the standard procedures. Melting points were determined using ATM-02 apparatus (AS ONE, Tokyo, Japan) and were uncorrected. $^{1}H$ NMR (600 MHz) and $^{13}C$ NMR (151 MHz) spectra were run on JNM-ECZ 600R/S1 MHz spectrometer, Faculty of Engineering, Yamagata University, Yonezawa, Japan. TMS was used as an internal standard and $CDCl_3$ or DMSO-$d_6$ as solvents. Chemical shift (δ) values are expressed in parts per million (ppm). Coupling constant (*J*) is measured in hertz (Hz). Multiplicity was designated as: s, singlet; d, doublet; t, triplet; q, quartet; p, pentet; dd, doublet of doublet; and m for multiplet. HRMS spectra (ESI-MS) were collected using an AccuTOF JMS-T100LC (JEOL, Tokyo, Japan) in Faculty of Engineering, Yamagata University, Yonezawa, Japan.

**Biology**

**Modulation of TNFα, LDH, and CK-MB**

**Materials and Methods**

Elabscience® Mouse D-LDH (D-Lactate Dehydrogenase) ELISA Kit, Catalog No:E-EL-M0419.

Elabscience® Mouse CKMB (Creatine Kinase MB Isoenzyme) ELISA Kit, Catalog No:E-EL-M0355.

Elabscience® Mouse TNF-α (Tumor Necrosis Factor Alpha) ELISA Kit, Catalog No: E-EL-M3063.

Systemic inflammation will be induced using Lipopolysaccharide, a bacterial endotoxin derived from the outer membrane of Gram-negative bacteria. LPS is widely used in experimental models to mimic acute inflammatory responses through activation of the innate immune system and stimulation of pro-inflammatory cytokine release (Copeland et al., 2005).

LPS will be dissolved in sterile physiological saline immediately before administration. Mice will receive a single intraperitoneal (IP) injection of LPS at a dose of 5 mg/kg body weight, which has been reported to produce a reliable systemic inflammatory response characterized by elevated cytokine production and tissue injury markers (Rittirsch et al., 2008).

**Chemicals and Reagents**

LPS, ce/lecoxib, and all analytical grade reagents used in the present study were obtained from (Sigma aldrich). Celecoxib was used as a standard anti-inflammatory reference drug.

Commercial enzyme-linked immunosorbent assay (ELISA) kits were used for the quantitative determination of lactate dehydrogenase (LDH), tumor necrosis factor-alpha (TNF-α) and creatine kinase-MB (CK-MB) levels in mouse serum according to the manufacturers' protocols.

**Experimental Animals**

Thirty female Swiss albino mice weighing (25g) were purchased from the Animal Care Unit of Alexandria University Medical Research Institute (Alexandria, Egypt). Mice were housed in polyethylene cages under controlled laboratory conditions (22 C ± 3 C temperature, constant relative humidity 40-60% and normal 12/12h dark/light cycle). Food and water were provided *ad libitum*. Before starting the experiment, the animals were allowed to acclimatize to laboratory conditions for **one week** to minimize stress-related physiological variations. All experimental procedures involving animals were conducted in accordance with the internationally accepted guidelines for the care and use of laboratory animals.

**Experimental Design**

A total of **30 mice** were randomly divided into **six experimental groups**, each consisting of **six animals (n = 5)**.

The experimental groups were arranged as follows:

Group I: Normal Control

Animals will receive sterile saline intraperitoneally and will serve as the negative control group.

Group II: LPS Positive Control

Animals will receive sterile saline followed by intraperitoneal injection of LPS (5 mg/kg) to induce systemic inflammation.

**Group III – Celecoxib group**

Animals received celecoxib at a dose of **20 mg/kg body weight** orally prior to LPS administration.

**Group IV – KM3 treatment group**

Animals received the test compound **KM3 (20 mg/kg, orally)** before LPS administration.

**Group V – KM6 treatment group**

Animals received **KM6 (20 mg/kg, orally)** before LPS administration.

**Group VI – KM12 treatment group**

Animals received **KM12 (20 mg/kg, orally)** prior to LPS administration.

All treatments were administered using an oral gavage needle.

**Drug Administration Protocol**

The investigated compounds will be administered orally via gastric gavage once daily for seven consecutive days. On the final experimental day, mice will receive the last dose of the tested compounds one hour prior to LPS injection to evaluate the potential protective effect of the compounds against LPS-induced inflammatory injury. This pretreatment strategy is widely used to assess the anti-inflammatory efficacy of novel therapeutic agents in experimental inflammation models (Copeland et al., 2005).

Sample Collection

Six hours following LPS administration, mice will be lightly anesthetized using thiopental anesthesia. Blood samples will be collected via cardiac puncture using sterile capillary tubes. The collected blood will be allowed to clot at room temperature for approximately 15 minutes, followed

by centrifugation at 3000 rpm for 10 minutes to separate the serum. Serum samples will then be carefully collected and stored at −80°C until biochemical analysis.

Determination of Serum TNF-α Levels

Serum levels of tumor necrosis factor-alpha (TNF-α) were measured using a commercially available enzyme-linked immunosorbent assay (ELISA) kit according to the manufacturer's instructions.

Briefly:

1. Serum samples and standards were added to the antibody-coated microplate wells.
2. The plate was incubated to allow binding between TNF-α and the immobilized antibodies.
3. After incubation, wells were washed multiple times to remove unbound components.
4. A specific enzyme-linked secondary antibody was added.
5. Following further incubation and washing steps, a chromogenic substrate solution was added.
6. The enzymatic reaction produced a color change proportional to the concentration of TNF-α present in the samples.

The absorbance was measured at 450 nm using a microplate reader, and TNF-α concentrations were calculated using a standard calibration curve.

**Determination of Serum LDH Activity**

Serum lactate dehydrogenase (LDH) activity was measured using a commercially available colorimetric assay kit.

The assay is based on the enzymatic conversion of lactate to pyruvate in the presence of $NAD^+$, resulting in the formation of NADH. The rate of NADH formation is proportional to LDH activity in the sample.

The reaction mixture containing serum sample and assay reagents was incubated under controlled conditions, and the absorbance was measured spectrophotometrically according to the manufacturer's instructions.

LDH activity was expressed in **U/L**.

**Determination of Serum CK-MB Levels**

Serum creatine kinase-MB (CK-MB) levels were determined using a commercial ELISA kit designed for quantitative measurement of CK-MB.

The assay was carried out according to the manufacturer's protocol and involved the following steps:

1. Addition of serum samples and standards into antibody-coated wells.
2. Incubation to allow antigen-antibody binding.
3. Washing steps to remove unbound substances.
4. Addition of enzyme-conjugated detection antibodies.
5. Development of color after addition of substrate solution.

The optical density was measured at 450 nm, and CK-MB concentrations were calculated using a standard curve.

Statistical Analysis

All experimental data were expressed as mean ± standard deviation (SD).

Statistical analysis was performed using GraphPad Prism software.

Differences between experimental groups were analyzed using one-way analysis of variance (ANOVA) followed by Tukey's post hoc multiple comparison test.

A p-value less than 0.05 ($p < 0.05$) was considered statistically significant.

References

1. Copeland S., Warren H., Lowry S., Calvano S., Remick D. Acute inflammatory response to endotoxin in mice and humans. *Clinical and Diagnostic Laboratory Immunology*. 2005.
2. Rittirsch D., Flierl M., Ward P. Harmful molecular mechanisms in sepsis. *Nature Reviews Immunology*. 2008

# Enzymatic Methodology

## Cox-1 and Cox-2

### Materials and reagents

The COX-1 Colorimetric Inhibitor Screening Assay Kit (Cayman Chemical, Cat. 701050) was used to evaluate potential cyclooxygenase-1 (COX-1) inhibitors. The kit provides human recombinant COX-1 enzyme, COX-2 enzyme, assay buffer, hemin, arachidonic acid substrate, potassium hydroxide, colorimetric reagent (TMPD), 96-well plates and covers. Kit components were stored and handled according to the manufacturer's recommendations

### Reagent preparation

- The 10× assay buffer was diluted to working concentration with ultrapure water.
- Hemin (300 µL in DMSO) was diluted by mixing 88 µL with ~1.912 mL of diluted buffer; this solution was stable for up to 12 h at ambient temperature
- The COX-1 enzyme was thawed on ice and diluted by combining 120 µL enzyme with 360 µL diluted assay buffer; this dilution allowed approximately 48 wells per preparation and remained stable on ice for about one hour

### Substrate preparation

Arachidonic acid solution was prepared by mixing 100 µL of the supplied ethanol solution with 100 µL potassium hydroxide, vortexing, and diluting with 1.8 mL HPLC-grade water to yield ~1.1 mM final concentration. Prepared substrate was used within 30 minutes; addition of 20 µL per well yielded a final substrate concentration of ~100 µM. Adjust concentration as required

### Assay protocol

Inhibitor test compounds were dissolved in ethanol or water and dispensed into designated wells. Pre-aliquoted enzyme mixture (COX-1 diluted) and hemin were added to each well and mixed gently. Following a brief incubation, arachidonic acid substrate was added to initiate the reaction. The peroxidase activity of COX was monitored continuously by measuring the oxidation of TMPD at 590 nm using a microplate reader. The rate of color development is inversely proportional to inhibitor potency. Appropriate controls—including vehicle-only, enzyme-only, and positive control inhibitors (e.g., NSAIDs)—were included on each plate.

### Data analysis

Absorbance at 590 nm was recorded at regular intervals. Reaction rates were calculated by linear regression of absorbance values over the initial linear phase. Percent inhibition at each compound concentration was determined relative to vehicle control. $IC_{50}$ values were calculated by non-linear regression fitting of inhibition versus concentration curves using software such as

GraphPad Prism. All experiments were performed in triplicate, and data are presented as mean ± standard deviation.

### Quality control and validation

The kit's dual-enzyme format (COX-1 and COX-2) enables screening for isozyme specificity. Inhibition profiles obtained with reference compounds (celecoxib and indomethacin) exhibit potency patterns consistent with previously reported $IC_{50}$ values, validating assay performance

## 5-Lipoxygenase

### Materials and Reagents

The **5-Lipoxygenase Colorimetric Inhibitor Screening Assay Kit** (Cayman Chemical, Cat. 760700) was employed to evaluate inhibitory potency against lipoxygenase (LO) enzymes. The kit includes a purified soybean-derived 15-LO enzyme (usable as proxy for 5-LO or other isoforms), 10× Assay Buffer, Developing Reagents 1 and 2, substrates (arachidonic and linoleic acids), potassium hydroxide solution, Zileuton as positive control, colorimetric 96-well plate, and covers

### Reagent Preparation

- **Assay Buffer (1×)**: Dilute 3 mL of 10× concentrate with 27 mL ultrapure water to prepare working buffer; store at 4 °C (stable ≥2 months)
- **Chromogen**: Mix equal volumes of Developing Reagent 1 and Developing Reagent 2, vortex gently; allocate ~100–120 µL per well and use within 1 hour
- **Enzyme stock**: Dilute 15-LO by adding 10 µL enzyme to 990 µL 1× buffer (1:100). For full-plate screening, prepare 110 µL enzyme into 10.89 mL buffer. Keep enzyme on ice; use within ~60 minutes
- **Substrate solutions** (arachidonic or linoleic acid): Transfer 25 µL substrate to new vial, add 25 µL 0.1 M KOH, vortex, dilute with 950 µL water to ~1 mM working stock; use within 30 minutes. Addition of 10 µL per well yields ~91 µM final concentration
- **Positive control inhibitor (**Zileuton**)**: Dissolve 550 nmol vial in 500 µL buffer to obtain 1.1 mM stock; adding 10 µL to assay gives 100 µM final inhibitor concentration Test compounds should be dissolved in methanol or DMSO (DMF and ethanol not recommended) and diluted into 1× buffer to create an 11× stock relative to final well concentration; vehicle control wells with matching solvent must be included Plate Setup and Controls

- At minimum, include two blank wells (buffer only), two positive-control wells (enzyme + Zileuton), duplicate or triplicate wells for enzyme-only (100% activity), and wells containing serial dilutions of test inhibitors.
- All assays are performed at ambient temperature with gentle orbital shaking

## Assay Procedure

1. **Blank wells**: Add 100 µL 1× buffer.
2. **Activity control wells**: Add 90 µL enzyme dilution plus 10 µL solvent.
3. **Negative (no enzyme) controls**: Add buffer only.
4. **Inhibitor wells**: Add 90 µL enzyme dilution and 10 µL of test compound or Zileuton.
5. **Initiate reaction**: Add substrate (10 µL) to all wells.
6. Incubate for ~10 minutes with shaking, then add Chromogen (100–120 µL) to each well.
7. Incubate further for 5 minutes with shaking.
8. Remove cover and measure absorbance at **490 nm** using microplate reader Total assay volume per well should be ~210 µL. Ensure blank absorbance is < 0.22 AU; if higher, dilute buffer or medium appropriately

### Data Analysis

- Subtract blank absorbance from all experimental wells to obtain corrected readings.
- Express residual enzyme activity relative to activity-control wells (100%).
- Plot percent inhibition versus compound concentration and fit data via non-linear regression (e.g. four-parameter logistic) to determine $IC_{50}$ values.
- Perform assays in at least duplicate (triplicate preferred) and report results as mean ± standard deviation

## Quality Control and Validation

- **Specificity**: Although the supplied enzyme is soybean 15-LO, researchers may substitute purified human 5-LO or 12-LO isoforms while using the kit buffer and chromogenic reagents under the same protocol for isoform-specific screening
- **Interference checks**: Test compounds should be screened for interference by including compounds + chromogen + blank (no enzyme), especially if they may absorb or react chemically. Significant absorbance (> 0.2 AU) may require dilution or exclusion

## Soluble Epoxide Hydrolase (sEH) Inhibitory Activity

The inhibitory activity of the synthesized compounds against human recombinant soluble epoxide hydrolase (sEH) was evaluated using a fluorescence-based Soluble Epoxide Hydrolase Inhibitor Screening Assay Kit (Cayman Chemical, Ann Arbor, MI, USA; Item No. 10011671), according to the manufacturer's protocol with slight optimization for dose–response analysis.

**Assay Principle**

This assay is based on the hydrolysis of the fluorogenic substrate PHOME [(3-phenyl-oxiranyl)-acetic acid cyano-(6-methoxy-naphthalen-2-yl)-methyl ester] by recombinant human sEH. Upon enzymatic hydrolysis of the epoxide moiety, an intramolecular cyclization reaction occurs under basic conditions, resulting in the release of an unstable cyanohydrin intermediate that rapidly decomposes to generate 6-methoxy-2-naphthaldehyde, a highly fluorescent product. The fluorescence intensity directly reflects sEH catalytic activity and is inversely proportional to inhibitor potency. Fluorescence was measured at an excitation wavelength of 330 nm and an emission wavelength of 465 nm using an Agilent BioTek Synergy™ H1 Hybrid Multi-Mode Microplate Reader.

**Reagents and Buffer Preparation**

The assay kit provided the following components:

- 10× sEH assay buffer
- human recombinant sEH enzyme
- PHOME substrate
- AUDA inhibitor control
- assay calibrator
- black 96-well microplate

The 10× assay buffer was diluted to 1× working buffer by adding 27 mL of ultrapure water to 3 mL of stock buffer, yielding a final volume of 30 mL.

For enzyme preparation, recombinant human sEH was thawed on ice and diluted 1:200 in 1× assay buffer immediately before use to preserve catalytic activity.

The PHOME substrate was also diluted 1:200 in 1× assay buffer and equilibrated to room temperature for 30 min before initiating the reaction.

The supplied reference inhibitor AUDA was diluted in DMSO to the required working concentration and used as the positive inhibitory control.

**Preparation of Test Compounds**

The synthesized compounds were dissolved in dimethyl sulfoxide (DMSO) to prepare concentrated stock solutions. Serial dilutions were then freshly prepared in 1× assay buffer to achieve 40× the desired final in-well concentration.

A broad concentration range was selected for each compound to allow accurate determination of the half-maximal inhibitory concentration (IC50) using nonlinear regression analysis.

The final solvent concentration was kept constant in all wells, and corresponding vehicle control wells containing the same DMSO concentration were included to exclude solvent-related interference.

**Fluorometric sEH Inhibition Assay Procedure**

The assay was carried out in black 96-well solid microplates in a final reaction volume of 200 µL per well, with all samples analyzed in duplicate.

The plate layout included:

- background wells
- vehicle control wells
- test compound wells
- inhibitor control wells (AUDA)

For each test well, the following were sequentially added:

- 95 µL of 1× sEH assay buffer
- 5 µL of test compound solution
- 50 µL of diluted recombinant human sEH enzyme

For vehicle control wells, the test compound was replaced with 5 µL DMSO vehicle.

For background wells, enzyme was omitted and replaced with assay buffer to correct for non-enzymatic fluorescence.

For positive control wells, 5 µL of diluted AUDA inhibitor was added instead of the test compound.

After reagent addition, the plate was gently mixed by pipetting to ensure homogeneity while avoiding bubble formation, then covered with foil and incubated at room temperature for 15 min to allow enzyme–inhibitor preincubation.

The enzymatic reaction was initiated by adding: 50 µL of diluted PHOME substrate to all wells. The plate was incubated for 30 min at room temperature in endpoint mode, protected from light.

**Fluorescence Measurement**

At the end of incubation, fluorescence intensity was measured using the microplate reader at: $\lambda$Ex = 330 nm, $\lambda$Em = 465 nm. The fluorescence output was recorded as Relative Fluorescence Units (RFU). For kinetic validation experiments, fluorescence can alternatively be monitored every minute for 30 min, and the initial linear reaction velocity (RFU/min) can be used for analysis.

**Calculation of Enzyme Inhibition**

The mean fluorescence values of duplicate wells were calculated first. Background fluorescence was subtracted from all corresponding readings to obtain the corrected RFU values. The percentage of remaining sEH activity was calculated using the following equation: %Remaining Activity= Corrected RFU of test compound / Corrected RFU of vehicle control ×100

The percentage inhibition was then determined as: %Inhibition=100−%Remaining Activity

Dose–response curves were constructed by plotting: log concentration of compound versus % remaining enzyme activity. Using nonlinear regression analysis with a four-parameter logistic (4PL) model. The IC50 value, defined as the concentration required to inhibit 50% of sEH activity, was calculated using GraphPad Prism software.

**Raw Data**

# 5-lipoxigenase activity assay

**Compound 3g**

**$IC_{50}$ = 1.094 µM**

| Concentration (µM) | Reading 1 | Reading 2 | Mean Absorbance |
|---|---|---|---|
| 0.1 | 0.7273 | 0.7261 | 0.7267 |
| 0.2 | 0.6746 | 0.6758 | 0.6752 |
| 0.4 | 0.5971 | 0.5964 | 0.5968 |
| 1 | 0.4529 | 0.4535 | 0.4532 |
| 2 | 0.3227 | 0.3214 | 0.3221 |
| 5 | 0.2159 | 0.2163 | 0.2161 |
| 10 | 0.1485 | 0.1497 | 0.1491 |
| 15 | 0.1294 | 0.1281 | 0.1288 |

| Concentration (µM) | Mean Absorbance | % Inhibition |
|---|---|---|
| 0.1 | 0.7267 | 11.2435 |
| 0.2 | 0.6752 | 17.5566 |
| 0.4 | 0.5968 | 27.0799 |
| 1 | 0.4532 | 44.6541 |
| 2 | 0.3221 | 60.6577 |
| 5 | 0.2161 | 73.6107 |
| 10 | 0.1491 | 81.7932 |
| 15 | 0.1288 | 86.1840 |

| Log[Conc.] (log10 µM) | % Inhibition |
|---|---|
| -1.000 | 11.2435 |
| -0.698 | 17.5566 |
| -0.398 | 27.0799 |
| 0.000 | 44.6541 |
| 0.301 | 60.6577 |
| 0.699 | 73.6107 |
| 1.000 | 81.7932 |
| 1.176 | 86.1840 |

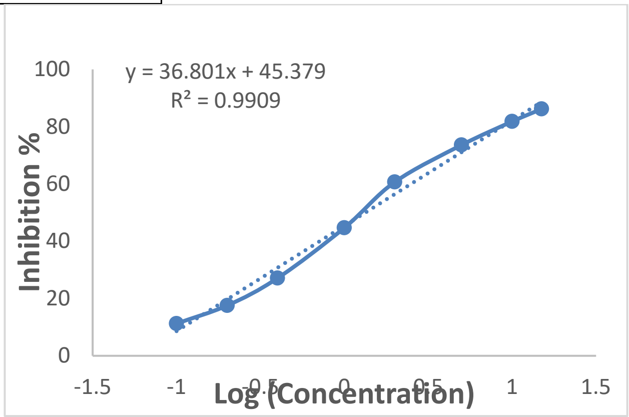

**Compound 4b**
**$IC_{50}$ = 3.077 µM**

| Concentration (µM) | Reading 1 | Reading 2 | Mean Absorbance |
|---|---|---|---|
| 0.1 | 0.7746 | 0.7732 | 0.7739 |
| 0.2 | 0.7625 | 0.7637 | 0.7631 |
| 0.4 | 0.7421 | 0.7406 | 0.7414 |
| 1 | 0.6984 | 0.6995 | 0.6989 |
| 2 | 0.6237 | 0.6249 | 0.6243 |
| 5 | 0.4915 | 0.4897 | 0.4906 |
| 10 | 0.3981 | 0.3994 | 0.3987 |
| 15 | 0.3542 | 0.3526 | 0.3534 |

| Concentration (µM) | Mean Absorbance | % Inhibition |
|---|---|---|
| 0.1 | 0.7739 | 5.3811 |
| 0.2 | 0.7631 | 6.7394 |
| 0.4 | 0.7414 | 9.3747 |
| 1 | 0.6989 | 14.5624 |
| 2 | 0.6243 | 23.6845 |
| 5 | 0.4906 | 40.0048 |
| 10 | 0.3987 | 51.2679 |
| 15 | 0.3534 | 71.2943 |

| Log[Conc.] (log10 µM) | % Inhibition |
|---|---|
| -1.000 | 5.3811 |
| -0.698 | 6.7394 |
| -0.398 | 9.3747 |
| 0.000 | 14.5624 |
| 0.301 | 23.6845 |
| 0.699 | 40.0048 |
| 1.000 | 51.2679 |
| 1.176 | 71.2943 |

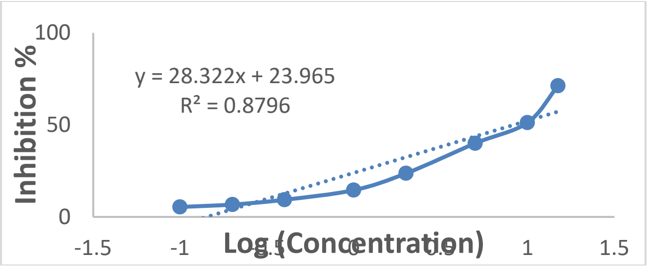


**Compound 3f**

**$IC_{50}$ =** 0.927 **µM**

| **Concentration (µM)** | **Reading 1** | **Reading 2** | **Mean Absorbance** |
|---|---|---|---|
| 0.1 | 0.7238 | 0.7225 | 0.7231 |
| 0.2 | 0.6937 | 0.6949 | 0.6943 |
| 0.4 | 0.6154 | 0.6167 | 0.6160 |
| 1 | 0.4385 | 0.4373 | 0.4379 |
| 2 | 0.2918 | 0.2929 | 0.2924 |
| 5 | 0.1697 | 0.1682 | 0.1690 |
| 10 | 0.1091 | 0.1103 | 0.1097 |
| 15 | 0.0937 | 0.0924 | 0.0930 |

| **Concentration (µM)** | **Mean Absorbance** | **% Inhibition** |
|---|---|---|
| 0.1 | 0.7231 | 12.42191 |
| 0.2 | 0.6943 | 15.8247 |
| 0.4 | 0.6160 | 25.3374 |
| 1 | 0.4379 | 46.9290 |
| 2 | 0.2924 | 64.5619 |
| 5 | 0.1690 | 79.5159 |
| 10 | 0.1097 | 86.7070 |
| 15 | 0.0930 | 88.3955 |

| **Log[Conc.] (log10 µM)** | **% Inhibition** |
|---|---|
| -1.000 | 12.42191 |
| -0.698 | 15.8247 |
| -0.398 | 25.3374 |
| 0.000 | 46.9290 |
| 0.301 | 64.5619 |
| 0.699 | 79.5159 |
| 1.000 | 86.7070 |

| Log[Conc.] (log10 µM) | % Inhibition |
|---|---|
| 1.176 | 88.3955 |

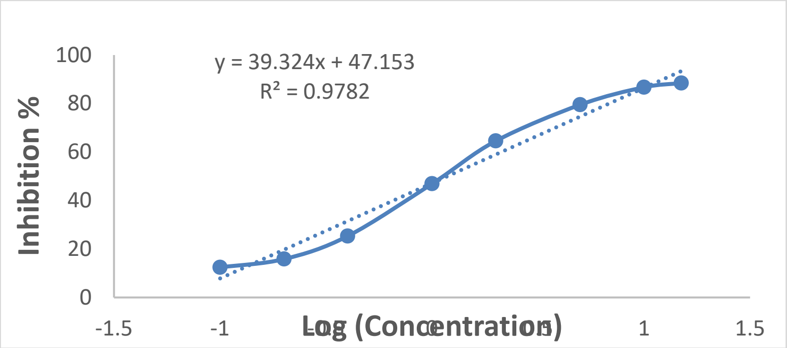


**Compound 4a**

**$IC_{50}$ = 6.469 µM**

| Concentration (µM) | Reading 1 | Reading 2 | Mean Absorbance |
|---|---|---|---|
| 0.1 | 0.8033 | 0.8021 | 0.8027 |
| 0.2 | 0.7875 | 0.7887 | 0.7881 |
| 0.4 | 0.7696 | 0.7709 | 0.7703 |
| 1 | 0.7268 | 0.7255 | 0.7261 |
| 2 | 0.6829 | 0.6813 | 0.6821 |
| 5 | 0.5617 | 0.5629 | 0.5623 |
| 10 | 0.4125 | 0.4138 | 0.4132 |
| 15 | 0.3297 | 0.3309 | 0.3303 |

| Concentration (µM) | Mean Absorbance | % Inhibition |
|---|---|---|
| 0.1 | 0.8027 | 3.5992 |
| 0.2 | 0.7881 | 5.5693 |
| 0.4 | 0.7703 | 7.7118 |
| 1 | 0.7261 | 13.0114 |
| 2 | 0.6821 | 18.2677 |
| 5 | 0.5623 | 32.6472 |
| 10 | 0.4132 | 50.5023 |
| 15 | 0.3303 | 60.4882 |

| Log[Conc.] (log10 µM) | % Inhibition |
|---|---|
| -1.000 | 3.5992 |
| -0.698 | 5.5693 |
| -0.398 | 7.7118 |
| 0.000 | 13.0114 |
| 0.301 | 18.2677 |
| 0.699 | 32.6472 |
| 1.000 | 50.5023 |
| 1.176 | 60.4882 |

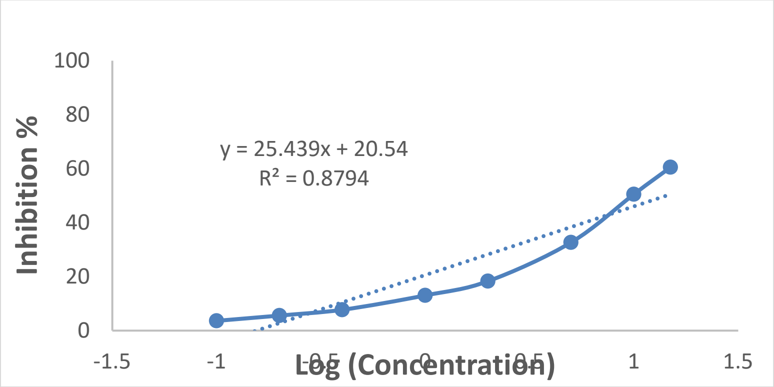


**Compound 4c**

**$IC_{50}$ =** 1.681 **µM**

| Concentration (µM) | Reading 1 | Reading 2 | Mean Absorbance |
|---|---|---|---|
| 0.1 | 0.7705 | 0.7691 | 0.7698 |
| 0.2 | 0.7324 | 0.7336 | 0.7330 |
| 0.4 | 0.6747 | 0.6759 | 0.6753 |
| 1 | 0.5081 | 0.5073 | 0.5077 |
| 2 | 0.3895 | 0.3881 | 0.3888 |
| 5 | 0.2462 | 0.2475 | 0.2469 |
| 10 | 0.1694 | 0.1708 | 0.1701 |
| 15 | 0.1559 | 0.1571 | 0.1565 |

| Concentration (µM) | Mean Absorbance | % Inhibition |
|---|---|---|
| 0.1 | 0.7698 | 9.0443 |
| 0.2 | 0.7330 | 13.4484 |
| 0.4 | 0.6753 | 20.2643 |
| 1 | 0.5077 | 40.0418 |
| 2 | 0.3888 | 54.0727 |
| 5 | 0.2469 | 70.8375 |

| Concentration (µM) | Mean Absorbance | % Inhibition |
|---|---|---|
| 10 | 0.1701 | 79.9020 |
| 15 | 0.1565 | 81.6328 |

| Log[Conc.] (log10 µM) | % Inhibition |
|---|---|
| -1.000 | 9.0443 |
| -0.698 | 13.4484 |
| -0.398 | 20.2643 |
| 0.000 | 40.0418 |
| 0.301 | 54.0727 |
| 0.699 | 70.8375 |
| 1.000 | 79.9020 |
| 1.176 | 81.6328 |

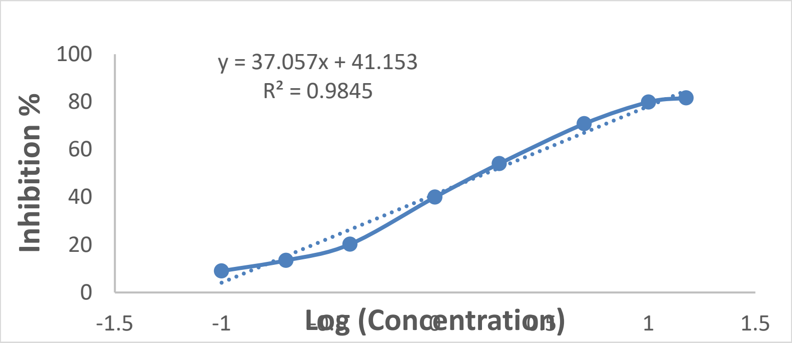


**Compound 3a**

**$IC_{50}$ = 0.802 µM**

| Concentration (µM) | Reading 1 | Reading 2 | Mean Absorbance |
|---|---|---|---|
| 0.1 | 0.7451 | 0.7439 | 0.7445 |
| 0.2 | 0.7113 | 0.7097 | 0.7105 |
| 0.4 | 0.6338 | 0.6351 | 0.6345 |
| 1 | 0.4587 | 0.4574 | 0.4580 |
| 2 | 0.3212 | 0.3199 | 0.3206 |
| 5 | 0.1946 | 0.1958 | 0.1952 |
| 10 | 0.1069 | 0.1083 | 0.1076 |
| 15 | 0.0629 | 0.0638 | 0.0634 |

| Concentration (µM) | Mean Absorbance | % Inhibition |
|---|---|---|
| 0.1 | 0.7445 | 13.1563 |
| 0.2 | 0.7105 | 17.2984 |
| 0.4 | 0.6345 | 26.1789 |
| 1 | 0.4580 | 46.7146 |
| 2 | 0.3206 | 62.6926 |
| 5 | 0.1952 | 77.2939 |
| 10 | 0.1076 | 87.4817 |
| 15 | 0.0634 | 92.7088 |

| Log[Conc.] (log10 µM) | % Inhibition |
|---|---|
| -1.000 | 13.1563 |
| -0.698 | 17.2984 |
| -0.398 | 26.1789 |
| 0.000 | 46.7146 |
| 0.301 | 62.6926 |
| 0.699 | 77.2939 |
| 1.000 | 87.4817 |
| 1.176 | 92.7088 |

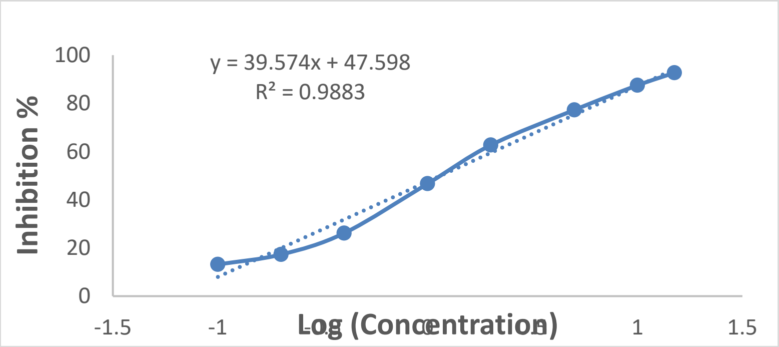


**Compound 4d**

**$IC_{50}$ = 5.907 µM**

| Concentration (µM) | Reading 1 | Reading 2 | Mean Absorbance |
|---|---|---|---|
| 0.1 | 0.8396 | 0.8382 | 0.8389 |
| 0.2 | 0.8181 | 0.8174 | 0.8178 |
| 0.4 | 0.7935 | 0.7929 | 0.7932 |
| 1 | 0.7463 | 0.7447 | 0.7455 |
| 2 | 0.6832 | 0.6816 | 0.6824 |
| 5 | 0.5633 | 0.5619 | 0.5626 |

| Concentration (µM) | Reading 1 | Reading 2 | Mean Absorbance |
|---|---|---|---|
| 10 | 0.4681 | 0.4673 | 0.4677 |
| 15 | 0.2724 | 0.2735 | 0.2729 |

| Concentration (µM) | Mean Absorbance | % Inhibition |
|---|---|---|
| 0.1 | 0.8389 | 3.8552 |
| 0.2 | 0.8178 | 6.2225 |
| 0.4 | 0.7932 | 9.0436 |
| 1 | 0.7455 | 14.5379 |
| 2 | 0.6824 | 21.7555 |
| 5 | 0.5626 | 35.4976 |
| 10 | 0.4677 | 46.3768 |
| 15 | 0.2729 | 68.9554 |

| Log[Conc.] (log10 µM) | % Inhibition |
|---|---|
| -1.000 | 3.8552 |
| -0.698 | 6.2225 |
| -0.398 | 9.0436 |
| 0.000 | 14.5379 |
| 0.301 | 21.7555 |
| 0.699 | 35.4976 |
| 1.000 | 46.3768 |
| 1.176 | 68.9554 |

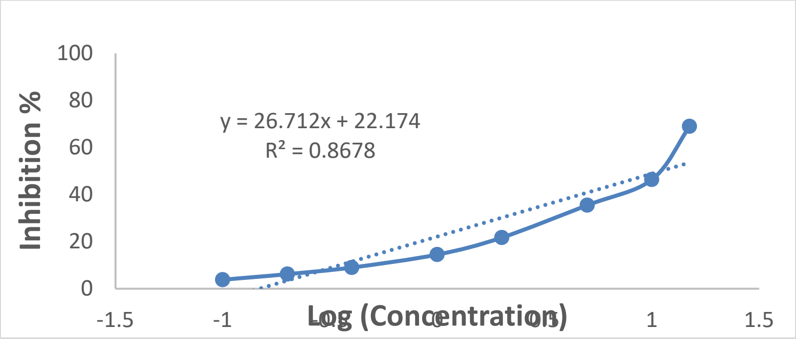

**Compound 4e**
**$IC_{50}$ = 2.871 µM**

| Concentration (µM) | Reading 1 | Reading 2 | Mean Absorbance |
|---|---|---|---|
| 0.1 | 0.8302 | 0.8315 | 0.8309 |
| 0.2 | 0.8114 | 0.8126 | 0.8120 |
| 0.4 | 0.7793 | 0.7809 | 0.7801 |
| 1 | 0.7167 | 0.7152 | 0.7159 |
| 2 | 0.6286 | 0.6271 | 0.6279 |
| 5 | 0.5123 | 0.5108 | 0.5115 |
| 10 | 0.3846 | 0.3862 | 0.3854 |
| 15 | 0.2001 | 0.1994 | 0.1997 |

| Concentration (µM) | Mean Absorbance | % Inhibition |
|---|---|---|
| 0.1 | 0.8309 | 7.1184 |
| 0.2 | 0.8120 | 9.2103 |
| 0.4 | 0.7801 | 12.7826 |
| 1 | 0.7159 | 19.9391 |
| 2 | 0.6279 | 29.7989 |
| 5 | 0.5115 | 42.8021 |
| 10 | 0.3854 | 56.9164 |
| 15 | 0.1997 | 77.5883 |

| Log[Conc.] (log10 µM) | % Inhibition |
|---|---|
| -1.000 | 7.1184 |
| -0.698 | 9.2103 |
| -0.398 | 12.7826 |
| 0.000 | 19.9391 |
| 0.301 | 29.7989 |
| 0.699 | 42.8021 |
| 1.000 | 56.9164 |
| 1.176 | 77.5883 |

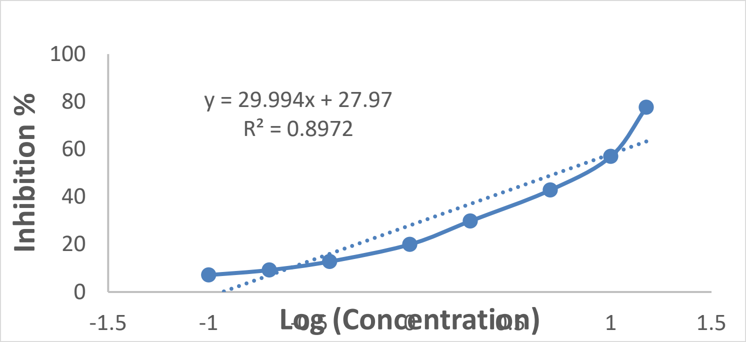


**Compound 3e**

**$IC_{50}$ = 6.182 µM**

| **Concentration (µM)** | **Reading 1** | **Reading 2** | **Mean Absorbance** |
|---|---|---|---|
| 0.1 | 0.8671 | 0.8686 | 0.8678 |
| 0.2 | 0.8494 | 0.8503 | 0.8499 |
| 0.4 | 0.8192 | 0.8181 | 0.8186 |
| 1 | 0.7814 | 0.7827 | 0.7820 |
| 2 | 0.7338 | 0.7349 | 0.7344 |
| 5 | 0.6596 | 0.6583 | 0.6589 |
| 10 | 0.5621 | 0.5613 | 0.5617 |
| 15 | 0.3295 | 0.3309 | 0.3302 |

| **Concentration (µM)** | **Mean Absorbance** | **% Inhibition** |
|---|---|---|
| 0.1 | 0.8678 | 4.3199 |
| 0.2 | 0.8499 | 6.3684 |
| 0.4 | 0.8186 | 9.8161 |
| 1 | 0.7820 | 13.8224 |
| 2 | 0.7344 | 19.0613 |
| 5 | 0.6589 | 27.4021 |
| 10 | 0.5617 | 38.0862 |
| 15 | 0.3302 | 63.7221 |

| **Log[Conc.] (log10 µM)** | **% Inhibition** |
|---|---|
| -1.000 | 4.3199 |
| -0.698 | 6.3684 |
| -0.398 | 9.8161 |
| 0.000 | 13.8224 |
| 0.301 | 19.0613 |
| 0.699 | 27.4021 |

| Log[Conc.] (log10 µM) | % Inhibition |
|---|---|
| 1.000 | 38.0862 |
| 1.176 | 63.7221 |

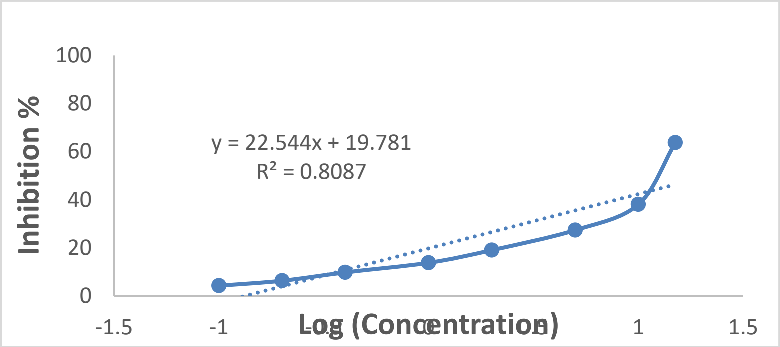


**Compound 3d**

**$IC_{50}$ = 7.336 µM**

| Concentration (µM) | Reading 1 | Reading 2 | Mean Absorbance |
|---|---|---|---|
| 0.1 | 0.8675 | 0.8688 | 0.8682 |
| 0.2 | 0.8549 | 0.8556 | 0.8552 |
| 0.4 | 0.8387 | 0.8374 | 0.8381 |
| 1 | 0.8195 | 0.8181 | 0.8188 |
| 2 | 0.7786 | 0.7772 | 0.7779 |
| 5 | 0.7149 | 0.7134 | 0.7141 |
| 10 | 0.6367 | 0.6381 | 0.6374 |
| 15 | 0.3856 | 0.3869 | 0.3862 |

| Concentration (µM) | Mean Absorbance | % Inhibition |
|---|---|---|
| 0.1 | 0.8682 | 2.8554 |
| 0.2 | 0.8552 | 4.1601 |
| 0.4 | 0.8381 | 6.0713 |
| 1 | 0.8188 | 8.2303 |
| 2 | 0.7779 | 12.8226 |
| 5 | 0.7141 | 20.0022 |
| 10 | 0.6374 | 28.5973 |
| 15 | 0.3862 | 56.7221 |

| Log[Conc.] (log10 µM) | % Inhibition |
|---|---|
| -1.000 | 2.8554 |
| -0.698 | 4.1601 |

| Log[Conc.] (log10 µM) | % Inhibition |
|---|---|
| -0.398 | 6.0713 |
| 0.000 | 8.2303 |
| 0.301 | 12.8226 |
| 0.699 | 20.0022 |
| 1.000 | 28.5973 |
| 1.176 | 56.7221 |

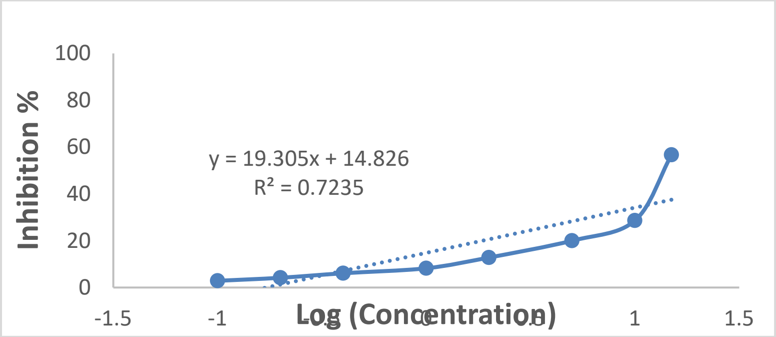


**Compound 3c**

**$IC_{50}$ = 0.874 µM**

| Concentration (µM) | Reading 1 | Reading 2 | Mean Absorbance |
|---|---|---|---|
| 0.1 | 0.7486 | 0.7498 | 0.7492 |
| 0.2 | 0.6952 | 0.6941 | 0.6946 |
| 0.4 | 0.6154 | 0.6172 | 0.6163 |
| 1 | 0.4838 | 0.4859 | 0.4849 |
| 2 | 0.3712 | 0.3724 | 0.3718 |
| 5 | 0.2975 | 0.2989 | 0.2982 |
| 10 | 0.2286 | 0.2298 | 0.2292 |
| 15 | 0.0713 | 0.0731 | 0.0722 |

| Concentration (µM) | Mean Absorbance | % Inhibition |
|---|---|---|
| 0.1 | 0.7492 | 13.0443 |
| 0.2 | 0.6946 | 19.3582 |
| 0.4 | 0.6163 | 28.4568 |
| 1 | 0.4849 | 43.7025 |
| 2 | 0.3718 | 56.8273 |
| 5 | 0.2982 | 65.3681 |
| 10 | 0.2292 | 73.3889 |
| 15 | 0.0722 | 91.7203 |

| Log[Conc.] (log10 µM) | % Inhibition |
|---|---|
| -1.000 | 13.0443 |
| -0.698 | 19.3582 |
| -0.398 | 28.4568 |
| 0.000 | 43.7025 |
| 0.301 | 56.8273 |
| 0.699 | 65.3681 |
| 1.000 | 73.3889 |
| 1.176 | 91.7203 |

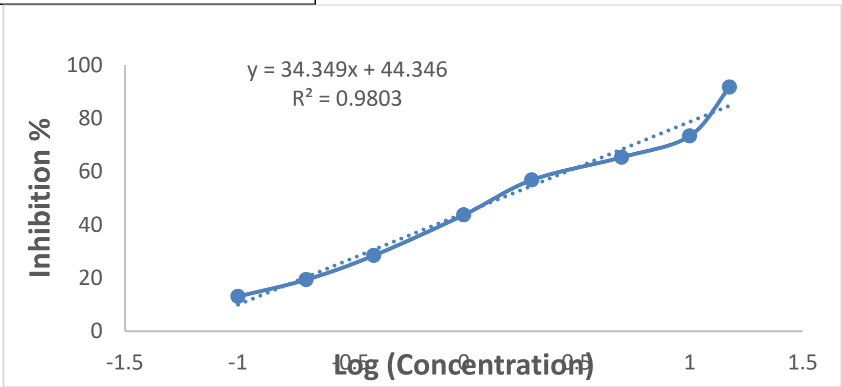


**Compound 3b**

**$IC_{50}$ = 0.462 µM**

| Concentration (µM) | Reading 1 | Reading 2 | Mean Absorbance |
|---|---|---|---|
| 0.1 | 0.7359 | 0.7341 | 0.7350 |
| 0.2 | 0.6926 | 0.6938 | 0.6932 |
| 0.4 | 0.5824 | 0.5839 | 0.5832 |
| 1 | 0.3872 | 0.3887 | 0.3879 |
| 2 | 0.2926 | 0.2915 | 0.2921 |
| 5 | 0.1683 | 0.1672 | 0.1678 |
| 10 | 0.0946 | 0.0934 | 0.0940 |
| 15 | 0.0163 | 0.0171 | 0.0167 |

| Concentration (µM) | Mean Absorbance | % Inhibition |
|---|---|---|
| 0.1 | 0.7350 | 17.4883 |
| 0.2 | 0.6932 | 22.2427 |
| 0.4 | 0.5832 | 34.5914 |
| 1 | 0.3879 | 56.4985 |
| 2 | 0.2921 | 67.2332 |

| Concentration (µM) | Mean Absorbance | % Inhibition |
|---|---|---|
| 5 | 0.1678 | 81.1786 |
| 10 | 0.0940 | 89.4549 |
| 15 | 0.0167 | 98.2066 |

| Log[Conc.] (log10 µM) | % Inhibition |
|---|---|
| -1.000 | 17.4883 |
| -0.698 | 22.2427 |
| -0.398 | 34.5914 |
| 0.000 | 56.4985 |
| 0.301 | 67.2332 |
| 0.699 | 81.1786 |
| 1.000 | 89.4549 |
| 1.176 | 98.2066 |

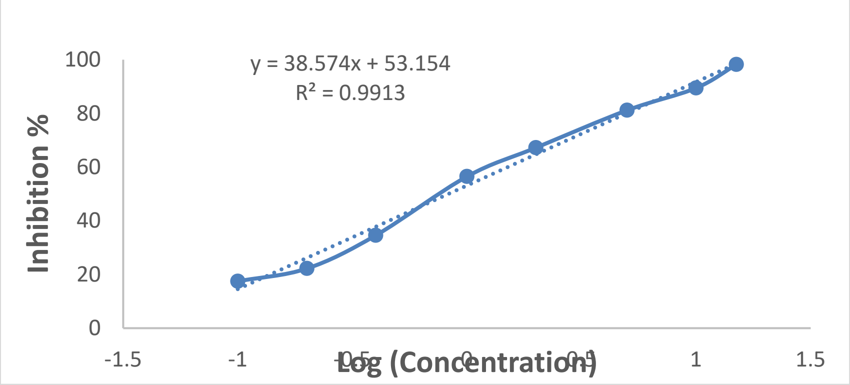


**Compound Zileuton**

**$IC_{50}$ = 0.689 µM**

| Concentration (µM) | Reading 1 | Reading 2 | Mean Absorbance |
|---|---|---|---|
| 0.1 | 0.7362 | 0.7350 | 0.7356 |
| 0.2 | 0.6945 | 0.6927 | 0.6936 |
| 0.4 | 0.6113 | 0.6098 | 0.6106 |
| 1 | 0.4721 | 0.4704 | 0.4712 |
| 2 | 0.3326 | 0.3339 | 0.3333 |
| 5 | 0.2085 | 0.2071 | 0.2078 |
| 10 | 0.1167 | 0.1179 | 0.1173 |
| 15 | 0.0410 | 0.0422 | 0.0416 |

| Concentration (µM) | Mean Absorbance | % Inhibition |
|---|---|---|
| 0.1 | 0.7356 | 15.8404 |
| 0.2 | 0.6936 | 20.6584 |
| 0.4 | 0.6106 | 30.1287 |
| 1 | 0.4712 | 46.1017 |
| 2 | 0.3333 | 61.8763 |
| 5 | 0.2078 | 76.2296 |
| 10 | 0.1173 | 86.5740 |
| 15 | 0.0416 | 95.3966 |

| Log[Conc.] (log10 µM) | % Inhibition |
|---|---|
| -1.000 | 15.8404 |
| -0.698 | 20.6584 |
| -0.398 | 30.1287 |
| 0.000 | 46.1017 |
| 0.301 | 61.8763 |
| 0.699 | 76.2296 |
| 1.000 | 86.5740 |
| 1.176 | 95.3966 |

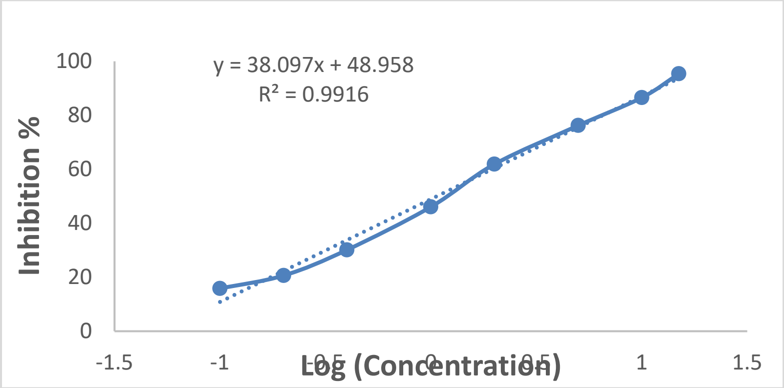

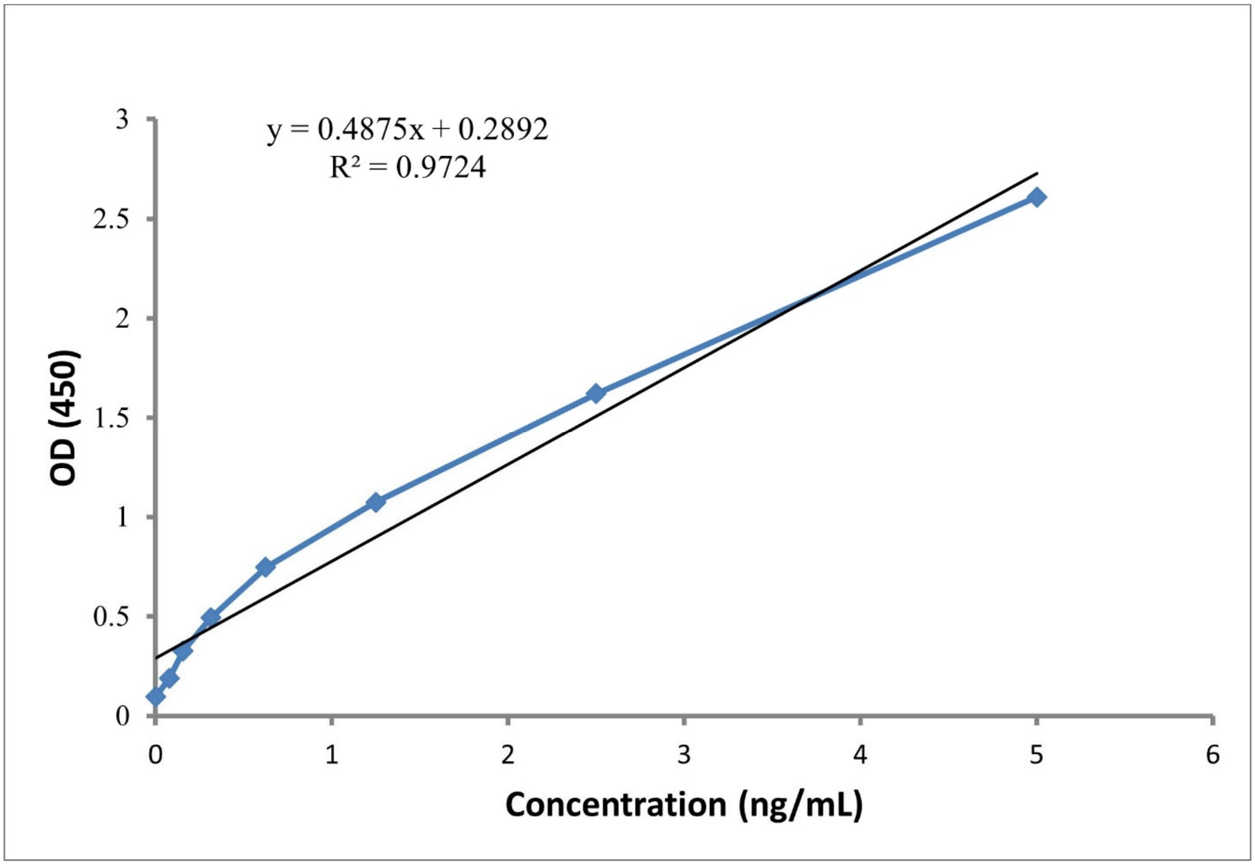

y = 0.4875x + 0.2892
R² = 0.9724
OD (450)
Concentration (ng/mL)
0
0.5
1
1.5
2
2.5
3
0
1
2
3
4
5
6


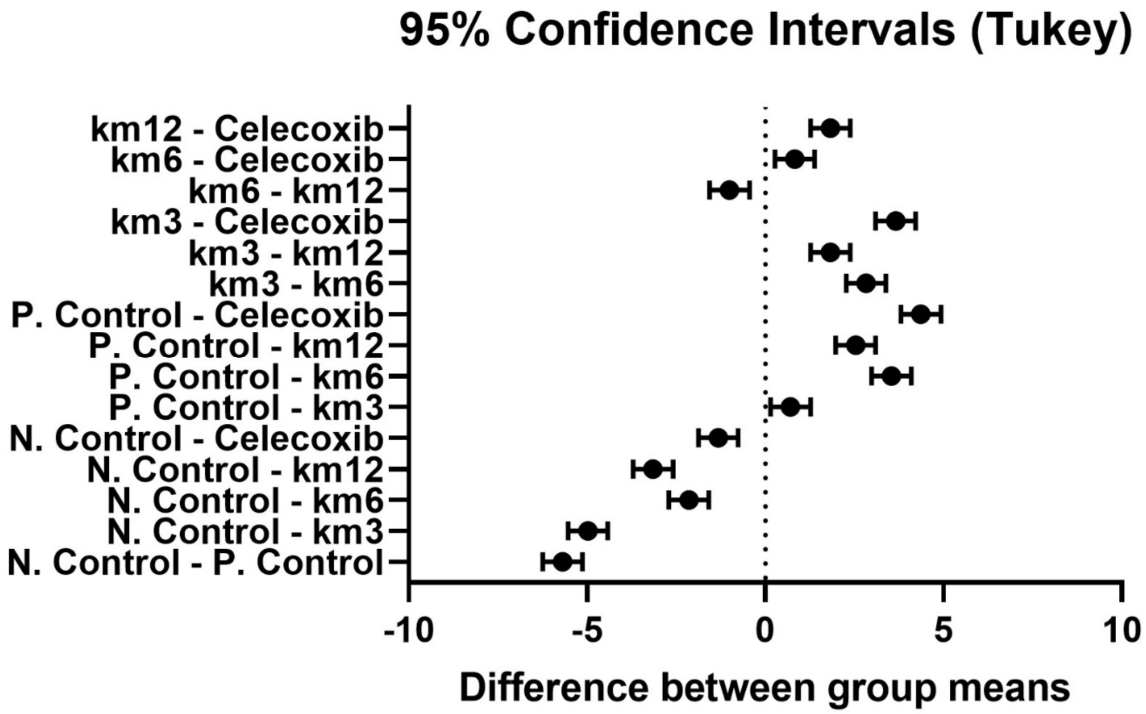

95% Confidence Intervals (Tukey)
km12 - Celecoxib
km6 - Celecoxib
km6 - km12
km3 - Celecoxib
km3 - km12
km3 - km6
P. Control - Celecoxib
P. Control - km12
P. Control - km6
P. Control - km3
N. Control - Celecoxib
N. Control - km12
N. Control - km6
N. Control - km3
N. Control - P. Control
-10
-5
0
5
10
Difference between group means

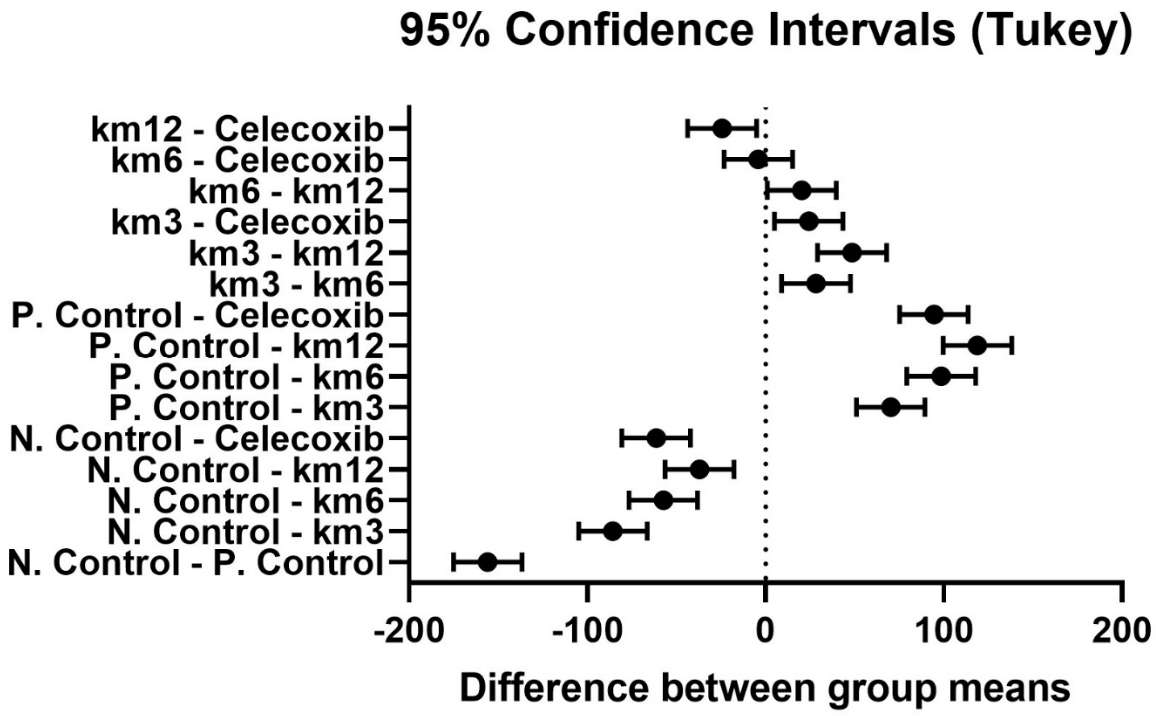
95% Confidence Intervals (Tukey)
km12 - Celecoxib
km6 - Celecoxib
km6 - km12
km3 - Celecoxib
km3 - km12
km3 - km6
P. Control - Celecoxib
P. Control - km12
P. Control - km6
P. Control - km3
N. Control - Celecoxib
N. Control - km12
N. Control - km6
N. Control - km3
N. Control - P. Control
-200
-100
0
100
200
Difference between group means

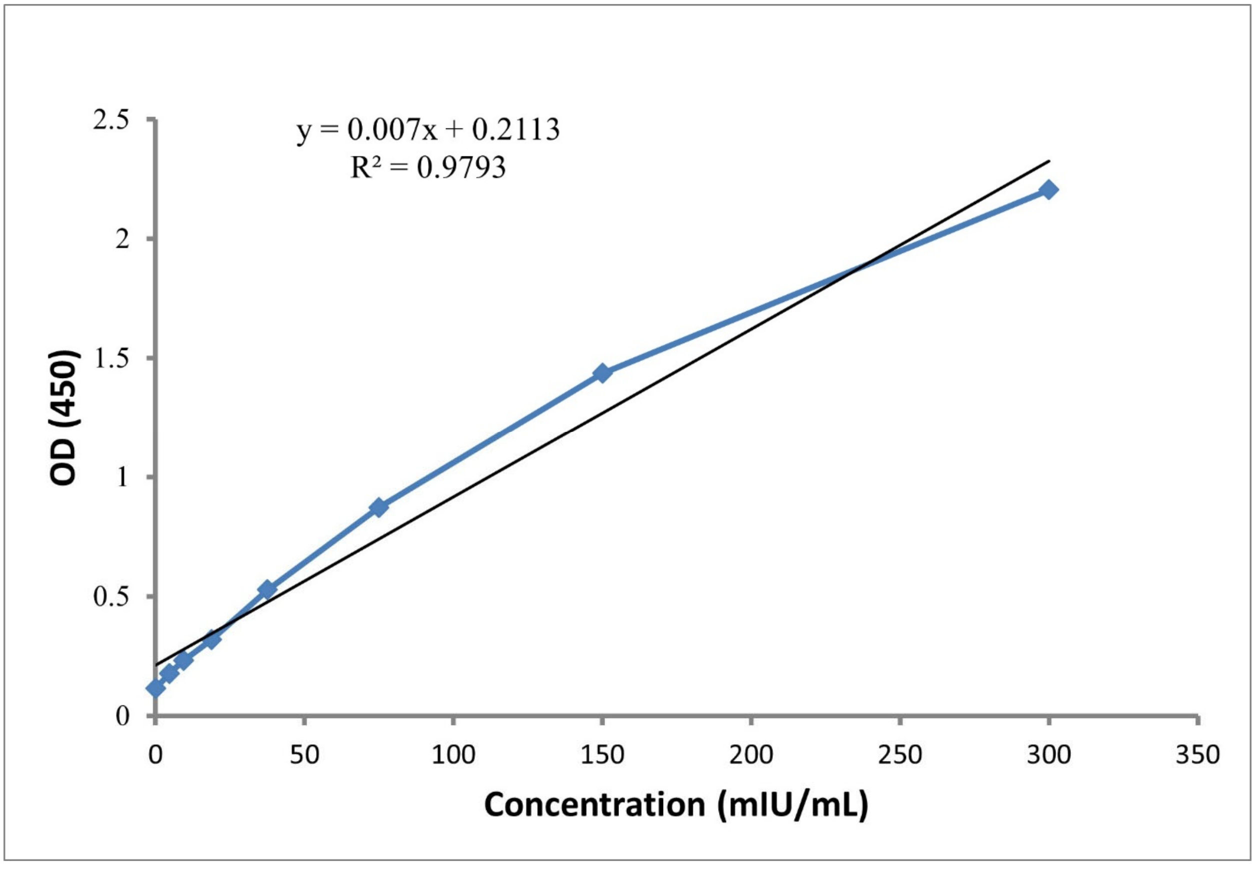
y = 0.007x + 0.2113
R² = 0.9793
OD (450)
0
0.5
1
1.5
2
2.5
0
50
100
150
200
250
300
350
Concentration (mIU/mL)

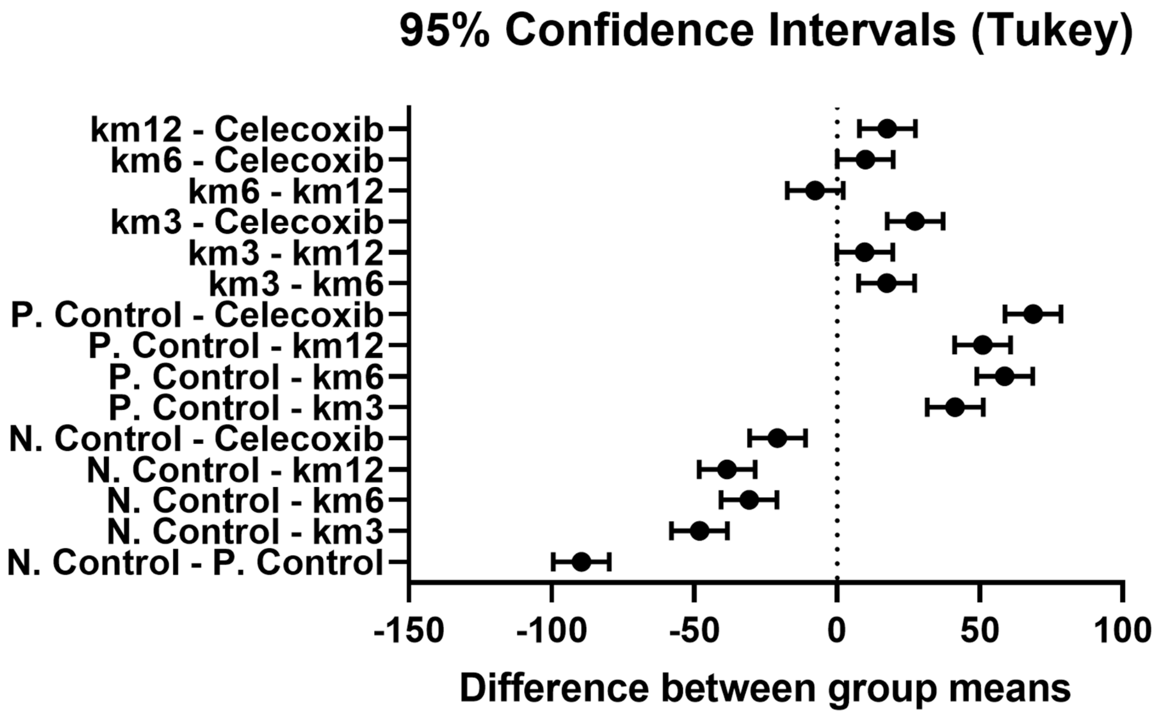
95% Confidence Intervals (Tukey)
km12 - Celecoxib
km6 - Celecoxib
km6 - km12
km3 - Celecoxib
km3 - km12
km3 - km6
P. Control - Celecoxib
P. Control - km12
P. Control - km6
P. Control - km3
N. Control - Celecoxib
N. Control - km12
N. Control - km6
N. Control - km3
N. Control - P. Control
-150
-100
-50
0
50
100
Difference between group means

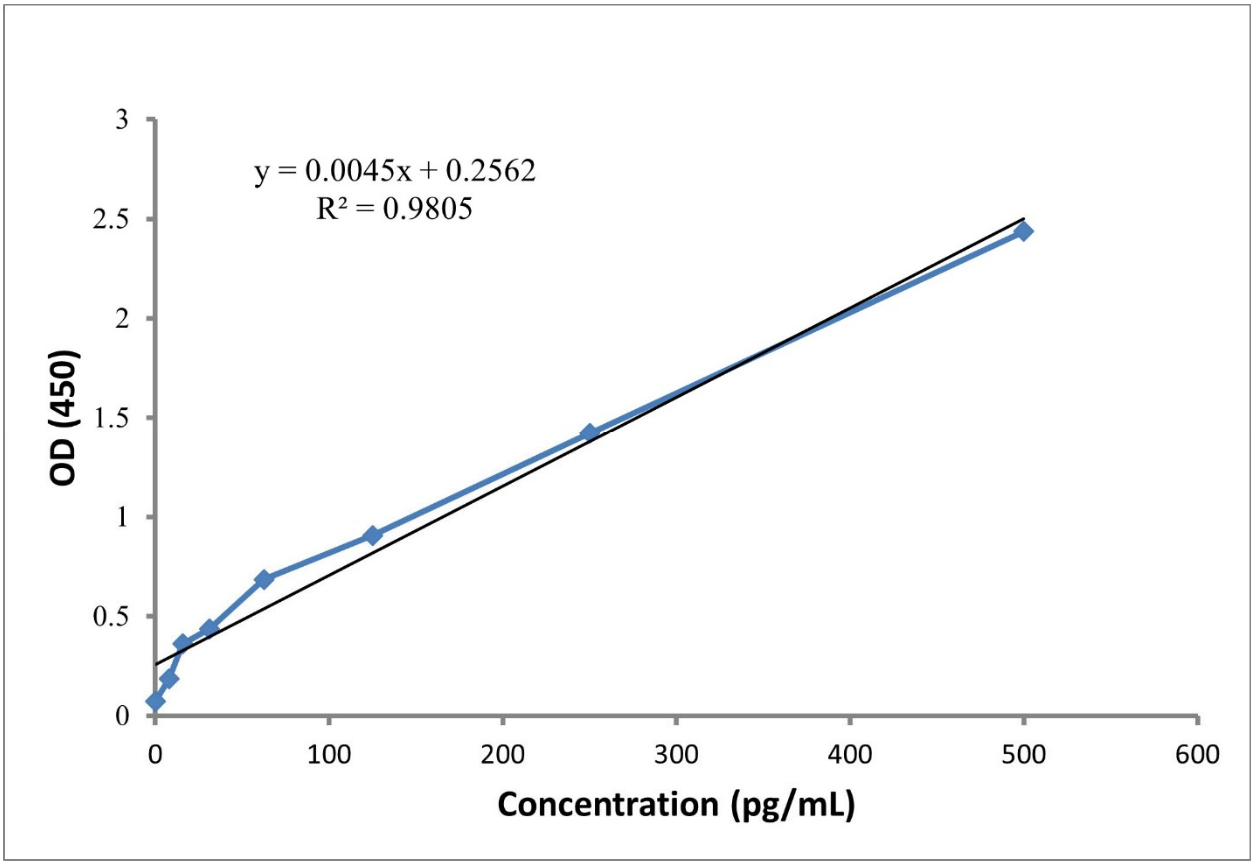
y = 0.0045x + 0.2562
R² = 0.9805
OD (450)
0
0.5
1
1.5
2
2.5
3
0
100
200
300
400
500
600
Concentration (pg/mL)
